# Reporting characteristics and quality of randomized controlled trial protocols in traditional Chinese medicine: a cross-sectional study

**DOI:** 10.3389/fphar.2024.1389808

**Published:** 2024-06-07

**Authors:** Lin Zhang, Han Li, Lihan Hu, Xiangqin Ou, Hanzhi Tan, Xuanqi Zhang, Chung Tai Lau, Aiping Lyu, Zhaoxiang Bian, Xuan Zhang

**Affiliations:** ^1^ Tianjin University of Traditional Chinese Medicine, Tianjin, China; ^2^ Chinese EQUATOR Centre, Hong Kong Baptist University, Kowloon, China; ^3^ Guangdong Provincial Hospital of Chinese Medicine in Guizhou, Guizhou, China; ^4^ School of Chinese Medicine, Hong Kong Baptist University, Kowloon, China; ^5^ Vincent V.C. Woo Chinese Medicine Clinical Research Institute, Hong Kong Baptist University, Kowloon, China; ^6^ Centre for Chinese Herbal Medicine Drug Development, School of Chinese Medicine, Hong Kong Baptist University, Kowloon, China

**Keywords:** evidence synthesis, randomized controlled trial, reporting quality, SPIRIT guideline, traditional Chinese medicine registration: open science framework

## Abstract

**Objectives:**

The impact of the Standard Protocol Items: Recommendations for Interventional Trials of Traditional Chinese Medicine (SPIRIT-TCM) Extension 2018 statement on the reporting quality of randomized controlled trial (RCT) protocols in traditional Chinese medicine (TCM) is not clear. This review aimed to assess the reporting characteristics and quality of RCT protocols involving interventions such as Chinese herbal medicine formulas (CHMF), acupuncture, and moxibustion published in the last 3 years.

**Methods:**

We conducted an extensive search among multiple databases, including All EBM Reviews, Allied and Complementary Medicine (AMED), Embase, Ovid MEDLINE(R), PubMed, Web of Science, Google Scholar, and ClinicalTrials.gov for publications in English from 1 January 2020 to 10 August 2023. Two reviewers independently assessed the eligibility of the publications, extracted predetermined information, and evaluated the reporting based on the SPIRIT-TCM Extension 2018 checklist.

**Results:**

Of the 420 eligible protocols (comprising 163 studies on CHMF, 239 on acupuncture, and 18 on moxibustion), the average reporting compliance rate was only 35.4%. Approximately half of the assessed items fell into the category of poorly reported, demonstrating a compliance rate below 65%. Notably, reporting compliance in acupuncture and moxibustion interventional studies exhibited higher scores than compliance in CHMF studies.

**Conclusion:**

Continued, concerted, and coordinated efforts are required by journals, editors, reviewers, and investigators to improve the application and promotion of the SPIRIT-TCM Extension 2018 reporting guideline.

## 1 Introduction

Numerous countries have increasingly recognized the potential value of traditional Chinese medicine (TCM) in preventing and treating both infectious and non-infectious diseases. In the realm of infectious diseases, artemisinin, derived from TCM, is widely employed to treat malaria in Africa (Daily et al., 2022). Addressing the global infectious disease, Coronavirus Disease 2019-related pneumonia, the lung cleansing and detoxifying decoction has demonstrated efficacy in alleviating symptoms like fever, cough, headache, and severity of illness rates (Liu et al., 2020). In the context of non-communicable diseases, arsenic, extracted from TCM, transformed the outlook for acute monocytic leukemia, previously deemed incurable, and is now recommended as a first-line drug (Burnett et al., 2015; Wang et al., 2018). Notably, TCM was incorporated into the International Statistical Classification of Diseases and Related Health Problems by the World Health Organization in 2019 (Lam et al., 2019). Despite these advancements, TCM faces scrutiny regarding its efficacy and safety in Western countries because it is an empirical medicine with complex herbal compositions.

Randomized controlled trials (RCTs) are widely regarded as the gold standard for assessing drug efficacy, particularly in TCM-related trials. To ensure the quality of RCTs at the design stage, the SPIRIT (Standard Protocol Items: Recommendations for Interventional Trials) statements were introduced in 2013. Comprising 33 items, including research background, objectives, and participant information, SPIRIT 2013 serves as a guideline for the minimum content of a clinical trial protocol, aiming to establish routine standards for completeness and transparency (Chan et al., 2013). As a result, the quality of RCT protocols has improved since the publication of the SPIRIT statement. Scholars have observed a significant enhancement in reporting quality by comparing the checklist items of 300 protocols before and after introducing the SPIRIT statement (Tan et al., 2020). This underscores the positive impact of standardized guidelines on promoting transparency and completeness in clinical trial protocols.

Despite a sharp increase in the number of TCM clinical trials registered on ClinicalTrials.gov after the publication of the SPIRIT statement, concerns persisted regarding transparency and repeatability. This is attributed to the fact that the suggestions outlined in the statement are not fully applicable to the distinctive characteristics of TCM (Cheng et al., 2017; Dai et al., 2019; Zhang et al., 2020). Several reporting guidelines for TCM, including acupuncture, moxibustion, and CHMF interventional studies, were developed in 2010 (MacPherson et al., 2010), 2013 (Cheng et al., 2013), and 2018 (Dai et al., 2019), respectively to address this issue. These efforts aimed to provide more comprehensive standards and enhance the quality of RCTs in TCM research. After that, the SPIRIT-TCM Extension 2018 was issued (Dai et al., 2019). Several critical factors were included in this extension, such as TCM pattern, criteria for practitioners, and various TCM interventions.

The introduction of SPIRIT-TCM Extension 2018 marked a significant advancement in the sophistication of research programs for RCTs in TCM. It not only guides the implementation of TCM protocols but also facilitates other scholars in retracing and building upon these protocols. For instance, following SPIRIT-TCM Extension 2018, some researchers reported more comprehensive details, including TCM pattern, quality control and safety assessment of CHMF interventions, and TCM-relevant rationale, in their clinical trial of TCM treatments for irritable bowel syndrome (Zheng et al., 2021). This underscores the positive impact of tailored guidelines on enhancing the rigor and transparency of TCM clinical trials.

Despite the publication of the SPIRIT-TCM Extension 2018 5 years ago, there remains a noticeable absence of detailed analyses regarding its impact on TCM clinical trial protocols. Additionally, there is a scarcity of systematic studies addressing this issue. Previous research has highlighted the inadequate utilization and endorsement of the CONSORT extensions for TCM reporting guidelines (Zhang et al., 2022). To bridge this gap, our objective is to scrutinize a selection of TCM RCT protocols from the past 3 years, with a particular focus on CHMF, acupuncture, and moxibustion—areas covered by the SPIRIT-TCM Extension 2018 guideline. The primary aim of this review is to pinpoint potential shortcomings for enhancement, thereby offering valuable insights to foster greater awareness, application, and dissemination of the SPIRIT-TCM 2018 guideline.

## 2 Materials and methods

This review is conducted and reported according to the Preferred Reporting Items for Systematic Reviews and Meta-Analyses (PRISMA) 2020 checklist (Page et al., 2021). The study has been registered on the Open Science Framework (https://osf.io/573c2/).

### 2.1 Eligibility criteria

The inclusion criteria comprised RCT protocols published in English from 1 January 2020 to 10 August 2023. The inclusion criteria were the following: i) The interventions were limited to CHMF, acupuncture, and moxibustion. According to the CONSORT-2017-CHMF (Gagnier et al., 2006a; Gagnier et al., 2006b; Cheng et al., 2017), the CHMF dosage form included decoctions, granules, powders, and botanical hybrid preparations (Panossian et al., 2024). The administration routes of CHMF were oral and external. ii) The protocol of RCTs. iii) There must be a control group, but no limitations were imposed on the types of control groups or the assessed outcomes. iv) Published in English including all EBM Reviews (Cochrane Database of Systematic Reviews, American College of Physicians Journal Club, Database of Abstracts of Reviews of Effects, Cochrane Clinical Answers, Cochrane Methodology Register, Health Technology Assessments, Cochrane Central Register of Controlled Trials and NHS Economic Evaluation Database), AMED (Allied and Complementary Medicine), Embase, Ovid MEDLINE(R), PubMed, Web of Science, Google scholar, and ClinicalTrials.gov.

The exclusion criteria were the following: i) the final results of an RCT or review; ii) duplicated records; iii) without studied interventions; iv) lack of a control group; v) complex intervention (with more than two studied interventions).

The systematic searches were conducted across the electronic databases, as noted in the detailed search strategy for each database provided in Supplementary Material S1.

### 2.2 Screening

Two reviewers (HL and LHH) independently assessed the publications during two phases. The initial phase involved the evaluation of titles and abstracts, while the subsequent phase entailed a comprehensive assessment of the complete texts of selected studies. The inclusion and exclusion criteria were consistently applied throughout both phases. Throughout the entire process, two reviewers conducted a meticulous verification to ensure the utmost precision. Any disagreements between the reviewers were resolved through discussion or consultation with the third reviewer (XZ).

### 2.3 Data extraction

A pre-designed data extraction form with four parts and 16 specific items was developed (Supplementary Material S2). Extracted data mainly addressed two categories: i) general characteristics (e.g., publication year, information of authors, journal, and funding), and ii) TCM-related characteristics (e.g., studied disease with or without TCM patterns and TCM-related outcomes). Before formally commencing the extraction process, a third reviewer (XZ) conducted training sessions on the extraction form rules. Moreover, a pilot test was administered on nine randomly selected articles, covering all three interventions of CHMF, acupuncture, and moxibustion. Two independent reviewers (LHH and HZT) were responsible for extracting data from each article. Any disagreements between the reviewers were resolved through discussion.

### 2.4 Assessment of reporting quality

In the SPIRIT-TCM Extension 2018 checklist, 15 items were customized for interventions involving CHMF, acupuncture, and moxibustion, respectively (Dai et al., 2019). These items included study rationale, eligibility criteria, intervention details, and outcome indicator measures. To enhance the efficiency of the assessment process, each item in SPIRIT-TCM Extension 2018 has been redesigned into one or more questions to facilitate more complex assessments. Thus, a specially designed quality assessment form was developed by the researcher (XZ) based on the SPIRIT-TCM Extension 2018 checklist. The formulation of the questions was primarily guided by the distinctive features inherent in TCM intervention modalities, such as i) essential elements of TCM in clinical practice; ii) revealing the TCM utilization of individualization in a transparent manner, elucidating the underlying reasons for its implementation, and providing comprehensive insights into its operational intricacies; and iii) the rationale for the selection of TCM. The assessment form utilized in this study incorporates the 15 primary items outlined in SPIRIT-TCM Extension 2018. Specifically, it encompasses 29 sub-questions for CHMF intervention, 21 sub-questions for acupuncture intervention, and 20 sub-questions for moxibustion intervention (Table 1-3). It was notable that item 6a.3 is not applicable for assessment as this review excluded complex interventions.

**TABLE 1:** Twenty-nine sub-questions for CHMF interventional studies based on SPIRIT-TCM Extension 2018.

No.	Section/topic	Extension items	Questions for assessment
1	Title	1a Specify the patient population in terms of 1) a WM-defined disease, 2) a WM-defined disease with a specific TCM pattern, or 3) a TCM pattern	Q1. Whether the diseases or patterns were accurately and specifically reported in the title
		1b Specify the intervention in terms of 1) CHMF, 2) acupuncture, 3) moxibustion, or 4) other TCM therapy(s)	Q2. Whether the specific intervention was reported in the title
2	Background and rationale	6a.1 Provide the background and rationale of the research question with TCM theory	Q3. Whether the rationale of TCM about the CHMF intervention for diseases or TCM patterns was reported in the background/introduction
		6a.2 Describe the rationale of the utilized TCM interventions with references	Q4. Whether the rationale for CHMF intervention was reported in the background/introduction
		6b Describe the rationale and principle(s) for selecting comparators corresponding to certain interventions (i.e., CHMFs, acupuncture, moxibustion, or other TCM interventions), considering 1) comparable with tested intervention; 2) success of blinding	Q5. Whether the rationale and principle(s) for selecting comparators corresponding to the CHMF intervention were reported
3	Objectives	7 State the objectives or hypotheses regarding the specific TCM intervention for 1) a WM-defined disease, 2) a WM-defined disease with a specific TCM pattern, or 3) a TCM pattern	Q6. Whether the objectives or hypotheses regarding the CHMF intervention were reported
4	Eligibility criteria	10a State whether participants with a specific TCM pattern will be recruited in terms of 1) diagnostic criteria and 2) inclusion and exclusion criteria. All criteria utilized should be universally recognized, or reference(s) where detailed explanations can be found should be given	Q7. If participants with a specific TCM pattern were recruited, whether the TCM diagnostic criteria, inclusion and exclusion criteria, and reference(s) were reported
		10b Descriptions of the roles, qualifications, and other relevant experience of the researchers (e.g., participant screeners, care providers, outcome assessors, and data analysts) in TCM research are recommended	Q8. Whether the roles, qualifications, and other relevant experience of the researchers were reported
		10c Descriptions of the qualification and relevant experience of the study center(s) involved in a TCM trial are recommended	Q9. Whether the qualifications and relevant experience of the study center(s) involved in a TCM trial were described
5	Interventions	11a.1 Interventions for the experimental group(s) with sufficient detail to allow replication	Q10. Whether the name of the CHMF and each medical substance were reported
			Q11. Whether the source of the CHMF was reported
			Q12. Whether the source and processing method of the CHMF were reported
			Q13. Whether the dosage form, production method, and administration route of the CHMF were reported
			Q14. Whether the dosage of CHMF and each medical substance were reported
			Q15. Whether the reference(s) to the dosage of CHMF was reported
			Q16. Whether the administration route of the CHMF was reported
			Q17. Whether the quality control of each ingredient and the whole formula(s) and the safety assessment of the whole formula(s) were conducted
			Q18. For a protocol with individualized CHMFs, whether the study reported how, when, and by whom the CHMF was modified
			Q19. For a protocol with patent proprietary CHMFs, whether the name and dosage of the formula were reported
			Q20. For a protocol with patent proprietary CHMFs, whether the efficacy of the formula was reported
			Q21. For a protocol with patent proprietary CHMFs, whether the safety assessment and quality control of the formula were reported
			Q22. For a protocol with patent proprietary CHMFs, whether the details of the formula were illustrated
			Q23. Whether the patent-proprietary CHMF utilized in the protocol is identical to the publicly available reference
		11a.2 Describe interventions for the control group(s) with sufficient detail to allow replication	Q24. For a protocol with placebo control, whether the name and dosage of each ingredient, the similarity of placebo with the intervention (e.g., color, smell, taste, appearance, packaging), the quality control and safety assessment of placebo, the administration route, regimen, and dosage and the production information of placebo, including when, where, how, and by whom the placebo was produced were reported
			Q25. For a protocol with an active control, if a CHMF will be used, refer to the recommendations of 11a.1A; if a chemical agent will be used, whether the name, administration route, dosage, and regime were reported
		11d.2 Descriptions of other interventions that will be administrated to experimental and/or control groups are recommended (e.g., rescue interventions), with enough details to allow replication	Q26. Whether the details of other interventions administered to experimental and/or control groups were reported
6	Outcomes	12a Provide the rationale of TCM-related indexes as outcomes (e.g., the change of degree and scope of symptoms and signs related to pattern differentiation)	Q27. Whether the rationale of TCM-related indexes as outcomes was reported
		12b Provide the details of the TCM-related outcomes assessment, including i) the measuring methods and standard (e.g., frequency, severity rating scale of symptoms and signs, verified pattern questionnaire, and time points for assessment and corresponding rationale), ii) assessor qualification (e.g., relevant assessment experience and years in clinical practice), iii) methods used to enhance the quality of assessment (e.g., multiple repeated observation and training of assessors), and iv) related reference(s)	Q28. Whether the details of the TCM-related outcomes assessment were described
7	Data collection methods	18a When a trial targeting a TCM pattern or a WM-defined disease with a specific TCM pattern, baseline data about the TCM pattern should be provided	Q29. If the trial targeted a TCM pattern or a WM-defined disease with a specific TCM pattern, whether the baseline data about the TCM pattern were provided

**TABLE 2:** Twenty-one sub-questions for acupuncture interventional studies based on SPIRIT-TCM Extension 2018.

No.	Section/topic	Extension items	Questions for assessment
1	Title	1a Specify the patient population in terms of 1) a WM-defined disease, 2) a WM-defined disease with a specific TCM pattern, or 3) a TCM pattern	Q1. Whether the diseases or patterns were accurately and specifically reported in the title
		1b Specify the intervention in terms of 1) CHMF, 2) acupuncture, 3) moxibustion, or 4) other TCM therapy(s)	Q2. Whether the specific intervention was reported in the title
2	Background and rationale	6a.1 Provide the background and rationale of the research question with TCM theory	Q3. Whether the TCM rationale about acupuncture intervention for diseases or TCM patterns was reported in the background/introduction
		6a.2 Describe the rationale of the utilized TCM interventions with references	Q4. Whether the rationale for acupuncture intervention was reported in the background/introduction
		6b Describe the rationale and principle(s) for selecting comparators corresponding to certain interventions (i.e., CHMFs, acupuncture, moxibustion, or other TCM interventions), considering 1) comparable with the tested intervention; 2) success of blinding	Q5. Whether the rationale and principle(s) for selecting comparators corresponding to acupuncture intervention were reported
3	Objectives	7 State the objectives or hypotheses regarding the specific TCM intervention for 1) a WM-defined disease, 2) a WM-defined disease with a specific TCM pattern, or 3) a TCM pattern	Q6. Whether the objectives or hypotheses regarding acupuncture intervention were reported
4	Eligibility criteria	10a State whether participants with a specific TCM pattern will be recruited in terms of 1) diagnostic criteria and 2) inclusion and exclusion criteria. All criteria utilized should be universally recognized, or reference(s) where detailed explanations can be found should be given	Q7. If participants with a specific TCM pattern were recruited, whether the TCM diagnostic criteria, inclusion and exclusion criteria, and reference(s) were reported
		10b Descriptions of the roles, qualifications, and other relevant experience of the researchers (e.g., participant screeners, care providers, outcome assessors, and data analysts) in TCM research are recommended	Q8. Whether the roles, qualifications, and other relevant experience of the researchers were reported
		10c Descriptions of the qualifications and relevant experience of study center(s) involved in a TCM trial are recommended	Q9. Whether the qualifications and relevant experience of study center(s) involved in a TCM trial were described
5	Interventions	11a.1 Interventions for the experimental group(s) with sufficient detail to allow replication	Q10. Whether the treatment environment and participant posture were reported
			Q11. Whether the number of needle insertions per subject per session (mean and range if possible) and names and locations of acupoints (uni/bilateral) were reported
			Q12. Whether the angle and depth of insertion were reported
			Q13. Whether the response sought from participants (e.g., de qi or muscle twitch response) and the needle stimulation (e.g., manual, electrical) were described
			Q14. Whether the needle type (e.g., diameter, length, and manufacturer or material) was described
			Q15. Whether the number of acupuncture treatment sessions, the frequency and duration of acupuncture treatment sessions, and the needle retention time were reported
		11a.2 Describe interventions for the control group(s) with sufficient detail to allow replication	Q16. If the study protocol was the blank/waitlist control, whether any special arrangements in pre-treatment, treatment, and post-treatment periods corresponding to the experimental intervention were reported
			Q17. If the study protocol was sham acupuncture or acupuncture-like control, whether the comparability of the sham acupuncture or acupuncture-like control and the comprehensive details for the recommendations of Intervention 11a.1B were reported
		11d.2 Descriptions of other interventions that will be administrated to experimental and/or control groups are recommended (e.g., rescue interventions), with enough details to allow replication	Q18. Whether the details of other interventions administered to experimental and/or control groups were reported
6	Outcomes	12a Provide the rationale of TCM-related indexes as outcomes (e.g., the change of degree and scope of symptoms and signs related to pattern differentiation)	Q19. Whether the rationale for selecting TCM-related indexes as outcomes was reported
		12b Provide the details of the TCM-related outcomes assessment, including i) the measuring methods and standard (e.g., frequency, severity rating scale of symptoms and signs, verified pattern questionnaire, and time points for assessment and corresponding rationale), ii) assessor qualification (e.g., relevant assessment experience and years in clinical practice), iii) methods used to enhance the quality of assessment (e.g., multiple repeated observation and training of assessors), and iv) related reference(s)	Q20. Whether the details of the TCM-related outcomes assessment were described
7	Data collection methods	18a When trial targeting TCM pattern or a WM-defined disease with a specific TCM pattern, baseline data about the TCM pattern should be provided	Q21. If the trial targeted a TCM pattern or a WM-defined disease with a specific TCM pattern, whether the baseline data about the TCM pattern were provided

**TABLE 3:** Twenty sub-questions for moxibustion interventional studies based on SPIRIT-TCM Extension 2018.

No.	Section/topic	Extension items	Questions for assessment
1	Title	1a Specify the patient population in terms of 1) a WM-defined disease, 2) a WM-defined disease with a specific TCM pattern, or 3) a TCM pattern	Q1. Whether the diseases or patterns were accurately and specifically reported in the title
		1b Specify the intervention in terms of 1) CHMF, 2) acupuncture, 3) moxibustion, or 4) other TCM therapy(s)	Q2. Whether the specific intervention was reported in the title
2	Background and rationale	6a.1 Provide the background and rationale of the research question with TCM theory	Q3. Whether the TCM rationale for moxibustion intervention for diseases or TCM patterns was reported in the background/introduction
		6a.2 Describe the rationale of the utilized TCM interventions with references	Q4. Whether the rationale for moxibustion intervention was reported in the background/introduction?
		6b Describe the rationale and principle(s) for selecting comparators corresponding to certain interventions (i.e., CHMFs, acupuncture, moxibustion, or other TCM interventions), considering 1) comparable with tested intervention; 2) success of blinding	Q5. Whether the rationale and principle(s) for selecting comparators corresponding to moxibustion intervention were reported
3	Objectives	7 State the objectives or hypotheses regarding the specific TCM intervention for 1) a WM-defined disease, 2) a WM-defined disease with a specific TCM pattern, or 3) a TCM pattern	Q6. Whether the objectives or hypotheses regarding moxibustion intervention were reported
4	Eligibility criteria	10a State whether participants with a specific TCM pattern will be recruited in terms of 1) diagnostic criteria and 2) inclusion and exclusion criteria. All criteria utilized should be universally recognized, or reference(s) where detailed explanations can be found should be given	Q7. If participants with a specific TCM pattern were recruited, whether the TCM diagnostic criteria, inclusion and exclusion criteria, and reference(s) were reported
		10b Descriptions of the roles, qualifications, and other relevant experience of the researchers (e.g., participant screeners, care providers, outcome assessors, and data analysts) in TCM research are recommended	Q8. Whether the roles, qualifications, and other relevant experience of the researchers were reported
		10c Descriptions of the qualification and relevant experience of study center(s) involved in a TCM trial are recommended	Q9. Whether the qualifications and relevant experience of study center(s) involved in a TCM trial were described
5	Interventions	11a.1 Interventions for the experimental group(s) with sufficient detail to allow replication	Q10. Whether the patient posture during the moxibustion treatment and the treatment environment were reported
			Q11. Whether the name and number (uni/bilateral) of acupoints/locations used for moxibustion were reported
			Q12. Whether the moxibustion procedures and responses sought from participants (e.g., warm feeling, skin reddening, burning pain, heat-sensitization phenomenon, etc.) were reported
			Q13. Whether the materials used for moxibustion were reported
			Q14. Whether the number, frequency, and duration of the moxibustion sessions were reported
		11a.2 Describe interventions for the control group(s) with sufficient detail to allow replication	Q15. If the study protocol used a blank/waitlist control, whether any special arrangements in pre-treatment, treatment, and post-treatment periods corresponding to the experimental intervention were reported
			Q16. If the study protocol was sham moxibustion or moxibustion-like control, whether the comparability of the sham moxibustion or moxibustion-like control and the comprehensive details for the recommendations of Intervention 11a.1B were reported
		11d.2 Descriptions of other interventions that will be administrated to the experimental and/or control groups are recommended (e.g., rescue interventions), with enough details to allow replication	Q17. Whether the details of other interventions administered to the experimental and/or control groups were reported
6	Outcomes	12a Provide the rationale of TCM-related indexes as outcomes (e.g., the change of degree and scope of symptoms and signs related to pattern differentiation)	Q18. Whether the rationale for using TCM-related indexes as outcomes was reported
		12b Provide the details of the TCM-related outcomes assessment, including i) the measuring methods and standards (e.g., frequency, severity rating scale of symptoms and signs, verified pattern questionnaire, and time points for assessment and corresponding rationale), ii) assessor qualification (e.g., relevant assessment experience and years in clinical practice), iii) methods used to enhance the quality of assessment (e.g., multiple repeated observation and training of assessors), and iv) related reference(s)	Q19. Whether the details of the TCM-related outcomes assessment were described
7	Data collection methods	18a When trial targeting TCM pattern or a WM-defined disease with a specific TCM pattern, baseline data about the TCM pattern should be provided	Q20. If the trial targeted a TCM pattern or a WM-defined disease with a specific TCM pattern, whether the baseline data about the TCM pattern were provided

To facilitate quality assessment, each question offers four scoring options: 2, 1, 0, and Not Applicable (NA). In the assessment process, each question will be awarded a score of “2” only if all relevant details of the information specified in SPIRIT-TCM Extension 2018 are fully reported. Conversely, a score of “1” was awarded if only some of the required information was disclosed, and a score of “0” was awarded if no information was disclosed at all. Cases marked “NA” indicate that the particular item or sub-question is not relevant to the particular RCT. Details of the standard operating procedures (SOP), including the original items and specific questions, scoring rules, and examples, are presented in Supplementary Material S3. All reviewers were trained by a senior reviewer (XZ) and participated in pilot evaluations of ten randomly selected articles from each type of intervention to ensure consistency in scoring. Each question was scored by two independent reviewers (e.g., CHMF articles: HZT and LHH, acupuncture articles: HL and LHH, and moxibustion articles: HZT and HL). Cohen’s kappa was used to identify the level of agreement, including poor agreement (0%–40%), moderate agreement (41%–60%), substantial agreement (61%–80%), and almost complete agreement (81%–100%) (Viera and Garrett, 2005). Any inconsistencies were resolved by the senior reviewer (XZ).

### 2.5 Data analysis

Descriptive statistics were performed to analyze the characteristics and reporting compliance of the included articles. Categorical variables are summarized in frequencies and percentages. For the reported compliance rate (CR, calculated as CR = n/N*100%), the count of “Not Applicable” instances was excluded from the calculation, while continuous variables are expressed as mean and standard deviation (SD). To assess the reporting quality, each item or question was evaluated based on the number of reports that received a score of “2” out of the total number of included reports, and the compliance rate was calculated according to the proportion. The compliance level was then categorized into three groups: excellent compliance (>90%), good compliance (between 65% and 90%), and poor compliance (<65%). The report compliance rate for each intervention was calculated separately by time of publication and by specific items. Data analysis was performed using SPSS software, version 26.0, and the results were clearly demonstrated using figures to illustrate trends and comparisons. All data were collected and recorded in Microsoft Office Excel 365.

## 3 Results

### 3.1 Search

**FIGURE 1:**
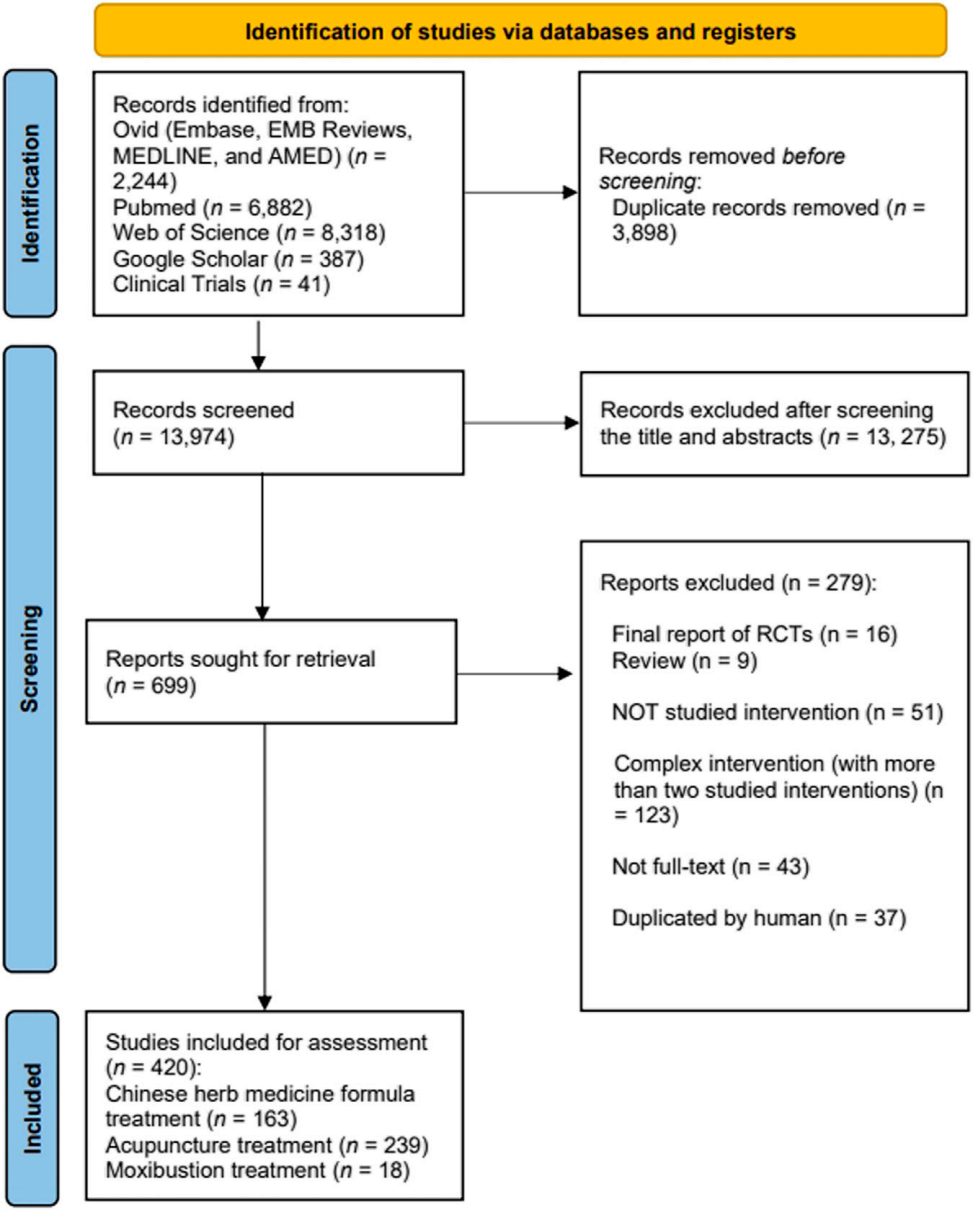
Overall flow chart of the search and selection process.

The initial search yielded 17,872 records, from which 17,452 relevant studies were retained after excluding duplicates and screening the titles and abstracts. Following a thorough review of full-text articles, we identified 420 eligible protocols, including 163 Chinese herbal medicine formula studies, 239 acupuncture studies, and 18 moxibustion studies (Figure 1). The RCTs included in this analysis are listed in Supplementary Material S4.

### 3.2 Characteristics of included studies

This study included 420 protocols published from 2020 to 2023. All were published in English journals, of which 17.9% had an impact factor greater than 3. The corresponding authors of all studies were distributed in 12 countries, and 89.5% of the corresponding authors were from China. Other countries included South Korea, the United States, France, Germany, Portugal, Brazil, the United Kingdom, Australia, Canada, and Poland. Only 4.3% of the participants were identified as having a Western medicine (WM)-defined disease(s) with a specific TCM pattern(s), while the remaining 95.7% were only identified as having a WM-defined disease(s). The most commonly studied disease was the disease of the nervous system (14.8%). In terms of TCM pattern(s) types, 0.4% were patterns of qi stagnation and blood stasis. In addition, 17.4% of the studies used TCM-related indicators in the outcomes. Funding support was reported for 94.5% of the protocols. However, only 2.1% of the roles of funding were described. More details are provided in Table 4 and Supplementary Material S5.

**TABLE 4:** Characteristics of included studies (n = 420).

Characteristic	CHMF n (%)	Acupuncture n (%)	Moxibustion n (%)	Total n (%)
Part 1 Information about included articles, journals, and corresponding authors				
Year of publications				
2020	57 (13.6)	66 (15.7)	6 (1.4)	129 (30.7)
2021	41 (9.8)	78 (18.6)	1 (0.2)	120 (28.6)
2022	52 (12.4)	63 (15)	8 (1.9)	123 (29.3)
2023	13 (3.1)	32 (7.6)	3 (0.7)	48 (11.4)
Journal type^a^				
English journal (SCIE), with impact factor >3	33 (7.9)	40 (9.5)	2 (0.5)	75 (17.9)
Distributions of corresponding authors (top 3)^b^				
China	149 (35.5)	209 (49.8)	18 (4.3)	376 (89.5)
South Korea	9 (2.1)	8 (1.9)	0 (0)	17 (4)
United States	1 (0.2)	6 (1.4)	0 (0)	7 (1.7)
Part 2 Participants				
Type of participants				
Participants with a WM-defined disease(s)	152 (36.2)	236 (56.2)	14 (3.3)	402 (95.7)
A WM-defined disease(s) with the specific TCM pattern(s)	11 (2.6)	3 (0.7)	4 (1.0)	18 (4.3)
Type of disease(s)/symptom(s) (top 3)^c^				
Diseases of the nervous system	14 (3.3)	43 (10.2)	5 (1.2)	62 (14.8)
Diseases of the circulatory system	39 (9.3)	9 (2.1)	4 (1.0)	52 (12.4)
Diseases of the musculoskeletal system or connective tissue	8 (1.9)	35 (8.3)	5 (1.2)	48 (11.4)
Type of TCM pattern(s) (top 3)^d^				
Qi stagnation and blood stasis syndrome	2 (0.4)	0 (0)	0 (0)	2 (0.4)
Blood stasis syndrome	0 (0)	1 (0.2)	0 (0)	1 (0.2)
Qi deficiency and blood stasis syndrome	0 (0)	1 (0.2)	0 (0)	1 (0.2)
Part 3 Outcomes				
TCM-related outcome(s)				
Included TCM pattern(s) outcome(s)	60 (14.3)	8 (1.9)	5 (1.2)	73 (17.4)
Part 4 Funding and roles				
Included funding supports	155 (36.9)	225 (53.6)	17 (4.0)	397 (94.5)
Included roles of funding	7 (1.7)	2 (0.5)	0 (0)	9 (2.1)

^a^ The journal types and impact factors of English journals were based on the latest data on the official website of Journal Citation Reports. Detailed rules are presented in Supplementary Material 5.1.

^b^ The total number of countries (413) was less than the number of included studies (420) because 10 studies did not identify a corresponding author, and multiple corresponding authors from different countries were listed in three articles. The percentage was based on the number of included studies, 420. More details of other countries are presented in Supplementary Material 5.2.

^c^ According to the International Classification of Diseases 11th Revision (ICD-11) in 2023. More details are presented in Supplementary Material 5.3.

^d^ This item is based on the 18 studies that reported the type of TCM pattern(s). The percentage was based on the number of included studies (420). More details are presented in Supplementary Material 5.4.

### 3.3 Reporting quality assessment of all included protocols

**FIGURE 2:**
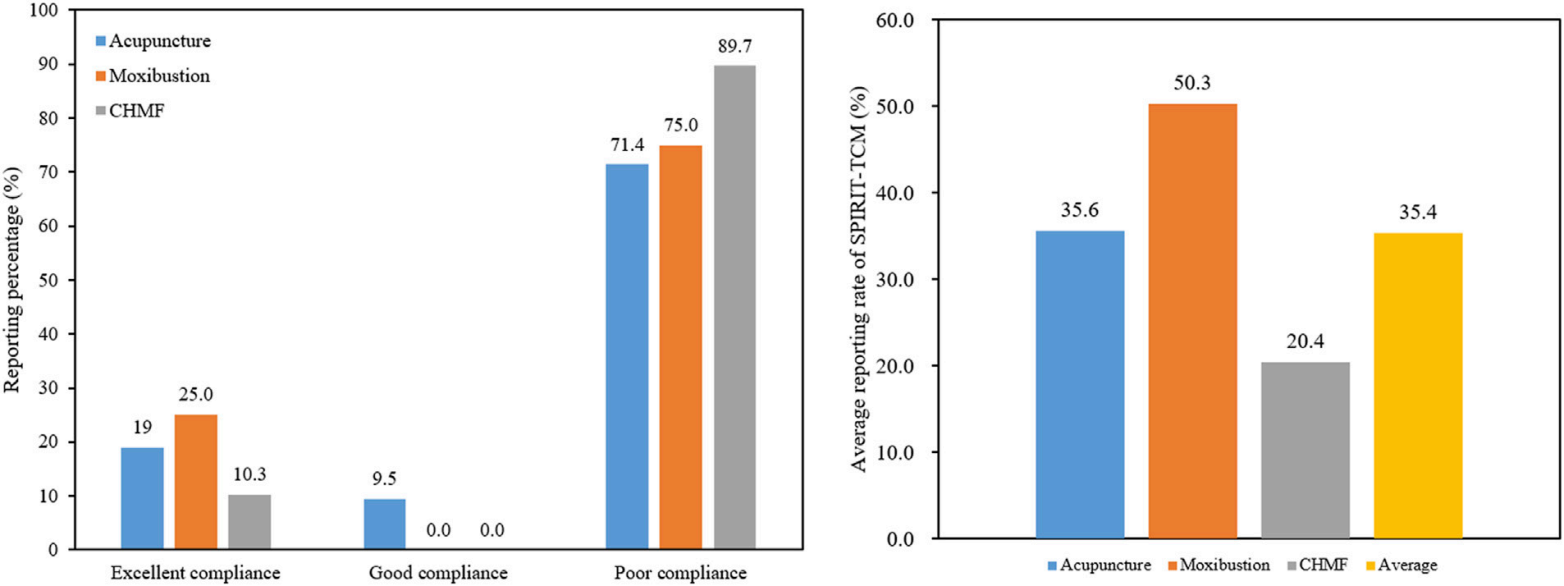
Level of reporting compliance of the included studies.

**FIGURE 3:**
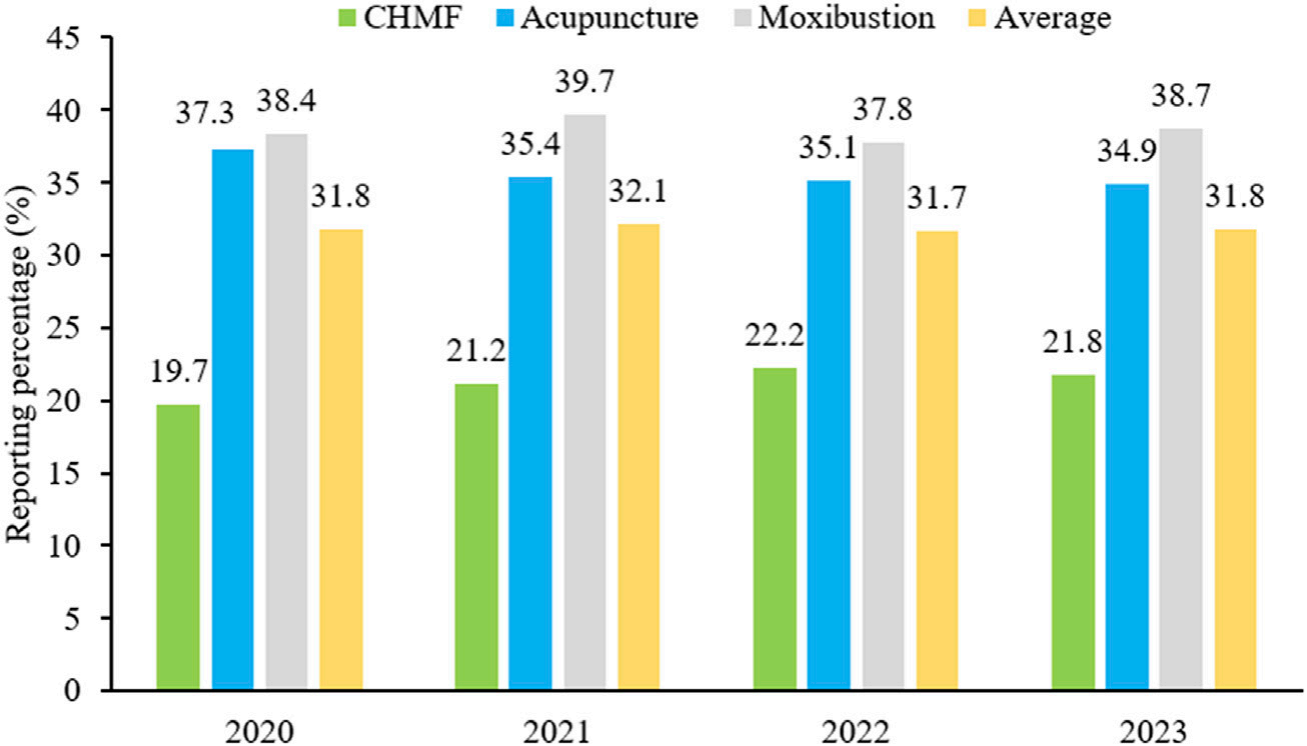
Reporting percentages by included years.

**FIGURE 4:**
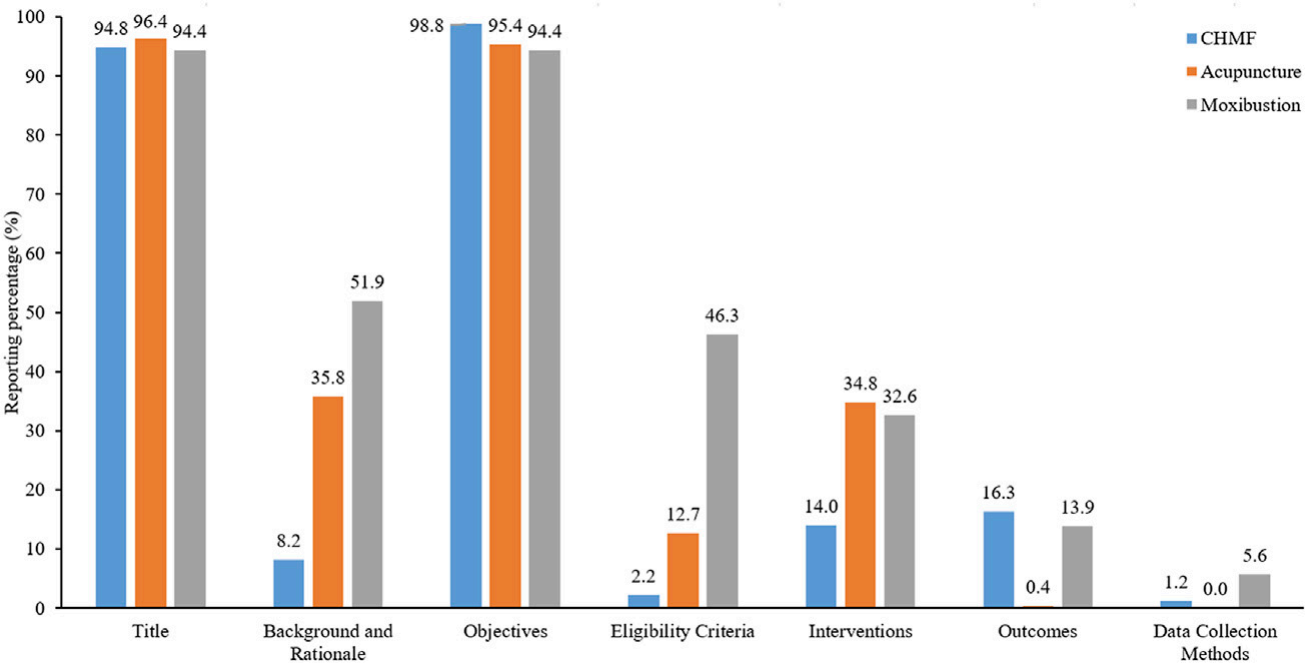
Reporting percentage of different sections in the reporting checklists.

SPIRIT-TCM Extensions 2018 was used to evaluate the quality of 420 articles that met the inclusion criteria, with an average reporting rate of 35.4%. Among the specific interventions, moxibustion demonstrated the highest reporting rate at 50.3%, followed by acupuncture at 35.6% and CHMF at 20.4%. The inter-rater agreement for each item exceeded 85% among the two reviewers (Supplementary Material S6). Concerning the compliance level, protocols of moxibustion were categorized as demonstrating excellent compliance, while CHMF protocols displayed poor compliance (Figure 2). In terms of year of publication, the average reporting rates from 2020 to 2023 were 31.8%, 32.1%, 31.7%, and 31.8%. Among the three interventions, moxibustion had the highest average annual reporting rate, and CHMF had the lowest (Figure 3). Focusing on specific items, the average reporting rate of each intervention in title and objectives exhibited a relatively higher rate above 90%. Conversely, the average compliance rate for eligibility criteria, outcomes, and data collection methods was below 30%, particularly for data collection methods, standing at a mere 2.3%. For acupuncture intervention, no literature report on this item considered TCM patterns in the collection of baseline data or data statistics (Figure 4).

### 3.4 Reporting quality assessment of CHMF protocols

**FIGURE 5:**
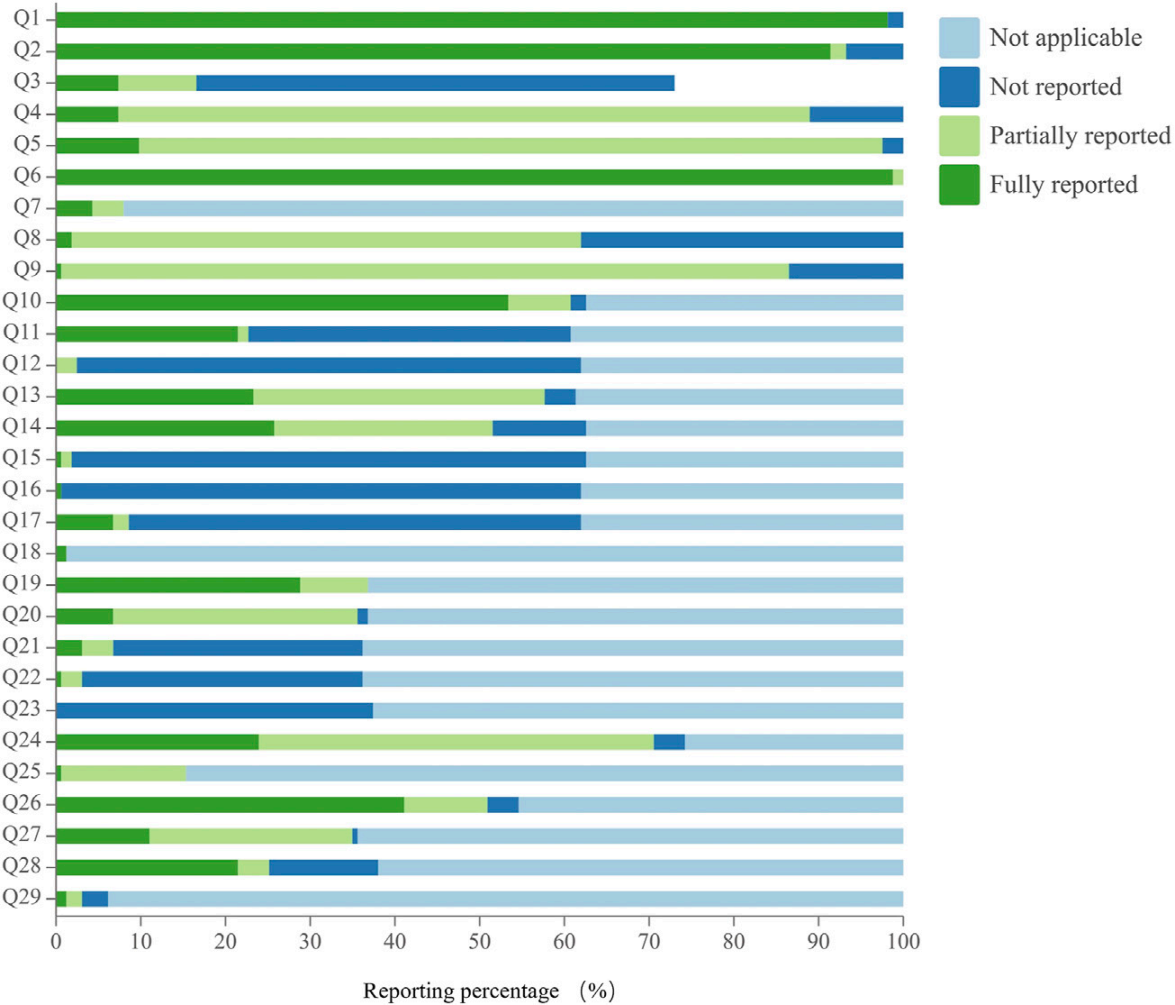
Reporting quality assessment of CHMF protocols.

The reporting quality of CHMF studies assessed using the SPIRIT-TCM extension 2018 checklist varied across 29 specific questions, with excellent compliance observed in 10.3% of cases, good compliance in 0%, and poor compliance in 89.7%. Regarding poor reporting, there were 26 specific issues assessed, and their reporting rates were ranked in descending order as follows: Q10 (53.4%), Q26 (41.1%), Q20 (28.8%), Q19 (28.8%), Q14 (25.8%), Q24 (23.9%), Q13 (23.3%), Q28 (21.5%), Q11 (21.5%), Q27 (11.0%), Q5 (9.8%), Q4 (7.4%), Q3 (7.4%), Q17 (6.7%), Q7 (4.3%), Q21 (3.1%), Q8 (1.8%), Q29 (1.2%), Q18 (1.2%), Q25 (0.6%), Q22 (0.6%), Q16 (0.6%), Q15 (0.6%), Q9 (0.6%), Q23 (0%), and Q12 (0%). Notably, the reporting rates for 24 of these specific issues were below 30%, especially for principles of CHMF, individualized treatment, details of the control, relevant experience of study center(s), details of patent proprietary CHMF, reference(s) to dosage of CHMF, administration route, origin and processing method of the CHMF, and whether the patent proprietary CHMF utilized in the protocol was identical to the public applicable reference. Details are shown in Figure 5 and Supplementary Material S7.

### 3.5 Reporting quality assessment of acupuncture protocols

**FIGURE 6:**
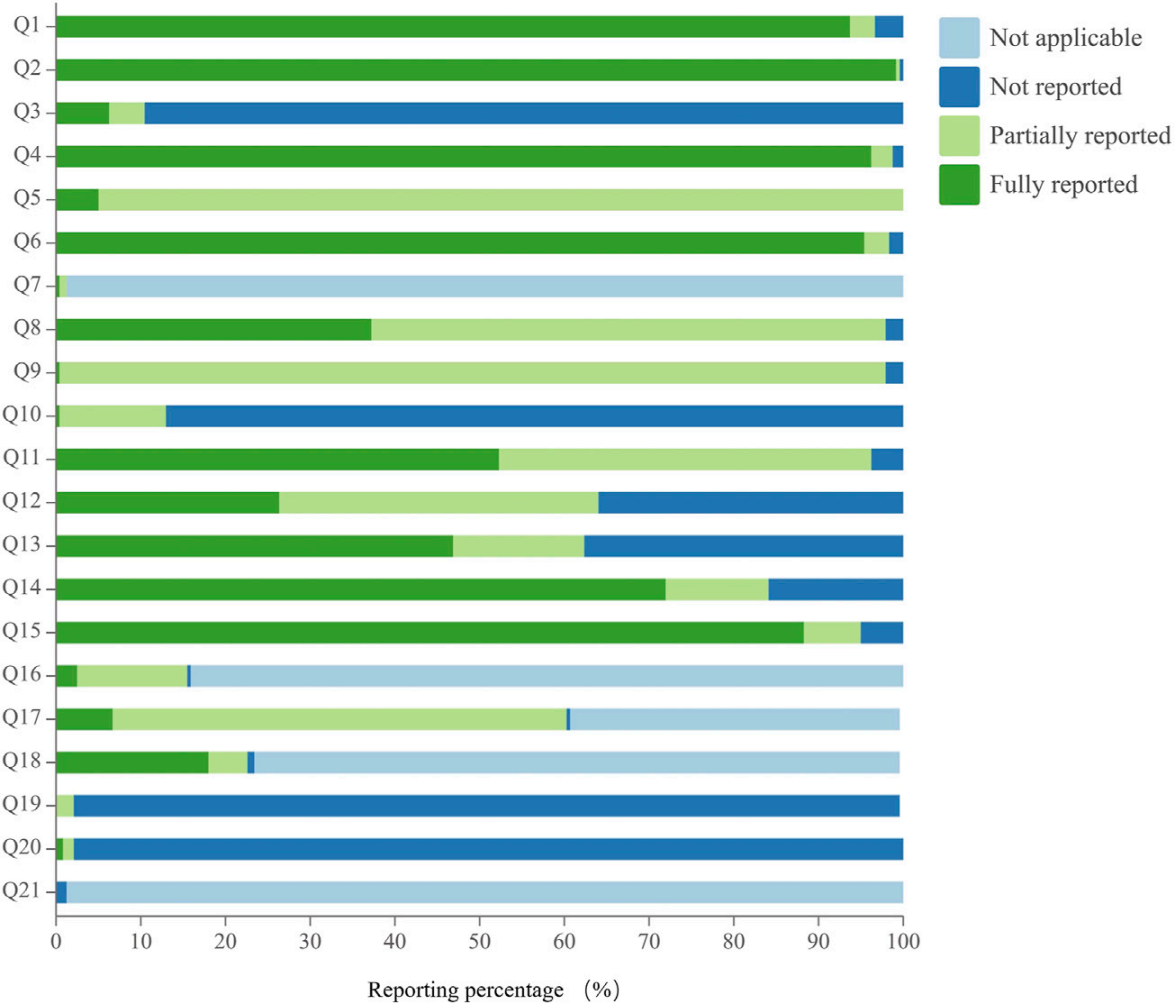
Reporting quality assessment of acupuncture protocols.

The reporting quality of acupuncture studies assessed using the SPIRIT-TCM checklist varied across 21 specific questions, with excellent compliance observed in 19.0% of cases, good compliance in 9.5%, and poor compliance in 71.4%. Regarding poor reporting, there were 15 specific issues assessed, and their reporting rates were ranked in descending order as follows: Q11 (52.3%), Q13 (46.9%), Q8 (37.2%), Q12 (26.4%), Q18 (18.0%), Q17 (6.7%), Q3 (6.3%), Q5 (5.0%), Q16 (2.5%), Q20 (0.8%), Q9 (0.4%), Q7 (0.4%), Q10 (0.4%), Q21 (0%), and Q19 (0%). Notably, the reporting rates for 12 of these specific issues were below 30%, especially for angle and depth of insertion, qualification and relevant experience of study center(s), TCM diagnostic criteria, inclusion and exclusion criteria, and reference(s), treatment environment and participant posture, rationale of TCM-related indexes as outcomes, and data collection methods. Details are shown in Figure 6 and Supplementary Material S8.

### 3.6 Reporting quality assessment of moxibustion protocols

**FIGURE 7:**
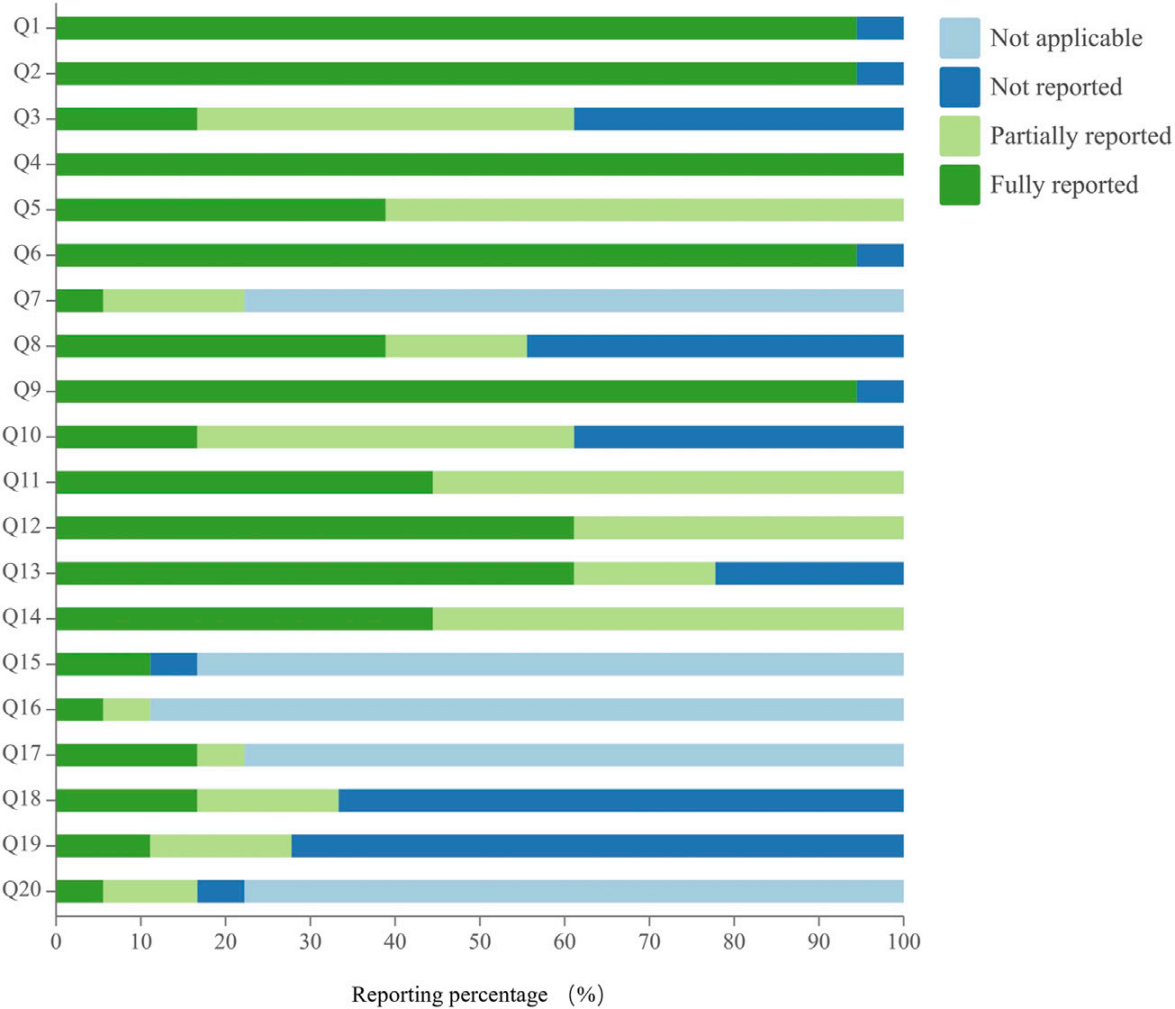
Reporting quality assessment of moxibustion protocols.

The reporting quality of moxibustion studies assessed using the SPIRIT-TCM checklist varied across 20 specific questions, with excellent compliance observed in 25.0% of cases, good compliance in 0%, and poor compliance in 75.0%. Regarding poor reporting, there were 15 specific issues assessed, and their reporting rates were ranked in descending order as follows: Q12 (61.1%), Q13 (61.1%), Q11 (44.4%), Q14 (44.4%), Q5 (38.9%), Q8 (38.9%), Q18 (16.7%), Q17 (16.7%), Q10 (16.7%), Q3 (16.7%), Q19 (11.1%), Q15 (11.1%), Q20 (5.6%), Q16 (5.6%), and Q7 (5.6%). Notably, the reporting rates for nine of these specific issues were below 30%, especially for TCM diagnostic criteria, inclusion and exclusion criteria and reference(s), comparability of the sham moxibustion or moxibustion-like control, details of the intervention, and data collection methods. Details are shown in Figure 7 and Supplementary Material S9.

## 4 Discussion

SPIRIT-TCM Extension 2018 underscored the significance of TCM syndromes in clinical trials by providing examples and explanations for different TCM interventions. Reporting of the protocols included in this item fell short, particularly in either ignoring during the study design or incomplete reporting of the TCM syndrome (if used) information about diagnosis criteria and measurement methods. In clinical practice, TCM places a crucial emphasis on treatment guided by syndrome differentiation, considering it as vital as disease diagnosis. To illustrate, in the context of the evolution of coronavirus disease 2019, different TCM syndromes may manifest, each requiring varied medications. The accurate differentiation of TCM patterns is essential for ensuring clinical efficacy, and an incorrect differentiation may result in ineffective intervention and even adverse effects (Zhao et al., 2020; Zhao et al., 2021). Therefore, it is imperative to highlight the importance of TCM syndrome types, as they not only serve as a guarantee for therapeutic effectiveness but also play a pivotal role in the reproducibility of experiments.

Only 17.4% of the studies adopted TCM-related outcomes. TCM pattern score is the most common type of TCM-related outcome report and can evaluate the composite efficacy of treatments according to a series of symptom evaluations. Generally, the higher the symptom score is, the more severe the disease will be (Jiang et al., 2016). In previous studies, some scholars have also indicated a lack of TCM symptom outcomes in most RCTs of TCM. For instance, a breast cancer-related study (Ji et al., 2020) accesses the TCM clinical value with a series of Western clinical indicators but without TCM symptom indicators. TCM symptom scores focus on subjective feelings and symptoms, while the Western clinical indicators highlight the laboratory report. Sometimes, there is no clear correlation between subjective symptoms and laboratory tests. To illustrate, acute myocardial infarction patients might have severe clinical symptoms but a normal electrocardiograph and laboratory indicators (Zhang et al., 2023). Hence, neglecting TCM-related indicators may introduce bias, potentially yielding inaccurate results. Special consideration should be given to TCM symptom/pattern scores, which not only serve as a crucial metric for emphasizing the overall efficacy of TCM therapies but also serve as a focal point for evaluating the effectiveness or ineffectiveness of a TCM treatment program in alleviating specific symptoms.

SPIRIT-TCM Extension 2018 also placed significant emphasis on providing specific and detailed descriptions of TCM interventions, particularly in CHMF, acupuncture, and moxibustion. Notably, TCM interventions pose a unique challenge for quantification, in contrast to Western medicine. This study, however, reveals a concerning gap in the reporting of details. Specifically, less than 30% of CHMF protocols detail the principles underlying CHMFs, individualized treatment approaches, specific control groups, particulars of patent proprietary CHMFs, references to dosage, and the origin and processing methods of the CHMF. The quality control of Chinese herbs (e.g., the origin and method of preparation) is a key factor in the efficacy. For example, “Dihuang,” Rehmannia, produced in Henan Province, is divided into “raw Dihuang” and “processed Dihuang” (Su et al., 2016) with different functions. The raw Dihuang is mainly used to clear heat and cool the blood, nourish Yin, and promote the production of body fluid, while the processed Dihuang is mainly used to tonify the blood and nourish Yin. The TCM-related RCTs must detail the origin, processing, formula, and so on. The lack of processing information on TCM may lead to unrepeatable results. The reason may be that most first authors are clinicians or statisticians who do not have a background related to TCM (Zhang et al., 2019). Therefore, including professionals with a background in TCM is necessary to ensure quality control of CHMF.

Similarly, less than 30% of the acupuncture protocols provided information on the angle and depth of insertion, qualifications and relevant experience of study center(s), TCM diagnostic criteria, inclusion and exclusion criteria with references, treatment environment specifics, and participant posture. The angle and depth of insertion are the main guarantees of efficacy and safety. For example, in terms of treatment effectiveness, deeper insertion had a better analgesic effect than shallow insertion in the treatment of chronic lumbar myofascial pain (Ceccherelli et al., 2002). In terms of safety, pneumothorax will be triggered by straight thrusts rather than oblique thrusts in the acupuncture of the chest (Nishie et al., 2021). Therefore, the description of the angle and depth of insertion is essential for acupuncture RCTs, but this aspect is not commonly reported in RCTs. The reason that the demerits of the acupuncture RCTs about the angle and depth of insertion may have two sides: On the one hand, for practitioners, different acupuncturists operate the same acupuncture point with different depths and angles; on the other hand, for patients, different patients have different body mass indexes and physical characteristics. Therefore, for the details of the intervention to be better and replicable, the characteristics of the included population (e.g., height, weight, etc.) need to be strictly defined, and the practitioner needs to be tarined in the operation of acupuncture.

In moxibustion, less than 30% of protocols reported the comparability of sham moxibustion or moxibustion-like control and provided detailed intervention information. However, the selection of the comparator will influence the interpretations of trial outcomes according to the SPIRIT-TCM Extension 2018, and the safety and efficacy cannot be proved and will be doubted by Western medicine specialists (Wang et al., 2022). The reason for the absence of sham moxibustion or moxibustion-like controls is that moxibustion, unlike other drugs, cannot be set up in the same way as the test drug in terms of the drug’s color, taste, or smell. For example, it is not only made in various forms (e.g., moxa cone moxibustion, warm needle moxibustion, etc.) but also has a different mechanism of treatment (e.g., the external smoke directly eliminates germs, and the burning temperature reaches 43° to achieve the desired therapeutic effect, and the therapeutic effect of infrared light radiation). Therefore, the moxibustion control group needs to be set up to meet the conditions of smoke, heat, and radiation insulation at the same time. For example, aluminum foil can be utilized for smoke and radiation insulation, and an asbestos heat-resistant layer can be used for heat insulation (Pan et al., 2019).

Among the three typical TCM intervention methods, moxibustion presented the highest reporting rate at 50.3%, followed by acupuncture at 35.6%, and finally, CHMF at 20.4%. Delving into specific items, the average reporting rate in the title and objectives stands relatively high. In contrast, the average reporting rate for eligibility criteria, outcomes, and data collection methods is below 30%, particularly for data collection methods, which is remarkably low at 2.3%. Notably, acupuncture studies exhibited the poorest performance in this regard, particularly in the collection of baseline data or data statistics related to TCM patterns. Similarly, a previous analysis of RCT reports of acupuncture for low back pain between 2010 and 2020 showed that the quality was moderate (Cohen’s κ-statistic 0.4 <κ ≤ 0.6) (Liu et al., 2021). The documentation about the quality of herbal medicine formulas was poor according to the CONSORT checklist in 2006 (Bian et al., 2006)^4^. In addition, a systematic review (Li et al., 2020) of Chinese patent medicine for eczema showed the mean overall quality score was not good with reference to the CONSORT-CHMF 2017 statement.

This study is subject to certain limitations. First, the review focused on identifying TCM RCT protocols published between 1 January 2020 and 10 August 2023 across eight designated databases. Any records beyond the specified cutoff period or not included in these databases were excluded. Additionally, the study only considered publications in English, potentially overlooking trials published in other languages. Second, the quality assessment was strictly based on the SPIRIT-TCM 2018 checklists, limiting the scope to three TCM interventions: CHMF, acupuncture, and moxibustion. When acknowledging these constraints, we believe that the findings presented in this study reflect significant and reliable trends in TCM protocol within the specified parameters.

## 5 Conclusion

This review first set SPIRIT-TCM Extension 2018 as a reference to evaluate the reported characteristics and quality of protocols of RCT that involve interventions such as CHMF, acupuncture, and moxibustion published in the last 3 years and to provide a basis for the refinement of subsequent RCTs in TCM. The results showed that the average compliance rate of TCM RCTs was only 35.4%, with only 4.3% identifying TCM pattern-based relevant diseases, 17.4% of the studies used TCM outcome-related indicators, and all TCM intervention details were described in less than 30% of the studies. Therefore, in the future, the following methods should be adopted to promote the impact and application of the SPIRIT-TCM Extension 2018: 1) In the selection of diseases and interventions, follow the guiding principles of TCM diagnosis and treatment to determine the disease, TCM patterns, treatment principles, and treatment protocols, and strictly determine the inclusion criteria according to the requirements of the diseases; (2) in terms of the details of the intervention, the quality and safety of the CHMF, the operation methods of acupuncture and moxibustion, and the setting of an appropriate control group or placebo group need to be strictly controlled; (3) in terms of outcome indicators, TCM-related indicators are indispensable to highlight the clinical efficacy of TCM.

## Data Availability

All data analysed during this study are included in the article/Supplementary Materials. More information can be found in the registration dataset at Open Science Framework (https://osf.io/573c2/).
