# Supplementary material for: Reporting characteristics and quality of randomized controlled trial protocols in traditional Chinese medicine: a cross-sectional study
Source: Front Pharmacol. 2024 Jun 7;15:1389808. doi: 10.3389/fphar.2024.1389808 (PMC11190156; doi:10.3389/fphar.2024.1389808)
Supplement: Supplementary file 1 [file DataSheet1.PDF]

Supplementary Information

Contents

Supplementary file 1: Search Strategy 2
    1.1 Search Strategy for CHMFs protocols 2
    1.2 Search Strategy for acupuncture protocols 3
    1.3.Search Strategy for moxibustion protocols 5
Supplementary file 2: Rules for Data extraction 7
Supplementary file 3: Standard operating procedures (SOP) for quality assessment 10
    3.1 SOP for quality assessment of CHMFs protocols 10
    3.2 SOP for quality assessment of acupuncture protocols 46
    3.3 SOP for quality assessment of moxibustion protocols 58
Supplementary file 4: List of the included protocols 74
Supplementary file 5: Details of characteristics of included protocols 99
    5.1 Journal type 99
    5.2 Distributions of corresponding authors 99
    5.3 Type of disease/symptom 99
    5.4 Type of TCM pattern(s) 100
Supplementary file 6: The inter-rater agreement rate of quality assessment 102
    6.1 The inter-rater agreement rate of CHMFs quality assessment 102
    6.2 The inter-rater agreement rate of acupuncture quality assessment 102
    6.3 The inter-rater agreement rate of moxibustion quality assessment 103
Supplementary file 7: Reporting quality assessment of CHMFs protocols 104
Supplementary file 8: Reporting quality assessment of acupuncture protocols 109
Supplementary file 9: Reporting quality assessment of moxibustion protocols 113

Supplementary file 1: Search Strategy

1.1 Search Strategy for CHMFs protocols

1.1.1 Selection of English databases via HKBU Ovid

Date： July 27th, 2023

Databases: EBM Reviews - Cochrane Database of Systematic Reviews <2005 to July 18, 2023>,EBM Reviews - ACP Journal Club <1991 to June 2023>,EBM Reviews - Database of Abstracts of Reviews of Effects <1st Quarter 2016>,EBM Reviews - Cochrane Clinical Answers <July 2023>’EBM Reviews - Cochrane Central Register of Controlled Trials <June 2023>,EBM Reviews - Cochrane Methodology Register <3rd Quarter 2012>,EBM Reviews - Health Technology Assessment <4th Quarter 2016>,EBM Reviews - NHS Economic Evaluation Database <1st Quarter 2016>,AMED (Allied and Complementary Medicine) <1985 to July 2023>,Embase <1974 to 2023 July 26>,Ovid MEDLINE(R) ALL <1946 to July 25, 2023>

1 (randomized controlled trial or controlled clinical trial).pt. or randomized.ab. or placebo.ab. or clinical trials as topic.sh. or randomly.ab. or trial.ti.
2 exp animals/ not humans.sh.
3 1 not 2
4 (protocol$ or "study design$" or "trial design$" or "research design$" or "design and methods" or "design and rationale" or "rationale and design").ti.
5 ("Research Design" or "clinical protocols").mp.
6 4 or 5
7 3 and 6
8 (Chinese herbal Medicine or Chinese herb or Chinese patent medicine or drug$ or herb$ or prescription$ or proprietary Chinese medicines or formula or Chinese medicine compound or Powder or Capsule or Decoction or Pill or Pellet or Plaster or Granula).ti,ab,kw.
9 7 and 8
10 limit 9 to english language
11 limit 10 to full text
12 limit 11 to yr="2020 -Current"

1.1.2 Selection of English databases via PubMed

Date： July 27th, 2023

Database and platform: PubMed 1946 to present (via https://pubmed.ncbi.nlm.nih.gov/)

#1

"Randomized controlled trial" [pt] OR "controlled clinical trial" [pt] OR randomized [tiab] OR randomised [tiab] OR placebo [tiab] OR clinical trial as topic [mesh:noexp] OR randomly [tiab] OR trial [ti] NOT (animals [mh] NOT humans [mh])

#2

protocol*[ti] OR study design [ti] OR trial design[ti] OR research design[ti] OR "design and methods"[ti] OR "design and rationale"[ti] OR "rationale and design"[ti] OR Research Design[mh] OR clinical protocols[mh] OR clinical trial protocol[pt]

#3

Chinese herbal Medicine[Title/Abstract] OR Chinese herb[Title/Abstract] OR Chinese patent medicine [Title/Abstract] OR drug$[Title/Abstract] OR herb$[Title/Abstract] OR prescription$[Title/Abstract] OR proprietary Chinese medicines[Title/Abstract] OR formula[Title/Abstract] OR Chinese medicine compound[Title/Abstract] OR Powder[Title/Abstract] OR Capsule[Title/Abstract] OR Decoction[Title/Abstract] OR Pill[Title/Abstract] OR Pellet[Title/Abstract] OR Plaster[Title/Abstract] OR Granula[Title/Abstract]
#4
#1AND#2AND#3
Filters: Full text, English, from 2020 - 2023

1.1.3 Selection of English databases via Web of Science
Date：July 27th, 2023
Database and platform: Web of Science (All databases) 1945 to present (via Clarivate)
#1
TS=((randomized controlled trial or controlled clinical trial or randomised controlled trials or random allocation or clinical trials or clinical trial or placebo) not (animals/ not humans)) and Preprint Citation Index (Exclude – Database)
#2
TS=(protocol$ or "study design$" or "trial design$" or "research design$" or "design and methods" or "design and rationale" or "rationale and design" or "Research Design" or "clinical protocols") and Preprint Citation Index (Exclude – Database)
#3
TS=(Chinese herbal Medicine OR Chinese herb OR Chinese patent medicine OR drug$ OR herb$ OR prescription$ OR proprietary Chinese medicines OR formula OR Chinese medicine compound OR Powder OR Capsule OR Decoction OR Pill OR Pellet OR Plaster OR Granula)
#4
#1 AND #2 AND #3 and Preprint Citation Index (Exclude – Database) and 2020 or 2021 or 2022 or 2023 (Publication Years) and English (Languages)

1.1.4 Selection of English databases via Google scholar
Date：July 28th, 2023
Allintitle: Chinese Medicine protocol "Randomized " -animal
Allintitle: herbal protocol "Randomized " -animal
Allintitle: formula protocol "Randomized " -animal
Time: 2020-2023.

1.1.5 Selection of registration information via ClinicalTrials.gov
Date：August 10, 2023
Filters: Condition or disease: Chinese Medicine;
Study Documents: Study protocols;
Time: 01/01/2020-30/06/2023.

1.2 Search Strategy for acupuncture protocols

1.2.1 Selection of English databases via HKBU Ovid

Date：July 27th, 2023

Databases:EBM Reviews - Cochrane Database of Systematic Reviews <2005 to July 18, 2023>,EBM Reviews - ACP Journal Club <1991 to June 2023>,EBM Reviews - Database of Abstracts of Reviews of Effects <1st Quarter 2016>,EBM Reviews - Cochrane Clinical Answers <July 2023>,EBM Reviews - Cochrane Central Register of Controlled Trials <June 2023>,EBM Reviews - Cochrane Methodology Register <3rd Quarter 2012>,EBM Reviews - Health Technology Assessment <4th Quarter 2016>,EBM Reviews - NHS Economic Evaluation Database <1st Quarter 2016>,AMED (Allied and Complementary Medicine) <1985 to July 2023> Embase <1974 to 2023 July 26>,Ovid MEDLINE(R) ALL <1946 to July 25, 2023>

1 (randomized controlled trial or controlled clinical trial).pt. or randomized.ab. or placebo.ab. or clinical trials as topic.sh. or randomly.ab. or trial.ti.

2 exp animals/ not humans.sh.

3 1 not 2

4 (protocol$ or "study design$" or "trial design$" or "research design$" or "design and methods" or "design and rationale" or "rationale and design").ti.

5 ("Research Design" or "clinical protocols").mp.

6 4 or 5

7 3 and 6

8 (acupuncture or needle or electroacupuncture or electro-acupuncture or auriculoacupuncture or auriculo-acupuncture or acupotomy or acupotomies or Transcutaneous Electric Nerve Stimulation or percutaneous electrical nerve stimulation or acupoint or "acupuncture points" or "warm needling" or "ear acupuncture" or "fire acupuncture" or acupotome).ti,ab.

9 7 and 8

10 limit 9 to english language

11 limit 10 to full text

12 limit 11 to yr="2020 -Current"

1.2.2 Selection of English databases via PubMed

Date：July 27th, 2023

Database and platform: PubMed 1946 to present (via https://pubmed.ncbi.nlm.nih.gov/)

#1

"Randomized controlled trial" [pt] OR "controlled clinical trial" [pt] OR randomized [tiab] OR randomised [tiab] OR placebo [tiab] OR clinical trial as topic [mesh:noexp] OR randomly [tiab] OR trial [ti] NOT (animals [mh] NOT humans [mh])

#2

(protocol*[ti] OR study design [ti] OR trial design[ti] OR research design[ti] OR "design and methods"[ti] OR "design and rationale"[ti] OR "rationale and design"[ti] OR Research Design[mh] OR clinical protocols[mh] OR clinical trial protocol[pt])

#3

acupuncture[Title/Abstract] OR needle[Title/Abstract] OR electroacupuncture[Title/Abstract]

OR electro-acupuncture[Title/Abstract] OR auriculoacupuncture[Title/Abstract] OR auriculo-acupuncture[Title/Abstract] OR acupotomy[Title/Abstract] OR acupotomies[Title/Abstract] OR Transcutaneous Electric Nerve Stimulation[Title/Abstract] OR percutaneous electrical nerve stimulation[Title/Abstract] OR acupoint[Title/Abstract] OR "acupuncture points"[Title/Abstract] OR "warm needling"[Title/Abstract] OR "ear acupuncture"[Title/Abstract] OR "fire acupuncture"[Title/Abstract] OR acupotome[Title/Abstract]
#4
#1AND#2AND#3
Filters: Full text, English, from 2020 - 2023

1.2.3 Selection of English databases via Web of Science
Date：July 27th, 2023
Database and platform: Web of Science (All databases) 1945 to present (via Clarivate)

Database and platform: Web of Science (All databases) 1945 to present (via Clarivate)
#1
TS=((randomized controlled trial or controlled clinical trial or randomised controlled trials or random allocation or clinical trials or clinical trial or placebo) not (animals/ not humans)) and Preprint Citation Index (Exclude – Database)
#2
TS=(protocol$ or "study design$" or "trial design$" or "research design$" or "design and methods" or "design and rationale" or "rationale and design" or "Research Design" or "clinical protocols") and Preprint Citation Index (Exclude – Database)
#3
TS=(acupuncture or needle or electroacupuncture or electro-acupuncture or auriculoacupuncture or auriculo-acupuncture or acupotomy or acupotomies or Transcutaneous Electric Nerve Stimulation or percutaneous electrical nerve stimulation) and Preprint Citation Index (Exclude – Database)
#4 #1 AND #2 AND #3 and Preprint Citation Index (Exclude – Database) and 2020 or 2021 or 2022 or 2023 (Publication Years) and English (Languages)

1.2.4 Selection of English databases via Google scholar
Date：July 28th, 2023
Allintitle: Acupuncture protocol "Randomized " -animal
Time: 2020-2023.

1.2.5 Selection of registration information via ClinicalTrials.gov
Date：August 10, 2023
Filters: Condition or disease: acupuncture;
Study Documents: Study protocols;
Time: 01/01/2020-30/06/2023.

1.3.Search Strategy for moxibustion protocols

1.3.1 Selection of English databases via HKBU Ovid

Date：July 27th, 2023

Databases:EBM Reviews - Cochrane Database of Systematic Reviews <2005 to July 18, 2023>,EBM Reviews - ACP Journal Club <1991 to June 2023>,EBM Reviews - Database of Abstracts of Reviews of Effects <1st Quarter 2016>,EBM Reviews - Cochrane Clinical Answers <July 2023>,EBM Reviews - Cochrane Central Register of Controlled Trials <June 2023>,EBM Reviews - Cochrane Methodology Register <3rd Quarter 2012>,EBM Reviews - Health Technology Assessment <4th Quarter 2016>,EBM Reviews - NHS Economic Evaluation Database <1st Quarter 2016>,AMED (Allied and Complementary Medicine) <1985 to July 2023>,Embase <1974 to 2023 July 26>,Ovid MEDLINE(R) ALL <1946 to July 25, 2023>

1 (randomized controlled trial or controlled clinical trial).pt. or randomized.ab. or placebo.ab. or clinical trials as topic.sh. or randomly.ab. or trial.ti.
2 exp animals/ not humans.sh.
3 1 not 2
4 (protocol$ or "study design$" or "trial design$" or "research design$" or "design and methods" or "design and rationale" or "rationale and design").ti.
5 ("Research Design" or "clinical protocols").mp.
6 4 or 5
7 3 and 6
8 (moxibustion or moxabustion or moxibustion therapy or moxa).ti,ab.
9 7 and 8
10 limit 9 to english language
11 limit 10 to full text
12 limit 11 to yr="2020 -Current"

1.3.2 Selection of English databases via PubMed

Date：July 27th, 2023

Database and platform: PubMed 1946 to present (via https://pubmed.ncbi.nlm.nih.gov/)

#1 ("Randomized controlled trial" [pt] OR "controlled clinical trial" [pt] OR randomized [tiab] OR randomised [tiab] OR placebo [tiab] OR clinical trial as topic [mesh:noexp] OR randomly [tiab] OR trial [ti] NOT (animals [mh] NOT humans [mh])

#2 (protocol*[ti] OR study design [ti] OR trial design[ti] OR research design[ti] OR "design and methods"[ti] OR "design and rationale"[ti] OR "rationale and design"[ti] OR Research Design[mh] OR clinical protocols[mh] OR clinical trial protocol[pt])

#3 moxibustion[Title/Abstract] OR moxabustion[Title/Abstract] OR moxibustion therapy[Title/Abstract] OR moxa[Title/Abstract]

#4 #1AND#2AND#3

Filters: Full text, English, from 2020 - 2023

1.3.3 Selection of English databases via Web of Science

Date：July 27th, 2023

Database and platform: Web of Science (All databases) 1945 to present (via

Clarivate)

#1
TS=((randomized controlled trial or controlled clinical trial or randomised controlled trials or random allocation or clinical trials or clinical trial or placebo) not (animals/ not humans)) and Preprint Citation Index (Exclude – Database)
#2
TS=(protocol$ or "study design$" or "trial design$" or "research design$" or "design and methods" or "design and rationale" or "rationale and design" or "Research Design" or "clinical protocols") and Preprint Citation Index (Exclude – Database)
#3
TS=(moxibustion or moxabustion or moxibustion therapy or moxa) and Preprint Citation Index (Exclude – Database)
#4
#1 AND #2 AND #3 and Preprint Citation Index (Exclude – Database) and 2020 or 2021 or 2022 or 2023 (Publication Years) and English (Languages)

1.3.4 Selection of English databases via Google scholar
Date： July 28th, 2023
Allintitle: moxibustion protocol "Randomized " -animal
Time: 2020-2023.

1.3.5 Selection of registration information via ClinicalTrials.gov
Date： August 10, 2023
Filters: Condition or disease: moxibustion;
Study Documents: Study protocols;
Time: 01/01/2020-30/06/2023.

Supplementary file 2: Rules for Data extraction

No.	Items	Rules for Data extraction
**Part 1**	**Information of included journals, and corresponding authors**	
**1**	Article ID	Extracted Record Number via Endnote 20.
**2**	Study title	Extracted via Endnote 20.
**3**	Year of publication	Extracted via Endnote 20.
**4**	Information of corresponding author(s)	Record the name(s), email(s) and work unit(s) of corresponding author(s). If corresponding author(s) is not reported, "Not Applicable"(NA) will be recorded.<br>If more than one corresponding author is listed in one article, record information above one by one.
**5**	Country of the corresponding author's work unit	Record the country(s) of corresponding author(s)'s work unit(s).The total number of countries will exceed the number of included studies if more than one corresponding author is listed from different countries in some articles.
**6**	Journal name	Extracted via Endnote 20.
**7**	Journal type	The information of English journals is from the Clarivate official website of Journal Citation Reports (https://jcr.clarivate.com/jcr/home). The journals included in our study are classified as Science Citation Index Expanded (SCIE), Social Sciences Citation Index (SSCI), or Emerging Sources Citation Index (ESCI).
**8**	Impact factor (IF) of journal	The same as above. According to the latest data on the official website of JCR when searched in Hong Kong, China in August 2023.
**9**	Year of impact factor	The same as above. According to the latest data on the official website of JCR when searched in Hong Kong, China in August 2023.
**10**	Type of TCMs	Classified as Chinese Herbal Medicine, Acupuncture, Moxibustion.
**Part 2**	**Participants**	
**11**	Name of disease/symptom	Record the name of disease/symptom in the study.
**12**	Type of disease	Diseases reported in the study are classified according to the International Classification of Diseases 11th Revision (ICD-11) (https://icd.who.int/browse11/l-m/en).<br>The total number of diseases/symptoms will exceed the number of included studies because certain diseases/symptoms may fall into more than one category.
**13**	Code of disease	Diseases reported in the study are recorded the particular code according to the International Classification of Diseases 11th Revision (ICD-11) (https://icd.who.int/browse11/l-m/en).
**Part 3**	**Outcomes**	
**14**	TCM-related index	Classified into two categories: YES=including TCM-related index(s), NO=no TCM-related index(s).
**Part 4**	**Funding**	
**15**	Funding reported	Classified into two categories: YES= the study was funded, NO= there was no funding for this study.

**16**	Role of funding	Classified into two categories: YES=role(s) of funding(s) is reported, NO=role(s) of finding(s) is not reported.

Supplementary file 3: Standard operating procedures (SOP) for quality assessment

1.1 SOP for quality assessment of CHMFs protocols

No.	Section/topic	Extension items	Questions for assessment	Definition of Fully reported (scored as 2), Partially reported (scored as 1), Not reported (scored as 0) and Not applicable (NA)	Examples of "fully reported"
1	Title	1a<br>Specify the patient population in terms of 1) a WM-defined disease, 2) a WM-defined disease with a specific TCM pattern, or 3) a TCM pattern.	Q1. Whether the WM-defined diseases or TCM patterns was accurately and specifically reported in the title?	① "Fully Reported" was considered if the title of the study contained a clear description of the WM-defined disease, or TCM pattern, or the names of the WM-defined disease and TCM pattern; if the study investigated two or more TCM patterns while the title contained a generalized description such as "Treatment Based On Pattern Differentiation", it could also be considered as a full report.<br>② "Partially Reported" was considered if generalized descriptions for WM-defined diseases and TCM patterns was reported in title.<br>③ "Not Reported" was considered if the title does not refer to an explicit or generalized WM-defined disease, or TCM patterns, or the names of the WM-defined disease and TCM patterns.	① Rationale and design of the RESTORE trial: A multicenter, randomized, double-blinded, parallel-group, placebo-controlled trial to evaluate the effect of Shenfu injection on myocardial injury in STEMI patients after PCI [268].<br>② Efficacy and safety of the Chinese herbal formula Hewei Jiangni recipe for NERD with cold-heat complex syndrome: study protocol for a double-blinded randomized controlled trial [316].
		1b<br>Specify the intervention, in terms of 1) CHMF, 2) acupuncture, 3)	Q2. Whether the specific intervention was reported in the title?	① "Fully Reported" was considered if the title reported the accurate, transparent and detailed information of specific CHMFs.<br>② "Partially Reported" was considered if it was not able to identify the title whether the	① Rationale and design of the RESTORE trial: A multicenter, randomized, double-blinded, parallel-group, placebo-controlled trial to evaluate the effect of Shenfu

		moxibustion, or 4) other TCM therapy(s).		intervention was a CHMFs or a single herb.<br>③ “Not Reported” was considered if the title did not refer to the specified intervention.	injection on myocardial injury in STEMI patients after PCI [268].<br>② Efficacy and safety of the Chinese herbal formula Hewei Jiangni recipe for NERD with cold-heat complex syndrome: study protocol for a double-blinded randomized controlled trial [316].
2	Background and Rationale	6a.1<br>Provide the background and rationale of the research question with TCM theory.	Q3. Whether the rationale of TCM about CHMFs intervention for the target disease or/and pattern was reported in the background/introduction?	① “Fully Reported” was considered if the background and rationale of the research question with TCM theory were provided in the background and introduction.<br>② “Partially Reported” was considered if only generalized descriptions such as "based on TCM theories" were used to report the theoretical basis in selecting the CHMFs for treating the the target disease or/and pattern in the background and introduction.<br>③ “Not Reported” was considered if the relationship between CHMFs and the target disease or/and pattern were not reported.	① According to CM theory, these herbs are believed to treat Shen and marrow deficiencies by tonifying Shen, invigorating yang, enriching essence and nourishing blood [271].<br>② According to the theory of Chinese medicine, both Qi and blood are basic and vital components of the human body and can maintain activities of living. Qi refers to the energy flow of the body (or a vitality of the body), which maintains blood circulation, warms the body, and fights against disease. In Traditional Chinese Medicine (TCM), blood deficiency is considered a patho- logical state of blood dysfunction and organ dystrophy, which is often caused by spleen and stomach deficiency, haematopoiesis, and blood stasis. Qi and blood supplement each other and support vigour. Qi and blood deficiency are the main pathogenic factors involved in CRF [282].

		6a.2<br>Describe the rationale of the utilized TCM interventions with references.	Q4. Whether the rationale of CHMFs intervention was reported in the background/introduction?	① "Fully Reported" was considered if the rationale of the utillized CHMFs intervention, including the principles, rationale, and prescription analysis of the formula, as well as the applicable data on efficacy, safety and pharmacology, was reported with references.<br>② "Partially Reported" was considered if the rationale of the utillized CHMFs intervention was reported but with no references; or there were references, but only biomedical evidence or Chinese medical theory was reported.<br>③ "Not Reported" was considered if rationale and references of the utillized CHMFs intervention were not reported.	①Tongguan capsules was consisted of radix astragali, radix salviae miltiorrhizae, hirudo and borneolum. Radix astragali had been widely used in CM to treat patients with cardiovascular disease (CVD). It was able to prevent lipid peroxidation by improving antioxidant enzyme activity scavenging free radicals, improving vasorelaxation, and hindering the process of pathological vascular remodeling. Radix salviae miltiorrhizae, also known as Danshen in Chinese, had been widely used to treat CVD in China and other Asia countries. Tanshinone IIA was an important component of Radix salviae miltiorrhizae. Hirudo had been renewed interest in the use of medicinal Leeches, especially for reducing blood coagulation, relieving venous pressure from pooling blood, and treating congestive complications after plastic and reconstructive surgery.Borneolum might facilitate the delivery of other effective components and enhance their effects in combinatorial herbal formulas. A multicenter trial demonstrated that tanshinone IIA inhibited the inflammation reaction and stabilized plaque of patients with CHD subsequently resulted in improved clinical symptoms. Recent experimental research had

					indicated that tanshinone IIA in Tongguan Capsules might reduce myocardial necrosis area and reverse early left ventricular remodeling in rats with acute myocardial infarction (AMI). Importantly, Tongguan Capsules had been shown to exert potent antiproliferative effects on vascular smooth muscle cells, which might be specifically useful in the occurrence of restenosis related primarily to neointimal hyperplasia. Based on the supportive experimental and clinical evidence, to further address the potential benefit of Tongguan Capsules therapy after stent implantation, we prospectively studied a consecutive series of patients undergoing percutaneous stent deployment to determine if Tongguan Capsules therapy reduced restenosis rates, subsequently improve cardiac function and quality of life leading to the reduction of major cardiovascular events finally [269].<br>② Longmu Tang is composed of Os Draconis (Long Gu; 30 g/126 g), Concha Ostreae (Calcined Mu Li; 30 g/126 g), Forsythia suspensa (Lian Qiao; 15 g/126 g), Massa Medi- cata Fermentata (Toasted Shen Qu; 15 g/126 g), Poria cocos (Fu Ling Pi; 30 g/126 g), and Radixet Rhizonma Ephedrae (Ma Huang Gen; 6 g/126 g) (Table 1). Studies have revealed the following. (1)

					Os Draconis and Con- cha Ostreae, the key components of Longmu Tang, exert sedation-like and antipruritic effects in the treatment of AD. In addition, organic calcium in Os Draconis and Concha Ostreae can alleviate allergic symptoms. (2) Forsythia suspensa inhibits Staphylococcus aureus on the skin surface of patients with AD; and (3) Poria cocos bark extract has potential as an oral immune sup- pressor for the treatment of AD through the generation of regulatory T cells. In a previous trial, we found that the Longmu Tang granule could inhibit skin lesions and attenuate atopic itch through suppression of inflam- mation in AD [331].
		6b<br>Describe the rationale and principle(s) for selecting comparators corresponding to certain interventions (i.e. CHMFs, acupuncture, moxibustion or other TCM interventions), considering 1) comparable with tested intervention; 2) success of blinding.	Q5. Whether the rationale and principle for selecting comparators corresponding to CHMFs intervention were reported?	① "Fully Reported" was considered if the rationale and principle for selecting comparators corresponding to CHMFs intervention were reported with details.<br>② "Partially Reported" was considered if the type of control was briefly reported but without the rationale and basis for selecting comparators.<br>③ "Not Reported" was considered if the rationale and principle for selecting comparators corresponding to CHMFs intervention were not reported.	1. A computer-generated randomization schedule was prepared prior to recruitment by a statistician not otherwise involved in the trial, using stata 11 to implement a random number sequence according to simple randomization with a 1:1 allocation ratio. Group assignments were concealed in opaque sequentially numbered envelopes according to the randomization schedule. A print copy of the randomization schedule was stored in a sealed envelope under at research office. With a similar rate of recruitment over time into the two intervention groups, either randomization

					sequence would be expected to produce balanced intervention groups. The study group should be given routine medical treatment for SCAD (including but not limited to anti-platelet aggregation (such as aspirin, once daily, 0.1g each time), lipid-regulating (such as atorvastatin, once daily, 20mg each time) and other drugs), and on this basis, add STDP orally, three times a day, 70 mg each time; By contrast, the control group should be given routine medical treatment for SCAD (including but not limited to anti-platelet aggregation, lipid-regulating and other drugs) [278].<br>② The randomization succession will be created with the PROC PLAN strategy explanations of SAS programming 9.3 bundle by the analyst specialists of Zhejiang Chinese Clinical College, and will be hidden and scattered utilizing obscure envelopes. During the time spent treating and analyzing the data, these spe- cialists will not be involved. By phone, the clinical investigators will apply for a new included patient’s randomization number. The doctors and patients engaged in this RCT won’t likely be rendered blind because there is no usage of herbal medicine in the control group.The randomization strategy will be used to divide the partici- pants into

					one of the 2 groups.<br>The treatment group: conventional western medicine after embryo transplantation combined with Jian-Pi-An-Tai formula. Jian-Pi-An-Tai formula is composed of codonopsis pilosula, astragalus membranaceus, fried atractylodes macrocephala, donkey hide gelatin, mulberry parasitic, dodder seed, raw reh-mannia root, cooked rehmannia root, ramie root, scutellaria baicalensis, paeony root, wolfberry fruit, eclipta alba, perilla frutescens stem, licorice root, and dipsacus, which provided by Preparation Center of the Hangzhou TCM Hospital Affiliated to Zhejiang Chinese Medical University. The patients are planned to take Jian-Pi-An-Tai formula for 5 weeks. The control group: conventional western medicine after embryo transplantation.
3	Objectives	7<br>State the objectives or hypotheses regarding the specific TCM intervention for 1) a WM-defined disease, 2) a WM-defined disease with a specific TCM pattern or 3) a TCM pattern.	Q6. Whether the objectives or hypotheses regarding CHMFs intervention for a WM-defined disease or/and TCM pattern was reported?	① “Fully Reported” was considered if the objectives or hypotheses regarding CHMFs intervention for a WM-defined disease or/and TCM pattern was reported.<br>② “Partially Reported” was considered if only generalized description for a WM-defined disease or/and TCM pattern in the objectives or hypotheses was report.<br>③ “Not Reported” was considered if the objectives or hypotheses regarding CHMFs	① The primary objective is to evaluate whether the ad- ministration of intravenous Shenfu injection, as compared to placebo, could reduce infarct size assessed by CMR in patients with acute anterior STEMI after PPCI.The secondary objective is to evaluate the effects of Shenfu injection as compared with placebo on enzymatic infarct size, microvascular obstruction, intramyocardial hemorrhage, myocardial perfusion, cardiac

				intervention for a WM-defined disease or/and TCM pattern was not reported.	function, and major adverse cardiovascular and cerebrovascular events (MACCE) after PPCI for STEMI [268].<br>② We prospectively studied 400 patients undergoing coronary stent deployment at Guangdong Provincial Hospital of Chinese Medicine. Patient demographic, medical, and procedural information were recorded in our computerized cardiovascular database using a format modeled study protocol after the coronary artery surgery. Patients were followed up for 6 months to determine clinical restenosis (defined as target lesion revascularization by percutaneous coronary intervention or coronary artery bypass grafting), myocardial infarction, and death. The angiographic end point of this trial was to evaluate the efficacy of Tongguan Capsules in reducing clinical restenosis frequency in patients with coronary stent deployment after 6 months after the coronary artery surgery [269].
4	Eligibility Criteria	10a<br>State whether participants with a specific TCM pattern will be recruited, in terms of 1) diagnostic criteria, and 2) inclusion and exclusion	Q7. If participants with a specific TCM pattern would be recruited, whether the TCM diagnostic criteria, inclusion and exclusion criteria and reference were reported?	① "Fully Reported" was considered if the TCM diagnostic criteria, inclusion and exclusion criteria and reference of the recruited participants with a specific TCM pattern were reported.<br>② "Partially Reported" was considered if the TCM diagnostic criteria, inclusion and	① Diagnostic criteria: all participants should meet the diagnostic criteria of NAFLD and TCM pattern differentiation criteria of spleen-yang deficiency. Diagnostic criteria of NAFLD will be based on the American Association for the Study of Liver Diseases (AASLD) in 2017 [12]. To be

		criteria. All criteria utilized should be universally recognized, or reference(s) where detailed explanations can be found should be given.		exclusion criteria and reference of the recruited participants with a specific TCM pattern were not completely reported.<br>③ "Not Reported" was considered if the TCM diagnostic criteria, inclusion and exclusion criteria and reference of the recruited participants with a specific TCM pattern were not reported.<br>④ "Not Applicable" was considered if the participants with no specific TCM pattern were recruited.	specific, the following four criteria should all be met: (a) imaging (ultrasound, computed tomography, or magnetic reson- ance imaging) or histological evidence of hepatic steato- sis; (b) no significant alcohol consumption, defined as > 21 standard drinks per week in men and > 14 standard drinks per week in women; (c) exclusion of other reasons inducing hepatic steatosis, including but not limited to hepatitis C, specific drugs induced, parenteral nutrition, and severe malnutrition; and (d) no coexist- ence of other chronic liver disease, including but not limited to hemochromatosis, autoimmune liver disease, chronic viral hepatitis, alpha-1 antitrypsin deficiency, hepatolenticular degeneration, and drug-induced liver disease. The TCM pattern differentiation criteria of spleen- yang deficiency will refer to the expert consensus of spleen deficiency by China Association of Traditional Chinese Medicine in 2017, previous systematic review, and the clinical guidelines for the new Chinese medicine [29–31]. Ten symptoms and signs will be assessed by continuous 100-point scale, a higher score means a more severe degree. Every symptom or sign possesses with a certain weight. The scoring will be

					calculated through multiplying the rating score by the weight. Spleen-yang deficiency pattern will be defined as total score of 10 di- mensions ≥20. The detailed rating scale is shown in Table Inclusion criteria Participants who meet all of the following criteria will be included: (a) aged 18–80 years, men or women; (b) con- firmed diagnosis of NAFLD; (c) confirmed diagnosis of spleen-yang deficiency pattern; and (d) voluntary informed consent and agreement to participate in every visit, examination, and treatment according to the protocol.Exclusion criteria: Participants who meet any of the following criteria will be excluded: (a) combination with other specific liver diseases which would induce fatty liver, including but not limited to alcoholic liver disease, chronic hepatitis C, autoimmune liver disease, and hepatocellular degener- ation; (b) fatty liver induced by drugs (e.g. tamoxifen, ethylamine iodifurone, valproate, methotrexate, glucocorticoid), total parenteral nutrition, inflammatory bowel disease, hypothyroidism, Cushing syndrome, abe- talipoproteinemia, and other syndromes related to insulin-resistance (e.g. lipid atrophic diabetes, Mauriac syndrome); (c) combination with

					serious primary diseases and mental diseases, including but not limited to cardiovascular and cerebrovascular diseases, hepatic diseases, renal diseases, hematologic diseases, cancers, and schizophrenia; (d) combination with diabetes or currently receiving anti-diabetic medicine treatment; (e) currently receiving treatments for NAFLD (including Chinese herbal decoction, Chinese patent medicine, and chemical agents); (f) antibiotics administration in the last month; (g) allergy to compositions of experimental agents or possessing an allergic constitution; (h)pregnancy and lactating women, and women who are likely to be pregnant but refuse to keep predefined contraception measures during the study; (i) participa- tion in other clinical trials in the last 3 months or cur- rently joining other trials; (j) mental or legal disability; (k) cannot obey medical advice for therapeutic lifestyle modifications; and/or (l) suspicious of drug abuse or possessing other forbidden criteria [304].<br>② Eligibility criteria: Eligible patients will be those who fulfill all of the following inclusion criteria and who do not have any of the listed exclusion criteria. Diagnostic criteria (1) Basis of diagnosis of

					CHD (diagnosis of any of the following)/The diagnostic criteria for CHD will be 1 history of myocardial infarction (MI); 2 coronary angiography or computed tomography coronary angiography confirmation of stenosis greater than 50%; and 3 noninvasive imaging stress test diagnostic of coronary artery disease (CAD) (e.g., positive treadmill exercise test (male only), nuclear perfusion scan). Patients will be considered to be diagnosed with CHD if they meet at least one of these criteria [4, 5, 15]. (2) The angina pectoris severity classification diagnostic criteria were formulated by following the "ACC/ AHA /ACP-ASIM Chronic Stable Angina Pectoris Management Guidelines". Canadian Cardiovascular Society (CCS) Angina Pectoris Severity Classification [16]. The standardization of TCM According to the Expert Consensus for Diagnosis and Treatment of Coronary Heart Disease with Stable Angina Pectoris (2018) [17], doctors choose medications based on standardized western medications. The technical guidelines for clinical research of TCM drugs and natural drugs used for angina and coronary artery disease (2011) describe the main symptom as chest pain (chest tightness) [18]. The secondary symptoms

					are sighing and bloating. Tongue characteristic is a purplish tongue and pulse manifestation is a string or astringent pulse. A patient must have the main symptom and one of the secondary symptoms, in accordance with the tongue and pulse conditions, to be diagnosed with Qi stagnation and blood stasis syndrome [19]. Inclusion criteria (1) Age 18 to 80 years;(2) Diagnosed with CHD;(3) CCS classification of angina grades I–III;(4) Patients with angina pectoris twice or more per week;(5) TCM diagnosis of Qi stagnation and blood stasis syndrome; and(6) Submitted informed consent. Exclusion criteria (1) Myocardial infarction in the past 3 months and moderate to severe heart failure, severe cardiopulmonary insufficiency;(2) Percutaneous coronary intervention or coronary artery bypass grafting or implantation of a pacemaker in the past 3 months;(3) Uncontrolled hypertension with systolic blood pressure (SBP) ≥ 180 mmHg or diastolic blood pressure (DBP) ≥ 100 mmHg, or severe arrhythmia;(4) Chest pain caused by other diseases, such as psychosis, severe neurosis, hyperthyroidism, biliary heart syndrome, gastroesophageal reflux, and aortic dissection;(5) Alanine

					transaminase and/or aspartate transaminase values two times higher than the upper reference limit value. Renal insufficiency (defined as serum creatinine value one and a half times higher than the upper reference limit value). Other severe primary diseases, such as hematopoietic system disease and malignant tumor;(6) Allergy to the ingredients of the study drug; and (7) Participation in other clinical trials in the past 1 month[315].
		10b<br>Descriptions of the roles, qualifications and other relevant experience of the researchers (e.g., participant screeners, care providers, outcome assessors, data analysts) in TCM research are recommended.	Q8. Whether the roles, qualifications and other relevant experience of researchers were reported?	① "Fully Reported" was considered if the roles, qualifications and other relevant experience of researchers was completely reported.<br>② "Partially Reported" was considered if only researchers were briefly described but without details of the roles, qualifications and other relevant experience.<br>③ "Not Reported" was considered if the roles, qualifications and other relevant experience of any of researchers were not reported.	① Two experienced gastroenterologists will screen the patients according to the inclusion and exclusion separately. Patients assessed by 2 doctors meeting the criteria of the trial will be included in the trial. In addition, the recruitment advertisements for this trial will be in the form of posters and webpages in the 8 research units for public recruitment. It mainly includes a brief introduction of the drugs, treatment methods, and the rights and interests of the patients in this study. For those who refuse to participate, we will record their basic information and the reasons for refusing to participate. The trial started in January 2017 and will end in December 2020 [275].<br>② The test medication (OKT) and placebo will be made to appear identical and labeled with the randomization code

					number. Independent pharmacists or research as- sistants will administer the trial medications by match- ing the randomization code numbers to the patients. Clinical trial staff (investigator, research assistants, pharmacist, clinical research coordinator [CRC], clinical research associate [CRA]) and the participants will be blinded to the treatment allocation during the trial. After the end of the trial, the blind will be released for analysis of the results. However, in severe medical emergencies such as serious adverse events (SAEs) during the clinical trial, unblinding may be considered according to the standard operating procedures (SOPs) [300].
		10c<br>Descriptions of the qualification and relevant experience of study center(s) involved in a TCM trial are recommended.	Q9. Whether the qualification and relevant experience of study center(s) involved in a TCM trial were described?	① “Fully Reported” was considered if the qualification and relevant experience of study center(s) involved in a TCM trial were completely described.<br>② “Partially Reported” was considered if the qualification or relevant experience of study center(s) involved in a TCM trial were briefly described.<br>③ “Not Reported” was considered if the qualification and relevant experience of study center(s) involved in a TCM trial were not described.	① Subjects will be recruited from the inpatients or outpa- tients in the Endocrinology Department of Shenzhen TCM Hospital as well as local communities through recruitment advertisements or online promotion. The applicants and main members of the department have been well trained, with rich experience in clinical and experimental research. The Endocrinology Department of Shenzhen TCM Hospital has undertaken multi-stage clinical and basic research projects, and advanced experi- mental equipment as well

					as abundant outpatients and inpatients, which jointly ensure the source of patients, technical force and research conditions required by this project. Recruitment was carried out on June 1, 2022, and is supposed to be completed on January 31, 2024.<br>② (This entry has not been fully reported in any study, and examples of partial reporting are applicable for reference) The scientific design, organization and conduct of the study were supervised by a Steering Committee. The participating centers of this study comprised four institutions across Guangdong Province, including Guangdong Provincial Hospital of Chinese Medicine, Ersha Island Hospital, Hospital of Guangzhou Higher Education Mega Center, and Guangzhou Mercy Hospital. Each participating center had a nominated research assistant responsible for local data collection [270].
5	Interventions	11a.1<br>Interventions for the experimental group(s) with sufficient detail to allow replication.	Q10. Whether the name of the CHMFs and each medical substance were reported?	① "Fully Reported" was considered if all the name of the CHMFs and each medical substance were completely reported.<br>② "Partially Reported" was considered if the name of the CHMFs was not clear and each medical substance were not completely reported.<br>③ "Not Reported" was considered if the	①The test drugs are LCO9 granules and LC09 mimetic agent, provided by pharmacy department of the China-Japan Friend- ship Hospital. The composition of specific Chinese herbal remedies is summarized in Table 1 [274].

<table>
<tr><td></td><td></td><td></td><td></td><td>name of the CHMFs and each medical substance were not reported.
④ “Not Applicable” was considered if TCM intervention was patent proprietary CHMFs.</td><td><table><tr><td colspan="3">Table 1
Constituents of LC09 formula.</td></tr><tr><th>Herb Chinese name</th><th>Botanical Latin name</th><th>Ratio</th></tr><tr><td>Huangqi</td><td>Astragali Radix</td><td>2</td></tr><tr><td>Danggui</td><td>Angelicae Sinensis Radix</td><td>2</td></tr><tr><td>Laoguancao</td><td>Erodii Herba Geranii Herba</td><td>2</td></tr><tr><td>Zicao</td><td>Amebiae Radix</td><td>2</td></tr><tr><td>Honghua</td><td>Carthami Flos</td><td>1</td></tr></table>② QZWTG is a TCM preparation and is commercially developed to relieve epigastric distress and pain. QZWTG is composed of 6 kinds of Chinese herbals (Table 1): Radix Bupleuri (Chaihu), Rhizoma Corydalis (Yanhusuo (baked)), Fructus Aurantii (Zhike), Nutgrass Galingale Rhizome (Xiangfu (baked)), Radix Paeoniae Alba (Baishao), and Radix Glycyrrhizae Preparata (Zhigancao) [275].</td></tr>
<tr><td></td><td></td><td></td><td>Q11. Whether the source of the CHMFs was reported?</td><td>① “Fully Reported” was considered if the source of the CHMFs was completely reported.
② “Partially Reported” was considered if more than one CHMFs were used in the study, the source of all the CHMFs were not completely reported.
③ “Not Reported” was considered if the source of the CHMFs was not reported.
④ “Not Applicable” was considered if TCM intervention was patent proprietary CHMFs.</td><td>① Huoxue-Jangtang Decoction (HJD) is the precom- pounded prescription order of the Endocrinology Department of Shenzhen Traditional Chinese Medicine Hospital (Shenzhen TCM Hospital) [288].
② Danggui Shaoyao Powder (DGSY, composed of Danggui, Shaoyao, Ful- ing, Baizhu, Zexie, and Chuanxiong) was obtained from the Synopsis Golden Chamber [290].</td></tr>
<tr><td></td><td></td><td></td><td>Q12. Whether the origin and processing method of the CHMFs were reported?</td><td>① “Fully Reported” was considered if the origin and processing method of the CHMFs</td><td><table><tr><td colspan="6">Table 1 Components of TXYF granule</td></tr><tr><th>Chinese name</th><th>Botanical name</th><th>Latin name</th><th>Part used</th><th>Source</th><th>Dosage (g)</th></tr><tr><td>Baizhu</td><td>Atratylodes macrocephala macrocephala Koidz.</td><td>Rhizoma Atractylodis Macrocephalae</td><td>Rhizome</td><td>Zhejiang</td><td>18</td></tr><tr><td>Baishao</td><td>Paeonia lactiflora Pall.</td><td>Paeoniae Radix Alba</td><td>Root</td><td>Sichuan</td><td>12</td></tr><tr><td>Chenpi</td><td>Citrus reticulata Blanco</td><td>Citri Reticulatae Pericarpium</td><td>Matured pericarp</td><td>Guangdong</td><td>9</td></tr><tr><td>Fangfeng</td><td>Saposhnikovia divaricata (Turez.) Schischk.</td><td>Saposhnikoviae Radix</td><td>Root</td><td>Neimeng</td><td>6</td></tr></table></td></tr>
</table>

				were reported. 1)The origin of at least one drug was reported, which could be abbreviated. 2)The processing method of at least one drug was reported, which could be abbreviated.<br>② "Partially Reported" was considered if the origin or processing method of the CHMFs was reported; more than one CHMFs were used in the study, the source of all the CHMFs were not completely reported.<br>③ "Not Reported" was considered if the origin and processing method of the CHMFs were not reported.<br>④ "Not Applicable" was considered if TCM intervention was patent proprietary CHMFs.	①TXYF granule and placebo are uniformly manufactured by Guangdong YiFang pharmaceutical co., LTD under the guidance of Good Manufacturing Practices (GMP), which have the identical appearance and nearly similar taste. TXYF granule is made up of Rhizoma Atractylodis Macrocephalae, Paeoniae Radix Alba, Citri Reticulatae Pericarpium, and Saposhnikoviae Radix (see Table 1). These four crude herbs are weighed in proportion of the prescription, subsequently washed, and crushed, and the thickness of the slices is 0.3–1.5 cm. Rhizoma Atractylo- dis Macrocephalae and Paeoniae Radix Alba are fired while Citri Reticulatae Pericarpium and Saposhnikoviae Radix are raw for use. According to the optimized im- plementation scheme for decocting, extracting, and con- centrating of the crude herbs, the ointments are prepared with a relative density of 1.07 to 1.09 g/ml, and then the ointments are screened to get granulated oint- ments. Finally, after spray drying and crushing, the ranules are packed in sealed opaque packages. Placebo consisting of maltodextrin, dextrin, experimental medi- cation extract (< 5%), kudingcha extract, and pigment is also prepared granules [309].

					② The Guilu Xian/placebo will be given to the patients at 5 g three times daily from the third day of their menstru- ation period before IVF for 40days (provided by the preparation room of Affiliated Hospital of Shandong University of Traditional Chinese Medicine, and the ex- traction procedure will be based on the standard of preparation room SOP). Drug composition: tortoise glue, turtle glue, antler glue, Chinese wolfberry, American gin- seng, Cornus officinalis, Hawthorn seed, jujube [322].
			Q13. Whether the dosage form, production method and administration route of the CHMFs were reported?	① "Fully Reported" was considered if the dosage form, production method and administration route of the CHMFs were clearly and completely reported.<br>② "Partially Reported" was considered if the dosage form or production method or administration route of the CHMFs was reported; more than one CHMFs were used in the study, the dosage form, production method and administration route of all the CHMFs were not completely reported.<br>③ "Not Reported" was considered if the dosage form, production method and administration route of the CHMFs were not reported.<br>④ "Not Applicable" was considered if TCM intervention was patent proprietary CHMFs.	① The granules will be packaged into small single-dose sachets, each weighing 16 g. All the drugs will be uniformly packaged and identified with the same labels. Administration method: 32 g of the granules will be dissolved in 60°C to 70°C water and diluted to 1000 mL in thermostatic foot bath tub with herbal liquid temperature 35°C to 38°C. This is then used to soak the hands and feet for 20 minutes twice a day. After washing and immersion intervention, patients in both groups are to apply vaseline moisturizer on their hands and feet for local moisturizing. Treatment duration lasts for 7 consecutive days [274].<br>② Test drugs including FBD and placebo

<table>
<tr><td></td><td></td><td></td><td></td><td></td><td>were prepared by the Department of Pharmacy of the Affiliated Hospital of Chengdu University of Traditional Chinese Medicine. The composition of the FBD is as follows: Raw Rhubarb (Sheng dahuang) 20g, Coptidis Rhizoma (Huang lian) 15g, Fructus Forsythia (Lian qiao) 15 g, Aluminum potassium sulfate (Ku fan) 10 g, Pseudobulbus Cremastrae Seu Pleiones (Shan cigu) 10g. All herbs are mixed, soaked, cooked, and made into a single-dose pouch of uniform specifications by the pharmacy department, each bag is about 150 mL. Placebos are made from starch without active ingredients and added a variety of food colors [276].</td></tr>
<tr><td></td><td></td><td></td><td>Q14. Whether the dosage of CHMFs and each medical substance was reported?</td><td>① “Fully Reported” was considered if the dosage of CHMFs and each medical substance was completely reported.
② “Partially Reported” was considered if the dosage of the CHMFs and each medical substance was reported; more than one CHMFs were used in the study, the dosage of all the CHMFs and each medical substance were not completely reported.
③ “Not Reported” was considered if the dosage of the CHMFs and each medical substance were not reported.
④ “Not Applicable” was considered if TCM intervention was patent proprietary CHMFs.</td><td>Table 1
Constituents of LC09 formula.<table><tr><th>Herb Chinese name</th><th>Botanical Latin name</th><th>Ratio</th></tr><tr><td>Huangqi</td><td>Astragali Radix</td><td>2</td></tr><tr><td>Danggui</td><td>Angelicae Sinensis Radix</td><td>2</td></tr><tr><td>Laoguancao</td><td>Erodii Herba Geranii Herba</td><td>2</td></tr><tr><td>Zicao</td><td>Amebiae Radix</td><td>2</td></tr><tr><td>Honghua</td><td>Carthami Flos</td><td>1</td></tr></table>① Administration method: 32 g of the granules will be dissolved in 60°C to 70°C water and diluted to 1000 mL in thermostatic foot bath tub with herbal liquid temperature 35°C to 38°C [274].
② The participants assigned to intervention group will take DGSY granules (8.4 g/pack × 2 packs, bid) and those</td></tr>
</table>

<table>
<tr><td></td><td></td><td></td><td></td><td></td><td>assigned to placebo group will take placebo granules (2 packs, bid) for 24 weeks. According to the initial pre- scription dose and clinical treatment practice of NAFLD, the daily raw drug dose for adults in the DGSY group was Angelica sinensis. (Danggui) 9 g, Angelica sinensis. (Shaoyao) 20 g, Wolfiporia cocos. (Fuling) 12 g, Atracty- lodes macrocephala. (Baizhu) 12 g, Alismatis Rhizoma. (Zexie) 20 g; and Ligusticum wallichii Franch. (Chuanx- iong) 12 g [290].</td></tr>
<tr><td></td><td></td><td></td><td>Q15. Whether the reference(s) to dosage of CHMFs was reported?</td><td>① “Fully Reported” was considered if the reference(s) to dosage of CHMFs was reported.
② “Partially Reported” was considered if a brief basis for dosage of CHMFs was reported, with no references indicated; more than one CHMFs were used in the study, the reference(s) to dosage of CHMFs was not completely reported.
③ “Not Reported” was considered if the reference(s) to dosage of the CHMFs was not reported.
④ “Not Applicable” was considered if TCM intervention was patent proprietary CHMFs.</td><td>(This entry has not been fully reported in any study, and examples of partial reporting are applicable for reference)
①
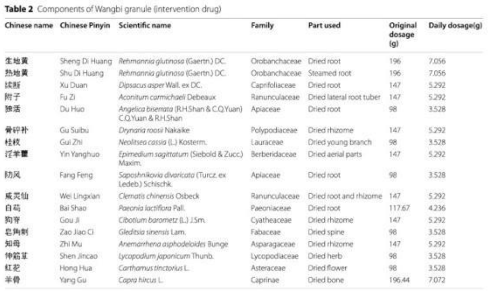
Table 2 Components of Wangbi granule (intervention drug)
<table>
<tr><th>Chinese name</th><th>Chinese Pinyin</th><th>Scientific name</th><th>Family</th><th>Part used</th><th>Original dosage (g)</th><th>Daily dosage(g)</th></tr>
<tr><td>生地黄</td><td>Sheng Di Huang</td><td>Rehmannia glutinosa (Gaertn.) DC.</td><td>Orobanchaceae</td><td>Dried root</td><td>196</td><td>7.056</td></tr>
<tr><td>熟地黄</td><td>Shu Di Huang</td><td>Rehmannia glutinosa (Gaertn.) DC.</td><td>Orobanchaceae</td><td>Steamed root</td><td>196</td><td>7.056</td></tr>
<tr><td>续断</td><td>Xu Duan</td><td>Dipsacus asper Wall. ex DC.</td><td>Caprifoliaceae</td><td>Dried root</td><td>147</td><td>5.292</td></tr>
<tr><td>附子</td><td>Fu Zi</td><td>Aconitum carmichaeli Debeaux</td><td>Ranunculaceae</td><td>Dried lateral root tuber</td><td>147</td><td>5.292</td></tr>
<tr><td>独活</td><td>Du Huo</td><td>Angelica biserrata (R.H.Shan & C.Q.Yuan) C.Q.Yuan & R.H.Shan</td><td>Apiaceae</td><td>Dried root</td><td>98</td><td>3.528</td></tr>
<tr><td>骨碎补</td><td>Gu Suibu</td><td>Drynaria roosii Nakaike</td><td>Polypodiaceae</td><td>Dried rhizome</td><td>147</td><td>5.292</td></tr>
<tr><td>桂枝</td><td>Gui Zhi</td><td>Neolitsea cassia (L.) Kosterm.</td><td>Lauraceae</td><td>Dried young branch</td><td>98</td><td>3.528</td></tr>
<tr><td>淫羊藿</td><td>Yin Yanghuo</td><td>Epimedium sagittatum (Siebold & Zucc.) Maxim.</td><td>Berberidaceae</td><td>Dried aerial parts</td><td>147</td><td>5.292</td></tr>
<tr><td>防风</td><td>Fang Feng</td><td>Saposhnikovia divaricata (Turcz. ex Ledeb.) Schischk.</td><td>Apiaceae</td><td>Dried root</td><td>98</td><td>3.528</td></tr>
<tr><td>威灵仙</td><td>Wei Lingxian</td><td>Clematis chinensis Osbeck</td><td>Ranunculaceae</td><td>Dried root and rhizome</td><td>147</td><td>5.292</td></tr>
<tr><td>白芍</td><td>Bai Shao</td><td>Paeonia lactiflora Pall.</td><td>Paeoniaceae</td><td>Dried root</td><td>117.67</td><td>4.236</td></tr>
<tr><td>狗脊</td><td>Gou Ji</td><td>Cibotium barometz (L.) J.Sm.</td><td>Cyatheaceae</td><td>Dried rhizome</td><td>147</td><td>5.292</td></tr>
<tr><td>皂角刺</td><td>Zao Jiao Ci</td><td>Gleditsia sinensis Lam.</td><td>Fabaceae</td><td>Dried spine</td><td>98</td><td>3.528</td></tr>
<tr><td>知母</td><td>Zhi Mu</td><td>Anemarrhena asphodeloides Bunge</td><td>Asparagaceae</td><td>Dried rhizome</td><td>147</td><td>5.292</td></tr>
<tr><td>伸筋草</td><td>Shen Jincao</td><td>Lycopodium japonicum Thunb.</td><td>Lycopodiaceae</td><td>Dried herb</td><td>98</td><td>3.528</td></tr>
<tr><td>红花</td><td>Hong Hua</td><td>Carthamus tinctorius L.</td><td>Asteraceae</td><td>Dried flower</td><td>98</td><td>3.528</td></tr>
<tr><td>羊骨</td><td>Yang Gu</td><td>Capra hircus L.</td><td>Caprinae</td><td>Dried bone</td><td>196.44</td><td>7.072</td></tr>
</table>
[296].
② In the Longmu Tang granule group, participants will be allocated to receive the Longmu Tang granule (9.8 g/ sachet) orally with 50 mL of warm water twice a day for 8 weeks. Active granules, each containing 9.8</td></tr>
</table>

					g of Longmu Tang, are equivalent to 126 g of crude medicine. The pla- cebo granule is composed of 5% crude Longmu Tang and 95% starch with a similar appearance and smell to the Longmu Tang granule [331].
			Q16. Whether the administration route of the CHMFs was reported?	① "Fully Reported" was considered if the administration route of the CHMFs , including when, where, by whom or by what organization, and how it was carried out, whether samples were retained, was reported.<br>② "Partially Reported" was considered if the administration route of the CHMFs was reported without when, where, by whom or by what organization, or how it was carried out or whether samples were retained.<br>③ "Not Reported" was considered if the administration route of the CHMFs was not reported.<br>④ "Not Applicable" was considered if TCM intervention was patent proprietary CHMFs.	(This entry has not been fully reported in any study, and examples of partial reporting are applicable for reference)<br>① Administration method: 32 g of the granules will be dissolved in 60°C to 70°C water and diluted to 1000 mL in thermostatic foot bath tub with herbal liquid temperature 35°C to 38°C [274].<br>② The trial group will be given QZWTG (2.5 G each time) and Mosapride citrate tablet placebo (5mg each time). The control group will be given QZWTG placebo (2.5 G each time) and Mosapride citrate tablet (5mg each time). All of them will be given 3 times a day, 30 minutes before breakfast, lunch, and dinner. The QZWTG placebo consists of starch without any active ingredient is produced by the same manufacturer as QZWTG. It is a dextrin that matches as much as possible the appearance and taste of QZWTG. The drug instructions for QZWTG and placebo are completely consistent. Both the drug and the corresponding placebo had the same outer packaging,

					color, shape, and taste so that neither the participants nor the researchers could identify which intervention the participants were receiving. The packaging of the drug will be returned to the investigator at the end of the treatment [275].
			Q17. Whether the quality control of each ingredient and the whole formulae and the safety assessment of the whole formulae were conducted?	① "Fully Reported" was considered if the quality control of each ingredient was conducted. Quality control methods for each ingredient include any quantitative and/or qualitative testing methods, and when, where, how and by whom or by what organization they were performed, where the raw data and samples were kept, and whether they were applicable; the safety assessment of the whole formulae including heavy metal and toxic element tests, pesticide residue tests, microbial limit tests, acute/chronic toxicity tests; as well as when, where, how and by whom or by what organization, where the raw data and samples were kept, and whether they were accessible.<br>② "Partially Reported" was considered if the quality control of each ingredient and the whole formulae or the safety assessment of the whole formulae was reported.<br>③ "Not Reported" was considered if the quality control of each ingredient and the whole formulae and the safety assessment of the whole formulae was not reported.	①All investigational medicinal product (IMP) including the Chinese medicine JCM-16021 granules and placebo are produced by the Purapharm (Nanning) Pharmaceuti- cal Co. Ltd. The entire manufacturing process will be in strict compliance with the standards of Good Manufac- tory Practice (GMP). The production process of the Chi- nese medicine JCM-16021 granules and placebo granules will be compliant with the quality specification standards, respectively. According to the Chinese Pharmacopoeia (2015), all qualified IMP will be delivered to the site of clinical study and stored within a range of 25±2 °C and relative humidity range of 60±10% [24]. The investiga- tors are responsible for ensuring adequate accountability of all used and unused IMP. For better guaranteeing the safety and quality of the Chinese medicine JCM-16021 granules, accelerated stability tests, heavy mental and toxic elements, pesticides residues, and microbial limit will be conducted [292].

				④ “Not Applicable” was considered if TCM intervention was patent proprietary CHMFs.	② Both the XBYRT and placebo granules will be manu- factured by Kinhong Pte Ltd., Singapore, a Good Manu- facturing Practices-certified manufacturer. A certificate of analysis containing the results on component identifi- cation, moisture content and the absence of heavy metal and microbial contamination will be applicable for each batch of XBYRT/placebo granules manufactured [308].
			Q18. For protocol with individualized CHMFs, whether it reported how, when, and by whom the CHMFs was modified?	① “Fully Reported” was considered if the protocol was with individualized CHMFs, the details of individualized CHMFs, including whether it reported how, when, and by whom the CHMFs was modified, should be reported.<br>② “Partially Reported” was considered if the protocol was with individualized CHMFs, the details of individualized CHMFs, including whether it reported how, when, and by whom the CHMFs was modified, were incompletely reported.<br>③ “Not Reported” was considered if the protocol was with individualized CHMFs, no details of individualized CHMFs was reported.<br>④ “Not Applicable” was considered if a CHMFs or a patent proprietary CHMFs was conducted to use in the study.	①<br>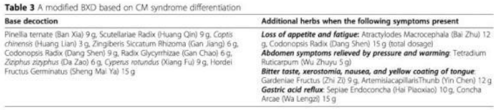<br>**Table 3** A modified BXD based on CM syndrome differentiation<br>**Base decoction** — Pinellia ternate (Ban Xia) 9 g, Scutellariae Radix (Huang Qin) 9 g, *Coptis chinensis* (Huang Lian) 3 g, Zingiberis Siccatum Rhizoma (Gan Jiang) 6 g, Codonopsis Radix (Dang Shen) 9 g, Radix Glycyrrhizae (Gan Chao) 6 g, *Ziziphus zizyphus* (Da Zao) 6 g, *Cyperus rotundus* (Xiang Fu) 9 g, Hordei Fructus Germinatus (Sheng Mai Ya) 15 g<br>**Additional herbs when the following symptoms present** — ***Loss of appetite and fatigue:*** Atractylodes Macrocephala (Bai Zhu) 12 g, Codonopsis Radix (Dang Shen) 15 g (total dosage); ***Abdomen symptoms relieved by pressure and warming:*** Tetradium Ruticarpum (Wu Zhuyu 5 g); ***Bitter taste, xerostomia, nausea, and yellow coating of tongue:*** Gardeniae Fructus (Zhi Zi) 9 g, ArtemisiacapillarisThunb (Yin Chen) 12 g; ***Gastric acid reflux:*** Sepiae Endoconcha (Hai Piaoxiao) 10 g, Concha Arcae (Wa Lengzi) 15 g<br>[312]<br>② The individualized TCM are as follows: blood deficiency, plus white peony, Angelica; yin deficiency and fire wang signs clearly, plus Anemarrhena asphodeloides, Cortex Phellodendri; qi deficiency, plus Radix ginseng, Schisandra; blood stasis, plus Panax notoginseng, Salvia Miltiorrhiza; yang deficiency, plus Eucommia, Herba Epimedii; turbid-phlegm, plus Rhizoma Pinelliae, Poria cocos; water-dampness, plus Grifola umbellata, Alisma; qi stagnation, plus Fructus aurantii, Dried tangerine peel. The therapeutic formula will be adjusted based on the patient’s clinical performance each treatment cycle

					[399].
			Q19. For protocol with patent proprietary CHMFs, whether the name and dosage of formulae were reported?	① "Fully Reported" was considered if the protocol was with patent proprietary CHMFs, the name and dosage of formulae were completely reported.<br>② "Partially Reported" was considered if the protocol was with patent proprietary CHMFs, name or dosage of formulae were reported; more than one patent proprietary CHMFs were used in the study, the name and dosage of all formulaes were not completely reported.<br>③ "Not Reported" was considered if the protocol was with patent proprietary CHMFs, the name and dosage of formulae were not reported.<br>④ "Not Applicable" was considered if TCM intervention was not patent proprietary CHMFs.	① The experimental medi- cine of FFEJS and placebo are both manufactured specifi- cally by Dong-E-E-Jiao Co., Ltd (Production batch number: 1910063). The participants in the FFEJS group will be administered with FFEJS, an oral liquid preparation, the same original marketed patent medicine (20 ml per bottle), 3 times per day for a total of 6 weeks. Each bottle of FFEJS contains donkey-hide gelation (Colla Corii Asini, 870 mg), red ginseng (Radix Ginseng Rubra, 435mg), prepared rehmannia root (Radix Rehmanniae Preparata, 3043mg), dang shen (Radix Codonopsis, 3043mg), and hawthorn fruit (Fructus Crataegi, 1304mg) [280].<br>② In traditional Chinese medicine, the decoction is pre- pared by boiling in water for hours. However, DKP (Lot No. Z20059003, Shanxi Guangyuyuan Medicine Co., Ltd., China) preparation will adopt the water-honeyed pill protocol according to the Chinese Pharmacopoeia (ChP) 2015 Edition standard [15] and ChP 2005 Edition. The "DKP water-honeyed pill" standard [16] is approved by the China Food and Drug Administration (CFDA). Each TCM bottle is filled with 7 g DKP [284].

			Q20. For protocol with patent proprietary CHMFs, whether the efficacy of formulae was reported?	① "Fully Reported" was considered if the protocol was with patent proprietary CHMFs, the efficacy of formulae was reported, e.g. manufacturer's name and manufacturing lot number; or references and instructions to the efficacy of the formulae.<br>② "Partially Reported" was considered if the protocol was with patent proprietary CHMFs, the efficacy of formulae was reported without references; more than one patent proprietary CHMFs were used in the study, the efficacy of all formulaes were not completely reported.<br>③ "Not Reported" was considered if the protocol was with patent proprietary CHMFs, the name and dosage of formulae were not reported.<br>④ "Not Applicable" was considered if TCM intervention was not patent proprietary CHMFs.	① Individuals in the experimental arm (174 cases) took 0.6 g of GLJC (0.3 g/capsule, provided by Guang Yu Yuan Co., Ltd., China, batch No. 103180601) once a day and 19.2 mg of Gingko biloba extract mimetic 3 times a day. Individuals in the active comparator arm (174 cases) took 0.6 g of Guilingji mimetic once a day and 19.2 mg of Gingko biloba extract in tablet form (19.2 mg/tablet, provided by Yangzi River Pharmaceutical Group Co., batch No. 10010321) 3 times a day [271].<br>② The Baidu Jieduan granules will be administered orally two times a day for 14days. The Baidu Jieduan granules are manufactured by Beijing Tcmages Pharmaceutical Co., Ltd. (number: Jing 20180032) [314].
			Q21. For protocol with patent proprietary CHMFs, whether the safety assessment and quality control of formulae were reported?	① "Fully Reported" was considered if the protocol was with patent proprietary CHMFs, the safety assessment and quality control of formulae were reported, e.g. manufacturer's name and manufacturing lot number; or the text or annex contains standards and methods of quality control and safety monitoring.<br>② "Partially Reported" was considered if there is no manufacturer's name and manufacturing lot number, only a general	① Individuals in the experimental arm (174 cases) took 0.6 g of GLJC (0.3 g/capsule, provided by Guang Yu Yuan Co., Ltd., China, batch No. 103180601) once a day and 19.2 mg of Gingko biloba extract mimetic 3 times a day. Individuals in the active comparator arm (174 cases) took 0.6 g of Guilingji mimetic once a day and 19.2 mg of Gingko biloba extract in tablet form (19.2 mg/tablet, provided by Yangzi River

				description of safety monitoring and quality control without specific methods or reference standards; the name or manufacturer or manufacturing lot number of patent proprietary CHMFs was reported; more than one patent proprietary CHMFs were used in the study, the safety assessment and quality control of all formulaes were not completely reported.<br>③ "Not Reported" was considered if the protocol was with patent proprietary CHMFs, the safety assessment and quality control were not reported.<br>④ "Not Applicable" was considered if TCM intervention was not patent proprietary CHMFs.	Pharmaceutical Group Co., batch No. 10010321) 3 times a day [271].<br>② The Baidu Jieduan granules will be administered orally two times a day for 14days. The Baidu Jieduan granules are manufactured by Beijing Tcmages Pharmaceutical Co., Ltd. (number: Jing 20180032) [314].

			Q22. For protocol with patent proprietary CHMFs, whether the details of the formulae were illustrated?	① "Fully Reported" was considered if the protocol was with patent proprietary CHMFs, the details of the formulae should be illustrated, including: product name (i.e. trade name), manufacturer, manufacturing lot number, date of manufacture and expiry date, name and content of excipients.<br>② "Partially Reported" was considered if the details of the formulae, including product name (i.e. trade name), manufacturer, manufacturing lot number, date of manufacture and expiry date, name and content of excipients, was partially illustrated; more than one patent proprietary CHMFs were used in the study, the the details of all formulaes were not completely reported.<br>③ "Not Reported" was considered if the protocol was with patent proprietary CHMFs, the details of the formulae were not reported.<br>④ "Not Applicable" was considered if TCM intervention was not patent proprietary CHMFs.	（This entry has not been fully reported in any study, and examples of partial reporting are applicable for reference）<br>① MYN is produced and packed by Sichuan Purity Pharmaceutical Co., Ltd. To ensure the stability of the drug, the same batch of drugs will be used in this study (drug batch number: 051–210917). MYN is cur- rently under patent review. We have uploaded all of the specific components, dosage, and other information of MYN to the official website of China National Intellectual Property Administration, which is open for access and can be found on the patent search and service system page of the official website of China National Intellectual Property Adminis- tration [28] via publication no.: CN109908209A. MYN will be administered intranasally and two pumps/nostril will be sprayed twice daily. The drug will not be administered once the patient meets the clinical control standard for two weeks. The shortest treatment time will be not less than four weeks, and the longest treatment time will be not more than three months [353].<br>② All the eligible participants were randomly divided into an experimental arm and an active comparator arm. Individuals in the experimental arm (174 cases) took

					0.6 g of GLJC (0.3 g/capsule, provided by Guang Yu Yuan Co., Ltd., China, batch No. 103180601) once a day and 19.2 mg of Gingko biloba extract mimetic 3 times a day. Individuals in the active comparator arm (174 cases) took 0.6 g of Guilingji mimetic once a day and 19.2 mg of Gingko biloba extract in tablet form (19.2 mg/tablet, provided by Yangzi River Pharmaceutical Group Co., batch No. 10010321) 3 times a day. The intervention period included two sessions over 24 weeks. To ensure consistency of participating centers, all the practitioners will attend professional training courses together before the trial [271].
			Q23. Whether the patent proprietary CHMFs utilized in the protocol was identical to the public applicable reference?	① “Fully Reported” was considered if the patent proprietary CHMFs utilized in the protocol was identical to the public applicable reference.<br>② “Partially Reported” was considered if it is reported the patent proprietary CHMFs could treat the target disease or evidence type without references or did not state that the indication corresponded to the study methodology； more than one patent	This entry has not yet been fully reported in a study, and examples are not yet applicable.

				proprietary CHMFs were used in the study, all of which were not identical to the public applicable reference.<br>③ "Not Reported" was considered if the patent proprietary CHMFs utilized in the protocol was not identical to the public applicable reference.<br>④ "Not Applicable" was considered if TCM intervention was not patent proprietary CHMFs.	
		11a.2<br>Describe interventions for the control group(s) with sufficient detail to allow replication.	Q24. For protocol with placebo control, whether the name and dosage of each ingredient, the similarity of placebo with the intervention (e.g., color, smell, taste, appearance, packaging), the quality control and safety assessment of placebo,the administration route, regimen, and dosage and the production information of placebo,including when, where, how, and by whom the placebo was produced were reported?	① "Fully Reported" was considered if protocol was with placebo control, the name and dosage of each ingredient, the similarity of placebo with the intervention (e.g., color, smell, taste, appearance, packaging), the quality control and safety assessment of placebo,the administration route, regimen, and dosage and the production information of placebo,including when, where, how, and by whom the placebo was produced were reported.<br>② "Partially Reported" was considered if protocol was with placebo control, the details of placebo were incompletely reported.<br>③ "Not Reported" was considered if protocol was with placebo control, the details of placebo were not reported.<br>④ "Not Applicable" was considered if the protocol was not with placebo control.	① Placebo treatment: placebo granules contained 5% DGSY granules and 95% other ingredients (contains 98.77% maltodextrin, 0.75% caramel pigment, 0.15% lemon yellow pigment, 0.03% sunset yellow pigment, and 0.30% bitters). The pellets were masked with the same packaging and a similar taste as DGSY. Both DGSY and placebo will be manufactured and packaged by Tianjiang Pharmaceutical Co. Ltd., Jiangyin City, Jiangsu Province, China [290].<br>② The control group receives placebo treatment, which mainly consists of starch without any effective ingredient. The placebo is provided by the same manufacturer as YXS. It is a dextrin that matches as much as possible in appearance, shape, color, size, package, and the taste with YXS table with no side effects on

					human health. All these medicines are consistent with the Chinese Medicine Standards of the State Food and Drug Administration [277].
			Q25. For protocol with active control, if a CHMFs was used, refer to the recommendations of 11a.1A; if a chemical agent will be used, whether the name,administration route, dosage and regime were reported?	① "Fully Reported" was considered if protocol was with active control and a CHMFs was used as active control, refer to the recommendations of 11a.1A; if a chemical agent was used, the name,administration route, dosage and regime were completely reported.<br>② "Partially Reported" was considered if protocol was with active control, the details of active control were incompletely reported.<br>③ "Not Reported" was considered if protocol was with active control, the details of active control were not reported.<br>④ "Not Applicable" was considered if the protocol was not with active control.	（This entry has not been fully reported in any study, and examples of partial reporting are applicable for reference）<br>① The trial group will be given QZWTG (2.5 G each time) and Mosapride citrate tablet placebo (5mg each time). The control group will be given QZWTG placebo (2.5 G each time) and Mosapride citrate tablet (5mg each time) [275].<br>② The participants will be categorized into two groups receiving either standard Western medicine alone ac- cording to the Protocol for Diagnosis and Treatment of Novel Coronavirus Pneumonia (7th edition) or the com- bination of Baidu Jieduan granules two times a day for 14 days plus standard care [314].
		11d.2 Descriptions of other interventions that will be administrated to experimental and/or control groups are recommended (e.g., rescue interventions),	Q26. Whether the details of other interventions administered to experimental and/or control groups were reported?	① "Fully Reported" was considered if the details of other interventions administered to experimental and/or control groups were completely reported.<br>② "Partially Reported" was considered if the name of other interventions was reported but without part of details.<br>③ "Not Reported" was considered if details of other interventions administered to	①The participants will be categorized into two groups receiving either standard Western medicine alone ac- cording to the Protocol for Diagnosis and Treatment of Novel Coronavirus Pneumonia (7th edition) or the com- bination of Baidu Jieduan granules two times a day for 14 days plus standard care. Routine care includes early fluid resuscitation, antimicrobial

		with enough details to allow replication.		experimental and/or control groups were not reported.<br>④ “Not Applicable” was considered if the protocol was not with other interventions administered to experimental and/or control groups.	anticoagulants, nutri- tional support and other treatments. Other TCM therap- ies, including TCM injections and other oral herbal medicines, will be prohibited [314].<br>② Simultaneously, all patients after intervention would receive standard anti-ischemic therapy according to patients' conditions, such as aspirin, clopidogrel, angiotensin converting enzyme inhibitors or beta- blockers, calcium channel blockers and nitrate esters irrespective of the initial randomization assignment. All procedures as well as medicines prescription would be under responsibility of physicians according to the clinical guidelines. The date of any medical therapy changes and the reasons would be documented in the case record form (CRF) [269].
6	Outcomes	12a<br>Provide the rationale of TCM-related indexes as outcomes (e.g. the change of degree and scope of symptoms and signs related to pattern differentiation).	Q27. Whether the rationale of TCM-related indexes as outcomes were reported?	① “Fully Reported” was considered if the rationale and references of TCM-related indexes as outcomes were completely reported.<br>② “Partially Reported” was considered if only a brief description of the rationale of TCM-related indexes as outcomes was reported but without references.<br>③ “Not Reported” was considered if the protocol was not with TCM-related indexes as outcomes.	①TCM syndrome scores will be assessed according to the Guidance Principle of Clinical Research on New Drug of Traditional Chinese Medicine (2002 edition) [27], which is widely used in the evaluation of TCM syndrome in China. The efficacy is classified into clinical recovery, markedly effective, effective, and non-effective: clinical recovery, TCM clinical symptoms disappeared or symp- tom score reduction ≥ 95%; markedly effective, symptom score reduction ≥ 70% and < 95%; effective,

					symptom score reduction ≥ 30% and < 70%; and non-effective, no significant improvement in clinical symptoms of TCM or symptom score reduction < 30%. The calculation for- mula is based on the nimodipine method: Efficacy index = [(score before treatment – score after treatment) / score before treatment] × 100%. The evaluation will be performed at baseline, day 6, day 21, and day 42 [282].<br>② Five typical symptoms for LSSD are assessed to evalu- ate the changes of TCM Pattern, involving: (i) abdominal distension, (ii) borborygmus and flatus, (iii) distension and fullness in chest and hypochondrium, (iv) frequent sighing, poor appetite, and (v) mental depression or irri- tability. Investigators will grade the TCM pattern scale (None=0, Mild=1, Moderate=2, Severe=3) as shown in Additional file 3: Table S2. Efficacy assessment stand- ards of Syndrome of Chinese medicine are as follows: Clinical remission: clinical symptoms and signs disap- pear or basically disappear, total scoring declining ≥ 95%; Excellence: clinical symptoms and signs are significantly improved, total scoring declining ≥70%; Effective: clini- cal symptoms and signs are improved, total scoring declining ≥ 30%; Invalid: clinical

					symptoms and signs are without obvious improvement or even with exacerbation, total scoring declining <30%. The effective rate on TCM pattern will be evaluated with following calculation for- mula (nimodipine method): [(total score of prior treat- ment – total score of post treatment)/total score of prior treatment] × 100% [23] [292].
		12b<br>Provide the details of the TCM-related outcomes assessment, including i) the measuring methods and standard (e.g. frequency, severity rating scale of symptoms and signs, verified pattern questionnaire, time points for assessment and corresponding rationale), ii) assessor qualification (e.g. relevant assessment experience, years in clinical practice), iii) methods used to enhance the quality of assessment (e.g. multiple repeated observation, training of	Q28. Whether the details of the TCM-related outcomes assessment were described?	① "Fully Reported" was considered if he details of the TCM-related outcomes assessment were completely described, including methods and criteria for measurement (e.g., frequency of occurrence, severity scores of symptoms and signs, validated questionnaires, selection of time points for assessment, and related rationale); qualifications of the assessor (e.g., relevant assessment experience, years of experience); methods to improve the quality of the assessment (e.g., repeated observations, training of the assessor); and relevant references.<br>② "Partially Reported" was considered if only the name or references of the TCM-related outcomes assessment was reported.<br>③ "Not Reported" was considered if the protocol was not with TCM-related indexes as outcomes.	①TCM syndrome scores will be assessed according to the Guidance Principle of Clinical Research on New Drug of Traditional Chinese Medicine (2002 edition) [27], which is widely used in the evaluation of TCM syndrome in China. The efficacy is classified into clinical recovery, markedly effective, effective, and non-effective: clinical recovery, TCM clinical symptoms disappeared or symp- tom score reduction ≥ 95%; markedly effective, symptom score reduction ≥ 70% and < 95%; effective, symptom score reduction ≥ 30% and < 70%; and non-effective, no significant improvement in clinical symptoms of TCM or symptom score reduction < 30%. The calculation for- mula is based on the nimodipine method: Efficacy index = [(score before treatment – score after treatment) / score before treatment] × 100%. The evaluation will be performed at baseline, day 6, day 21, and day 42 [282].

		assessors), and iv) related reference(s).			② Five typical symptoms for LSSD are assessed to evalu- ate the changes of TCM Pattern, involving: (i) abdominal distension, (ii) borborygmus and flatus, (iii) distension and fullness in chest and hypochondrium, (iv) frequent sighing, poor appetite, and (v) mental depression or irri- tability. Investigators will grade the TCM pattern scale (None=0, Mild=1, Moderate=2, Severe=3) as shown in Additional file 3: Table S2. Efficacy assessment stand- ards of Syndrome of Chinese medicine are as follows: Clinical remission: clinical symptoms and signs disap- pear or basically disappear, total scoring declining ≥ 95%; Excellence: clinical symptoms and signs are significantly improved, total scoring declining ≥70%; Effective: clini- cal symptoms and signs are improved, total scoring declining ≥ 30%; Invalid: clinical symptoms and signs are without obvious improvement or even with exacerbation, total scoring declining <30%. The effective rate on TCM pattern will be evaluated with following calculation for- mula (nimodipine method): [(total score of prior treat- ment – total score of post treatment)/total score of prior treatment] × 100% [23] [292].
7	Data Collection Methods	18a	Q29 If protocol targets TCM pattern, or a WM- defined disease	① “Fully Reported” was considered if protocol targets TCM pattern, or a WM-	（This entry has not been fully reported in any study, and examples of partial reporting

		When trial targeting TCM pattern, or a WM-defined disease with a specific TCM pattern, baseline data about TCM pattern should be provided.	with a specific TCM pattern, whether the baseline data about TCM pattern was provided?	defined disease with a specific TCM pattern, the baseline data about TCM pattern was provided.<br>② “Partially Reported” was considered if protocol targets TCM pattern, or a WM-defined disease with a specific TCM pattern, the baseline data collection or statistics that the evidence type would be considered was mentioned, but without details on how to implement.<br>③ “Not Reported” was considered if protocol targets TCM pattern, or a WM- defined disease with a specific TCM pattern, the baseline data about TCM pattern was not provided.<br>④ “Not Applicable” was considered if protocol targets WM-defined disease not TCM pattern, or a WM- defined disease with a specific TCM pattern.	are applicable for reference）<br>①<br>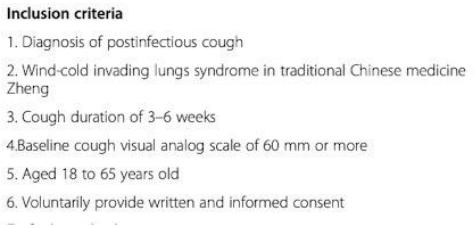<br>**Inclusion criteria**<br>1. Diagnosis of postinfectious cough<br>2. Wind-cold invading lungs syndrome in traditional Chinese medicine Zheng<br>3. Cough duration of 3–6 weeks<br>4.Baseline cough visual analog scale of 60 mm or more<br>5. Aged 18 to 65 years old<br>6. Voluntarily provide written and informed consent<br>[306]<br>② The inclusion criteria of TCM refers to the diagnosis of cold-heat complex syndrome in the “consensus on Inte- grated traditional Chinese and Western Medicine diagnosis and treatment of gastroesophageal reflux Disease” issued by the Digestive Professional Committee of the Chinese Soci- ety of Integrated traditional Chinese and Western Medicine in 2017 (Table 2) [316].

1.2 SOP for quality assessment of acupuncture protocols

No.	Section/topic	Extension items	Questions for assessment	Definition of Fully reported (scored as 2), Partially reported (scored as 1), Not reported (scored as 0) and Not applicable (NA)	Examples of fully reported
1	Title	1a<br>Specify the patient population in terms of 1) a WM-defined disease, 2) a WM-defined disease with a specific TCM pattern, or 3) a TCM pattern.	Q1. Whether the diseases or patterns was accurately and specifically reported in the title?	① "Fully Reported" was considered if the title of the study contained a clear description of the WM-defined disease, or TCM pattern, or the names of the WM-defined disease and TCM pattern; if the study investigated two or more TCM patterns while the title containd a generalized description such as "Treatment Based On Pattern Differentiation", it could also be considered as a full report.<br>② "Partially Reported" was considered if generalized descriptions for WM-defined diseases and TCM patterns was reported in title<br>③ "Not Reported" was considered if the title does not refer to an explicit or generalized WM-defined disease, or TCM patterns, or the names of the WM-defined disease and TCM patterns.	① Acupuncture for hot flashes in hormone receptor- positive breast cancer, a coordinated multinational study: Rationale and design of the study protocol[1].<br>② Acupuncture versus sham acupuncture in the treatment of diabetic distal symmetric polyneuropathy (DSPN): Study protocol for a randomized controlled trial[2].
		1b<br>Specify the intervention, in terms of 1) CHMF, 2) acupuncture, 3) moxibustion, or 4) other TCM therapy(s).	Q2. Whether the specific intervention was reported in the title?	① "Fully Reported" was considered if the title explicitly denotes the specified intervention as acupuncture.<br>② "Partially Reported" was considered if the title did not provide sufficient information to determine whether the intervention was of acupuncture.<br>③ "Not Reported" was considered if the title does not refer to the specified intervention.	① Acupuncture has been reported to reduce hot flashes in patients with breast cancer undergoing adjuvant hormonal therapy[1].<br>② Acupuncture versus sham acupuncture in the treatment of diabetic distal symmetric polyneuropathy (DSPN): Study protocol for a randomized controlled trial[2].

2	Background and Rationale	6a.1<br>Provide the background and rationale of the research question with TCM theory.	Q3. Whether the rationale of TCM about acupuncture intervention for diseases or TCM patterns was reported in the background/introduction?	① "Fully Reported" was considered if the detailed TCM theoretical principles of acupuncture intervention of the disease or pattern was reported in the background and introduction.<br>② "Partially Reported" was considered if the theoretical basis of TCM for the treatment of the disease or pattern by acupuncture was reported only with a generalized description such as "based on Chinese medicine theory" in the background and introduction.<br>③ "Not Reported" was considered if the relationship between acupuncture and the target disease or/and pattern was not reported.	① Based on the meridian theory, acupuncture therapy could promote flow of qi and blood circulation and regulate the balance of Yin and Yang, which has been used in the treatment of many diseases and clinical syndromes[35].<br>② According to the traditional Chinese acupuncture theory, meridians and viscera are closely related in physiological function, mutually affected in pathological changes and treatment. The Heart Meridian connects with the heart directly, and the Lung Meridian indirectly associates with the heart. Both of these two meridians can be used for cardiovascular diseases in clinical practice[52].
		6a.2<br>Describe the rationale of the utilized TCM interventions with references.	Q4. Whether the rationale of acupuncture intervention was reported in the background/introduction?	① "Fully Reported" was considered if the rationale of the utillized acupuncture intervention, including the principles, rationale, and prescription analysis of the formula, as well as the available data on efficacy, safety and pharmacology, was reported with references.<br>② "Partially Reported" was considered if the rationale of the utillized acupuncture intervention was reported but with no references; or there were references, but only biomedical evidence or Chinese medical theory was reported.<br>③ "Partially Reported" was considered if	① Previous studies that investigated diabetic neuropathy suggested that acupuncture can alleviate pain and improve quality of life .Clinical studies and animal models investigating the physiological mechanisms of acupuncture have demonstrated an association between acupuncture treatment and an increase in blood circulation, which is beneficial for improving patient's neuropathic symptoms . One study has investigated the application of

				rationale and references of the utillized acupuncture intervention were not reported.	acupuncture for a variety of subjective symptoms associated with diabetic peripheral neuropathy[2].<br>② A large number of studies have demonstrated the efficacy and advantages of acupuncture therapy in alleviating chronic pain, insomnia, anxiety, and depression. Acupuncture exhibits a rapid pain-relieving effect for the chronic soft tissue diseases, which has been confirmed by research findings on its anti-inflammatory and analgesic effects and improvement of microcirculation[35].
		6b<br>Describe the rationale and principle(s) for selecting comparators corresponding to certain interventions (i.e. CHMFs, acupuncture, moxibustion or other TCM interventions), considering 1) comparable with tested intervention; 2) success of blinding.	Q5. Whether the he rationale and principle(s) for selecting comparators corresponding to acupuncture intervention was reported?	① "Fully Reported" was considered if the type of control, as well as the rationale and basis for the control was reported in the full text.<br>② "Partially Reported" was considered if the type of control was simply described (e.g., sham acupuncture control, blank control, waiting list, etc., without mentioning the rationale and basis for setting up the control.<br>③ "Not Reported" was considered if the type of control and the rationale and basis for the control was not reported in the full text.	① Eligible participants were randomly allocated to the TEAS and sham-TEAS groups at a ratio of 1:1 with a random number table.The electrical stimulator was placed in an opaque container. An envelope containing grouping information was given to an acupuncturist, and the acupuncturist's sole responsibility was to place the electrodes and turn the stimulator on and off. All operations were completed by the same operational team, and the surgeons, anesthesiologists, and follow-up observers were all blinded to the grouping of patients[5].

					② Randomization will be performed by an independent<br>third party, Linkermed Technology Co. Ltd. (Beijing,<br>China). Randomization sequence will be generated by<br>computers in varying block sizes and stratified by centers, and central randomization system will be adopted to conduct the process. Once the patient is confirmed eligible to be enrolled, the study coordinator in charge of the randomization will log into the central<br>randomization system to obtain the allocation information. Patients will be randomly allocated into the EA group, SA group, or solifenacin treatment group at a ratio of 1:1:1[17].
3	Objectives	7<br>State the objectives or hypotheses regarding the specific TCM intervention for 1) a WM-defined disease, 2) a WM-defined disease with a specific TCM pattern or 3) a TCM pattern.	Q6. Whether the objectives or hypotheses regarding acupuncture intervention was reported?	① "Fully Reported" was considered if the purpose or hypothesis of the acupuncture study was explicitly reported as targeting a Western disease, or a TCM pattern, or the names of a Western disease and a TCM pattern; if the study targeted two or more TCM patterns, a generalized description such as "TCM diagnosis and treatment" appeared in the title.<br>② "Partially Reported" was considered if the purpose of the study generalized descriptions of	① The overall goal of this research is to evaluate the impact of acupuncture on the frequency and severity of hot flashes in a multinational population of breast cancer survivors experiencing hot flashes during adjuvant hormonal therapy[2].<br>② Therefore, the aim of this study is to investigate the effects of laser acupuncture on constipation, to offer a better and simpler

				diseases and patterns, such as respiratory diseases, or TCM diagnosis and treatment.<br>③ "Not Reported" was considered if it was not stated that the purpose or hypothesis of the acupuncture study was for a Western medicine disease, or a Chinese medicine evidence type, or a Western medicine disease with a Chinese medicine evidence type.	therapy for treating constipation in advanced cancer patients[7].
4	Eligibility Criteria	10a<br>State whether participants with a specific TCM pattern will be recruited, in terms of 1) diagnostic criteria, and 2) inclusion and exclusion criteria. All criteria utilized should be universally recognized, or reference(s) where detailed explanations can be found should be given.	Q7. If participants with a specific TCM pattern would be recruited, whether the TCM diagnostic criteria, inclusion and exclusion criteria and reference(s) was reported?	① "Fully Reported" was considered if subjects with specific TCM pattern types were recruited, their diagnostic criteria, inclusion and exclusion criteria should be reported in detail. It is essential to utilize recognized diagnostic criteria or provide references that can be consulted for detailed explanations.<br>② "Partially Reported" was considered if it failing to meet any of the TCM diagnostic criteria, inclusion exclusion criteria, or references.。<br>③ "Not Reported" was considered if subjects meeting specific TCM evidence types were recruited; however, the TCM diagnostic criteria, inclusion/exclusion criteria, and references were not provided in the text.<br>④ "Not Applicable " was considered if refraining from recruiting subjects exhibiting a specific TCM evidence pattern.	①<br>TCM diagnostic criteria. According to the expert<br>consensus on diagnosis and treatment of hypertension in TCM(2019 edition),[5] the guiding principles for clinical research of new Chinese medicine in the treatment of hypertension[6] and the criteria for diagnosis and treatment of TCM syndromes (2017edition),[7] the TCM diagnostic criteria for HLY will be formulated. On the basis of the main symptoms, those who have ≥3 secondary symptoms at the same time combined with tongue and pulse conditions, can be diagnosed as HLY. To guarantee the consensus on inquiry method, judgment of syndrome type and record method, the clinical data will be collected by unified questionnaire and unified criteria. It will be

					reviewed by the deputy chief physician and above. If the judgment of syndrome type is inconsistent, 2 chief physicians of TCM willassist in the diagnosis[233].<br>② Inclusion criteria<br>1. Age: 18 to 65 years, both male and female;<br>2. Met the Western diagnostic criteria of essential hypertension. For patients without antihypertensive drugs, SBP: 140 to 179 mmHg and/or DBP: 90 to 109mmHg. For patients with anti-hypertensive drugs, hypertension should be stabilized at a normal high value. The patients who took medicine used≤2 antihypertensive drugs for >2 months.<br>3. Confirmed TCM diagnosis of HLY in hypertension.<br>4. Not receives acupuncture or Chinese herbal medicine treatment in recent 1 month.<br>5. Good compliance with the observation and evaluation of researchers.<br>6. Informed consent is signed by the patient himself or his immediate family members.<br>Exclusion criteria<br>1. Secondary hypertension, such as aldosteronism, pheochromocytoma, Cushing

					syndrome, gestational hypertension, obstructive sleep apnea hypopnea syndrome, and so on.<br>2. Accompanied by other serious cardiovascular and cerebrovascular diseases, nephropathy, retinopathy, peripheral vascular diseases, diabetes, blood diseases, and so on.<br>3. There were skin diseases and severe skin damage near the acupoints.<br>4. Mental disorders, pregnant, and lactating women.<br>5. Participating in other clinical trials[233].<br>Inclusion criteria<br>Patients satisfying the following six conditions will be included:<br>A. Diagnosis of 'apoplexy' according to TCM and cerebral infarction confirmed by computed tomography or magnetic resonance imaging<br>B. Age of 35 to 80 years<br>C. A duration from stroke onset of 2 weeks to 3<br>1. months<br>D. National Institute of Health stroke scale (NIHSS) score of 5–22<br>E. A limb strength score below grade 4 and

					limb dysfunction<br>F. Qi deficiency syndrome and blood stasis syndrome confirmed by the 'Ischemic Ischaemic Stroke TCM Syndrome Diagnostic Scale (ISTCMDS)'<br>Exclusion criteria<br>Patients with any of the following conditions will be excluded:<br>A. Intracerebral haemorrhage or subarachnoid haemorrhage confirmed by computed tomography or magnetic resonance imaging<br>B. Recurrent stroke with a neurofunctional disability that can affect the results of this study<br>C. A history of a brain tumour, traumatic brain injury, haematopathy, etc.<br>D. Other severe diseases that can affect cerebral infarction therapy outcomes and mental impairment<br>E. A pregnant or lactating status<br>F. Participation in another clinical trial for a drug, acupuncture or rehabilitation<br>G. Skin lesions and other skin diseases at acupuncture points[63].
		10b	Q8. Whether the roles, qualifications and other	① "Fully Reported" was considered if the role, qualifications and relevant experience	① The acupuncture treatments in both groups will be performed by the same

		Descriptions of the roles, qualifications and other relevant experience of the researchers (e.g., participant screeners, care providers, outcome assessors, data analysts) in TCM research are recommended.	relevant experience of the researchers was reported?	qualifications of the participating researcher (e.g. subject screeners, healthcare providers, outcome evaluators, data analysts) were reported in the full text.<br>② "Partially Reported" was considered if only a concise portrayal of the researcher is provided, for instance, the practitioner is solely identified as a physician without elaborating on their qualifications and pertinent expertise.<br>③ "Not Reported" was considered if the roles, qualifications, and relevant experience of individuals involved in the study (e.g., subject screeners, healthcare providers, outcome assessors, data analysts) were not reported.	acupuncturist with 10 years of clinical experience throughout the study[8].<br>② The treatment will be provided by licensed acupuncturists holding acupuncture physician certifications in China with at least 3 years of clinical experience[12].
		10c<br>Descriptions of the qualification and relevant experience of study center(s) involved in a TCM trial are recommended.	Q9. Whether the qualification and relevant experience of study center(s) involved in a TCM trial was described?	① "Fully Reported" was considered if the qualification and relevant experience of the research center participating in the TCM trial were reported in the full text.<br>② "Partially Reported" was considered if only the name or qualification of the research center of the TCM trial was simply described. For example, patient recruitment was only described in XX hospital or in a tertiary hospital.<br>③ "Not Reported" was considered if it failure to provide comprehensive reporting on the qualifications and relevant experience of research centers involved in clinical trials of Chinese medicine.	① Participating sites include Dana-Farber Cancer Institute (DFCI), Boston, US; Daegu Catholic University Medical Center (DCUMC), Daegu, Republic of Korea; and Jiangsu Provincial Hospital of Traditional Chinese Medicine (JPHTCM), Nanjing, China[1].<br>② This RCT will be performed at the First Affiliated Hospital of Changchun University of Chinese Medicine, China. It is a comprehensive tertiary level Chinese medicine hospital with medical treatment, scientific research, prevention, and health care. The hospital has a clinical research center for acupuncture and moxibustion[79].

5	Interventions	11a.1<br>Interventions for the experimental group(s) with sufficient detail to allow replication.	Q10. Whether the treatment environment and participant posture was reported?	① "Fully reported" was considered if the treatment environment and participant posture were reported. For the treatment environment, it could be descriptions of temperature, humidity, noise, light, etc. The patient's position/posture during the acupuncture treatment could be prone, sitting, lying sideways, and so on. Statements such as "any position was acceptable as long as the patient was comfortable", "there were no special requirements for position/posture", or other expressions with similar meanings in the article can also be considered "fully reported".<br>② "Partially Reported" was considered if only the treatment environment and participant posture were reported.<br>③ "Not Reported" was considered if neither the treatment environment nor participant posture were not reported.	① The acupuncture treatments in both groups will be performed by the same acupuncturist with 10 years of clinical experience throughout the study, the room temperature will be kept 25°C and other influencing factors are prevented to avoid bias[8].<br>② Before undergoing acupuncture, patients will be asked to lie in the supine position in a quiet environment [46].
			Q11. Whether the number of needle insertions per subject per session (mean and range if possible) and names and locations of acupoints (uni/bilateral) was reported?	① "Fully reported" is considered if there was information related to the number of needle insertions per subject per session (mean and range if possible) and names and locations of acupoints (uni/bilateral) was reported. For the number, the total number of treated points with acupuncture per subject per session, or the mean/range of points' numbers selected across all participants can be described. For names and locations of acupoints (uni/bilateral), the standard nomenclature for acupoints (e.g.	① Acupoints on both sides of the body are as follows: SP10 (Xue Hai), GB34 (Yang Ling Quan), ST36 (Zu San Li), SP6 (San Yin Jiao), ST41 (Jie Xi), and EX-le10<br>(Ba Feng) in the lower extremity. All participants will take a supine position to receive acupuncture therapy. After skin disinfection with 75% alcohol, the acupuncturists will place sterile adhesive pads on bilateral acupoints, and then insert the

				GB21) and/or meridians (e.g., conception vessel), anatomical location ,“The pain site or ashi point was selected for acupuncture treatment”, or other expressions with similar meanings can be described.<br>② “Partially Reported” was considered if only the number of needle insertions per subject per session or names and locations of acupoints (uni/bilateral) was reported.<br>③ “Not Reported” was considered if neither the number of needle insertions per subject per session nor names and locations of acupoints (uni/bilateral) was not reported.	needles into the skin. A disposable needle (size 0.30 × 50 mm) will be used for SP10, GB34, ST36, and SP6. Acupuncture needles will be inserted vertically through the adhesive pads into the skin for a distance of 25 mm. Disposable needles(size 0.30 × 40 mm) will be used for bilateral ST41 and EX-LE10, these acupoints will be punctured for a distance of 15 mm into the skin. Equal manipulations of twirling, lifting, and thrusting will be performed on all needles to evoke the needle sensation of de qi (a composite of sensations including soreness, numbness, distention, heaviness, and other sensations)[2].<br>② After skin disinfection, the double-sided adhesive pad (Φ1 × 15 mm) and opaque plastic base (Φ4 × 15 mm, Φ5 × 10 mm) of the customized Hwato acupuncture device (Product batch number: 200304, Figure 4) will be placed on the skin surface, and a verum needle with a sharp needle tip (Hwato, Suzhou, China 0.3 mm × 25 mm/ 0.3 mm× 40 mm) placed in a plastic catheter (large: Φ4 x 20mm, small: Φ3 x 35mm) and stabbed to the skin along the direction of the catheter. For

					bilateral ST36, ST37, and LI4, it is approximately 20–30 mm, 20–30 mm, and 10–20 mm, respectively[4].
			Q12. Whether the angle and depth of insertion was reported?	① "Fully reported" was considered if the angle and depth of insertion was reported.The depth of insertion should be expressed using the Chinese measurement of the cun; in terms of anatomical depth, for example, of subcutaneous tissue, fascia, muscle or periosteum; or in millimetres. The angle of needle insertion can be described with specific numerical values; if a straight puncture, oblique puncture, or flat puncture was reported, but the specific numerical value was not stated, it can also be regarded as reporting the needle insertion angle.<br>② "Partially Reported" was considered if only the angle or depth of insertion was reported.<br>③ "Not Reported" was considered if neither the angle nor depth of insertion was reported.	① In perpendicular needling group, the needles will be vertically inserted to SP6 bilaterally in a depth of 1 to 1.2 cun and will be manipulated by lifting-thrusting and twirling methods for 30 seconds to achieve proper needling sensation. In transverse needling group, the needles will be inserted to bilateral SP6 transversely 1 to 1.2 cun toward the abdomen without any manipulation to avoid needling sensation[8].<br>② The acupuncture points (ST36, SP6, P6, and LI4) will be punctured with 0.25×40mm stainless steel needles using the one-hand insertion method. The needle will be inserted into the skin surface with a depth of 5 to 20mm depending on the acupoint. Lifting and twisting techniques will be used for these points until the participant can feel the needle sensation (ie, "Deqi," a sensation of soreness, numbness, distention or heaviness around the needle). After the needle is removed, the patient assumes the prone position, and receives acupuncture their back, with needle

					retention for 20minutes. Back-shu points will be punctured with 0.25×25mm stainless steel needles without performing any needling manipulations[9].
			Q13. Whether the response sought from participants (e.g., de qi or muscle twitch response) and the needle stimulation (e.g., manual, electrical) was described?	① "Fully reported" was considered if there were the response sought from participants (e.g., de qi or muscle twitch response) and the needle stimulation (e.g., manual, electrical) was reported. For the needle stimulation included manual or electrical. If electroacupuncture stimulation was used, the brand, manufacturer and frequency of use of the electroacupuncture instrument should be indicated. For participants responses included the expected responses or the actual responses. For example the de qi sensation in traditional Chinese acupuncture, the muscle twitch in trigger point treatment or muscle contraction in electro-acupuncture, or other expressions with similar meanings. It was also considered as participants responses were reported if authors described "No special response was found", or "All participants responded normally".<br>② "Partially or not reported" was considered if only the response sought from participants or the needle stimulation was reported; or only the	① After insertion, acupuncture needles are manually manipulated until the acupuncturist obtains the De Qi sensation (feeling a tugging or grasping sensation from the needle manipulation coupled with soreness, fullness, heaviness or local distension at local needling sites) from at least two points bilaterally on the lower extremity and at least one point bilaterally on the upper extremity[1].<br>② Equal manipulations of twirling, lifting, and thrusting will be performed on all needles to evoke the needle sensation of de qi (a composite of sensations including soreness, numbness, distention, heaviness, and other sensations)[2].

				name of the stimulation technique was reported, such as electrical stimulation, but there was no description of the specific operation, such as current intensity, amplitude, frequency and other details.<br>③ "Not Reported" was considered if neither the response sought from participants nor the needle stimulation was reported.	
			Q14. Whether the needle type (e.g. diameter, length, and manufacturer or material) was described?	① "Fully reported" was considered if there was all information related to the needle type used for acupuncture, including the materials (e.g., stainless steel, gold and silver), diameter, length, product name and manufacturer.<br>② "Partially reported" was considered if only partial information of diameter or length or manufacturer or material was reported.<br>③ "Not reported" was considered if there was no information about needle type.	① after sterilizing the skin on the areas where the needles will be inserted, by using single-use sterile filiform needles of 0.30×40mm (Zhongyan Taihe, Beijing Zhongyan Taihe Medical Instruments center, Beijing, China)[8].<br>② Disposable, sterile needles with a diameter of0.25 mm and a body length of 40 mm(Huatuo, Suzhou, China) will be used[12].
			Q15. Whether the number of acupuncture treatment sessions, the frequency and duration of treatment sessions and the needle retention time was reported?	① "Fully reported" was considered if the information related to the acupuncture treatment sessions, the frequency or duration of acupuncture treatment sessions and the needle retention time was reported.<br>② "Partially reported" was considered if only partial information about the acupuncture treatment sessions, or the frequency or duration of acupuncture treatment sessions, or the needle retention time was reported.<br>③ "Not reported" was considered if there was	① The needles at all points will be retained for 30 min with light lifting, thrusting, and twirling every 10min during each session. Participants will receive 18 treatment sessions given 3 times per week (ideally every other day) for 6 continuous weeks[11].<br>② Patients in the acupuncture group will receive standard pharmacological treatment same as the control group, and receive acupuncture two times a week for 12 weeks for a total of 24 treatment sessions.Patients

				no information about the acupuncture treatment sessions, or the frequency or duration of acupuncture treatment sessions, or the needle retention time was reported.	will receive treatment in supine position for 20 min per session[12].
		11a.2<br>Describe interventions for the control group(s) with sufficient detail to allow replication.	Q16. Whether any special arrangements in pre-treatment, treatment and post-treatment periods corresponding to the experimental intervention was reported if the study protocol was the blank/waitlist control?	① "Fully reported" was considered if the study used the no-treatment or waiting-list controls, and the researchers included any special arrangements in pre-treatment, treatment, and post-treatment periods corresponding to the experimental intervention was reported. For example, examinations in pre-treatment period, unaltered lifestyle and medication in treatment period, and compensatory interventions in post-treatment period.<br>② "Partially reported" was considered if the study used a blank or wait-list control and only partial details of the control were reported.<br>③ "Not Reported" was considered if only the study was mentioned, a blank or wait-list control was selected, but no details of the control were reported.<br>④ "Not applicable" was considered if there was no blank control or a wait-list control.	① The patients in the control group will receive standard pharmacological treatment. Patients with osteoporosis will take Alendronate Sodium Tablets 10mg (once per day, taken with a glass of warm water before breakfast) for 12 weeks. However, no acupuncture treatment was given during the study period. They will be evaluated at each visit.[12]<br>② Patients in the HA group will accept acupuncture treat- ment three times in the first week, and then twice a week in the next two weeks during each cycle of chemother- apy (21 days for 1 cycle) for three cycles, namely a total of 21 times acupuncture sessions; those in the LA group will take once a week for three cycles, namely a total of 9 times acupuncture sessions.[23]
			Q17. Whether the comparability of the sham acupuncture or acupuncture-like control	① "Fully reported" was considered if the comparability of the sham acupuncture or acupuncture-like control was reported and comprehensively provided details as for the	① In the SA group, the selected stimulation points (non-acupoints 1-6) are close to those of the EA group, but not on the meridian route (Table 1, Figure 3). The SA

			and the comprehensive details as for the recommendations of Intervention 11a.1B were reported if the study protocol was sham acupuncture or acupuncture-like control?	recommendations of Intervention 11a.1B.<br>② "Partially reported" was considered if the study used sham acupuncture or acupuncture-like controls and only partial details of the control were reported.<br>③ "Not Reported" was considered if the study mentioned the use of sham acupuncture or acupuncture-like controls, but failed to provide any pertinent details regarding the control.<br>④ "Not applicable" was considered if the study protocol had no sham acupuncture or acupuncture-like control.	group will have a similar acupuncture insertion process as the EA group; however, the SA group will use a sham needle with a blunt needle tip (Hwato, Suzhou, China 0.3 mm × 25 mm/0.3 mm × 40 mm, Figure 4) that cannot pierce the skin. Specifically, no acupuncture manipulation will be performed during acupuncture. Next, non-acupoints 1 will be connected to non-acupoints 2 with EA, similarly with non-acupoints 4 and 5. The frequency (10 Hz) will be set to the same as the EA group, but no current will pass through (current intensity will be 0 mA) because of the prior connection of the blunt needle to the incorrect output socket. Treatment from postoperative days one to three, once per day.[4]<br>② The sham needle consists of a needle with a blunt tip and a circle adhesive pad (Fig. 1). The needle is very similar to the real needle, 0.30 mm wide, and 40 mm long. The adhesive pad is made of the sterile cylindrical polyethylene foam (10 mm in diameter and 5 mm in length) with a double-sided adhesive tape at the bottom. The adhesive pad supports the sham needle on the acu- point

					and ensures the implementation of blinding during the intervention. Thus, the sham needle with a blunt tip only contacts the skin surface but will not penetrate into the skin of the participants during sham acupuncture.[35]
		11d.2<br>Descriptions of other interventions that will be administrated to experimental and/or control groups are recommended (e.g., rescue interventions), with enough details to allow replication.	Q18. Whether the details of other interventions administered to experimental and/or control groups were reported?	① "Fully reported" was considered if the details of other interventions administered to experimental and/or control groups were reported in the method section, such as general treatment, routine care, rescue interventions, exercise therapy, diet adjustment, etc., including the specific method and duration of intervention implementation.<br>② "Partially reported" is considered if there was insufficient information about the details of other interventions administered to experimental and/or control groups.<br>③ "Not Reported" was considered if there was no information about the details of other interventions administered to experimental and/or control groups.<br>④ "Not applicable" was considered if the study was not with other interventions administered to experimental and/or control	① According to the actual situation of all participants in the two groups, we will follow the theory of neuropsych- ology and provide each participant with the same cogni- tive rehabilitation treatment through a combination of artificial training and computer training that will include classification exercises, rule (response) suppression exer- cises, plan analysis exercises, reasoning comprehension exercises, working memory exercises, and comprehen- sive ability exercises.[55]<br>② During the clinical trial period, all participants will be allowed to continue the routine management regimens, existing medications (e.g., those for hypertension, dia- betes, or hyperlipidemia), and medications for maintain- ing and improving their health status. However, they will not be permitted to

				groups.	engage in other treatments (pharma- cological treatments, physical therapy, or CAM therap- ies) to ameliorate their CLBP symptoms. All medical devices will be inspected by the investigators, who will record the results of checkups in the management register.[60]
6	Outcomes	12a<br>Provide the rationale of TCM-related indexes as outcomes (e.g. the change of degree and scope of symptoms and signs related to pattern differentiation).	Q19. Whether the rationale of TCM-related indexes as outcomes were reported?	① "Fully reported" was considered if the rationale and references of TCM-related indexes as outcomes (e.g. the change of degree and scope of symptoms and signs related to pattern differentiation) were reported.<br>② "Partially reported" was considered if the TCM-related outcome indexes were briefly described without any accompanying references.<br>③ "Not Reported" was considered if the rationale and references of TCM-related indexes as outcomes were not reported.	(This entry has not been fully reported in any study, and examples of partial reporting are applicable for reference)<br>① Clinical symptoms of TCM: symptom quantitative score will be used, which is based on "Guidelines for Clinical Research of New Drugs of Traditional Chinese Medicine" [17]. Four levels are used to assess the severity of symptoms, which include no, mild, moderate and severe. a speci63c score will be given to each level (see attached Table 3). Effectiveness assessment: clinical recovery: TCM symptoms disappear, symptom score decreases by ≥95%; remarkable effectiveness: TCM symptoms and signs are improved signi 63 cantly, symptom score decreases by ≥70%; effectiveness: TCM symptoms and signs improve, symptom score decreases by ≥30%; No effectiveness: TCM symptoms and signs are not improved or aggravated, and the symptom score reduces by <30%.[12]<br>② TCM symptoms will be assessed using TCM symptom scores, which are formulated

					according to TCM diagnostic criteria. When scoring, patients only need to an- swer “yes” or “no”; “yes” counts as 1 point, “no” counts as 0 points, and the points are combined to calculate the total score (Table 1). The recurrence rate will also be calculated to evaluate the efficacy of fire needle therapy.[45]
		12b<br>Provide the details of the TCM-related outcomes assessment, including i) the measuring methods and standard (e.g. frequency, severity rating scale of symptoms and signs, verified pattern questionnaire, time points for assessment and corresponding rationale), ii) assessor qualification (e.g. relevant assessment experience, years in clinical practice), iii) methods used to enhance the quality of assessment (e.g. multiple repeated observation, training of assessors), and iv) related reference(s).	Q20. Whether the details of the TCM-related outcomes assessment were described?	① “Fully reported” was considered if the details of the TCM-related outcomes assessment, including i) the measuring methods and standard (e.g. frequency, severity rating scale of symptoms and signs, verified pattern questionnaire, time points for assessment and corresponding rationale), ii) assessor qualification (e.g. relevant assessment experience, years in clinical practice), iii) methods used to enhance the quality of assessment (e.g. multiple repeated observation, training of assessors), and iv) related reference(s) were reported.<br>② “Partially reported” was considered if only names or references of TCM-related outcomes assessment were reported.<br>③ “Not Reported” was considered if the TCM-related outcomes assessment was not reported.	① Clinical symptoms of TCM: symptom quantitative score will be used, which is based on "Guidelines for Clinical Research of New Drugs of Traditional Chinese Medicine" [17]. Four levels are used to assess the severity of symptoms, which include no, mild, moderate and severe. a speci64c score will be given to each level (see attached Table 3). Effectiveness assessment: clinical recovery: TCM symptoms disappear, symptom score decreases by ≥95%; remarkable effectiveness: TCM symptoms and signs are improved signi 64 cantly, symptom score decreases by ≥70%; effectiveness: TCM symptoms and signs improve, symptom score decreases by ≥30%; No effectiveness: TCM symptoms and signs are not improved or aggravated, and the symptom score reduces by <30%.[12]<br>② TCM syndrome evaluation will be

					conducted accord- ing to the 2002 “Guiding Principles for Clinical Research of New Chinese Medicines [17]” based on the degree of joint pain; number of joints involved; joint swelling, ten- derness, flexion, and extension; and other symptoms or signs. Scores will be divided into four levels: none, 0 points; light, 1 point; severe, 2 points; and very severe, 3 points. Four types of curative effects will be considered: (1) recovery: TCM syndrome scores decreased by ≥ 70%;(2) significant effect: TCM syndrome scores decreased by ≥ 50%; (3) effective: TCM syndrome scores decreased by ≥ 20%; and (4) ineffective: TCM syndrome scores de- creased by less than 20%, whereby effectiveness rate = ([points before treatment – points after treatment]/ points before treatment) × 100%.[69]
7	Data Collection Methods	18a<br>When trial targeting TCM pattern, or a WM- defined disease with a specific TCM pattern, baseline data about TCM pattern should be provided.	Q21. If trial targeting TCM pattern, or a WM- defined disease with a specific TCM pattern, whether the baseline data about TCM pattern was provided?	① “Fully reported” was considered if the study was specific to TCM patterns, or disease and TCM patterns, baseline data collection including TCM patterns information was reported, such as baseline characteristics and differences between populations with different patterns. It mainly described whether TCM patterns were considered in the baseline data	This entry has not yet been fully reported in a study, and examples are not yet applicable.

				and statistical analysis.<br>② "Partially reported" was considered if it was only mentioned in the baseline data collection or statistics that the TCM patterns would be considered, but there were no details on how this would be implemented.<br>③ "Not Reported" was considered if the baseline data did not include information regarding the TCM patterns.<br>④ "Not applicable" was considered if the study focuses on the disease rather than the TCM patterns or TCM patterns and disease.	

1.3 SOP for quality assessment of moxibustion protocols

No.	Section/topic	Extension items	Questions for assessment	Definition of Fully reported (scored as 2), Partially reported (scored as 1), Not reported (scored as 0) and Not applicable (NA)	Examples of "fully reported"

1	Title	1a<br>Specify the patient population in terms of 1) a WM-defined disease, 2) a WM-defined disease with a specific TCM pattern, or 3) a TCM pattern.	Q1. Whether the diseases or patterns was accurately and specifically reported in the title?	① "Fully Reported" was considered if the title of the study should provide a precise description of either the Western medicine disease, Chinese medicine pattern, or both; in cases where multiple Chinese medicine patterns are targeted, a comprehensive report can also be indicated by using a more specific title such as "Chinese Medicine Diagnosis and Treatment".<br>② "Partially Reported" was considered if the title encompassed generalized descriptions of respiratory diseases and evidence types, including TCM evidence-based treatment<br>③ "Not Reported" was considered if the title does not refer to an explicit or generalized Western medicine disease, or Chinese medicine evidence type, or the names of the Western medicine disease and Chinese medicine evidence type.	① Efficacy and safety of Ma's Bamboo-based medicinal moxibustion therapy for chronic fatigue syndrome [411].<br>② Moxibustion at 'Danzhong' (RN17) and 'Guanyuan' (RN4) for fatigue symptom in patients with depression [414].<br>③ The microcirculatory characteristics of the heart and lung meridians [415.]
		1b<br>Specify the intervention, in terms of 1) CHMF, 2) acupuncture, 3) moxibustion, or 4) other TCM therapy(s).	Q2. Whether the specific intervention was reported in the title?	① "Fully Reported" was considered if the title explicitly denotes the specified intervention as moxibustion.<br>② "Partially Reported" was considered if the title did not provide sufficient information to determine whether the intervention was of moxibustion.<br>③ "Not Reported" was considered if the title does not refer to the specified intervention.	① Efficacy and safety of Ma's Bamboo-based medicinal moxibustion therapy for chronic fatigue syndrome [411].<br>② Moxibustion at 'Danzhong' (RN17) and 'Guanyuan' (RN4) for fatigue symptom in patients with depression [414].<br>③ The microcirculatory characteristics of the heart and lung meridians [415].

2	Background and Rationale	6a.1<br>Provide the background and rationale of the research question with TCM theory.	Q3. Whether the rationale of TCM about moxibustion intervention for diseases or TCM patterns was reported in the background/introduction?	① "Fully Reported" was considered if the background and introduction of this report provide a clear explanation of the theoretical principles in Chinese medicine that led to the selection of moxibustion as a treatment for the disease or condition under investigation, ensuring that the underlying theory is transparently presented.<br>② "Partially Reported" was considered if the background and introduction, the selection of moxibustion as a therapeutic intervention for the disease or condition is supported by a comprehensive account of its theoretical underpinnings rooted in Chinese medicine theory.<br>③ "Not Reported" was considered if the background and introduction provided a description of the characteristics of moxibustion and the disease, without elucidating their relationship.	① A great deal of researches either domestic or foreign in recent years has proven that moxibustion is an important approach to treat AS, with the techniques against this disease including long snake moxibustion,indirect moxibustion, warming needle moxibustion and heat-sensitive moxibustion, all of which have thermal stimulus despite their difference in manipulating the methods and components. Thunder-fire moxibustion has the advantages such as richer components of drugs, higher temperature, greater thermal radiation, and stronger penetration, in comparison with ordinary moxibustions. Certain efficacy has been identified by researches for thunder-fire moxibustion in treating pain and osteoarthropathy [409].<br>② According to the theories of acupuncture and traditional Chinese medicine, meridians distribute on the surface of the whole body vertically and horizontally, integrating the surface of the body with internal organs, thus transforming the whole body into one entire organ. That is to say, the essence of the meridian theory and meridian systems mainly manifests its summaries concerning the fundamental rules for correlation and

					specificity of different sites of the body. After many years of effort, although the physical structure of meridians has not been found, the biological characteristics of meridians has been confirmed, which could be used as the entry point for meridian studies. However, the majority of the existing studies involve lots of subjective assessments. Moreover, few studies have investigated the site specificity between 2 specific meridians [415].
		6a.2<br>Describe the rationale of the utilized TCM interventions with references.	Q4. Whether the rationale of moxibustion intervention was reported in the background/introduction?	① “Fully Reported” was considered if the specific rationale for the moxibustion intervention should be clearly stated in the background and introduction, supported by reliable sources such as historical documented accounts, previous studies, or expert consensus, accompanied by appropriate references.<br>② “Partially Reported” was considered if only the principles of the moxibustion intervention are presented in the background and introduction, while lacking references.<br>③ “Not Reported” was considered if the rationale and references for the moxibustion intervention were not adequately addressed in the background and introduction.	① Moxibustion is a traditional Chinese medicine (TCM) method of the treatment of KOA. Unlike drug treatment, moxibustion rarely causes side effects, which can effectively relieve the pain symptoms of KOA patients,[13–15] and improve the overall function. In a meta-analysis of a randomized controlled trial involving 11 moxibustion treatments for KOA, the moxibustion group had significantly improved overall pain and physical function scores compared to the traditional oral medication [416].<br>② Previous systematic reviews have confirmed that moxibustion or acupuncture combined with conventional Western medicine is better than traditional treatment group [418].

3		6b<br>Describe the rationale and principle(s) for selecting comparators corresponding to certain interventions (i.e. CHMFs, acupuncture, moxibustion or other TCM interventions), considering 1) comparable with tested intervention; 2) success of blinding.	Q5. Whether the he rationale and principle(s) for selecting comparators corresponding to moxibustion intervention was reported?	① "Fully Reported" was considered if the text provides comprehensive reporting on the selected types of controls, as well as the rationale and justification for their implementation, regardless of their location.<br>② "Partially Reported" was considered if only the type of control (e.g., sham moxibustion control, blank control, waiting list, etc.) was briefly described, with no mention of the rationale or justification for setting up the control.<br>③ "Not Reported" was considered if the full text does not report on the type of control chosen and the rationale and justification for the control.	① At present, pharmacological treatment remains the major treatment option for RA patients. These include non-steroidal anti-inflammatory drugs (NSAIDs), glucocorticoids, disease-modifying antirheumatic drugs (DMARDs), Janus kinase (JAK) inhibitors and Biosimilars [9] [404].<br>② In practice, analgesics are standard primary treatments for CSR unless there is evidence of spinal cord disease or apparent inability to move. Nonsteroidal antiinflammatory drugs (NSAIDs) could relieve pain as firstline agents in acute settings. The efficacy of ibuprofen in the treatment of cervical nerve root pain has also been reported, but it can only relieve pain and has no effect on the other symptoms of CSR [407].
3	Objectives	7<br>State the objectives or hypotheses regarding the specific TCM intervention for 1) a WM-defined disease, 2) a WM-defined disease with a specific TCM pattern or 3) a TCM pattern.	Q6. Whether the objectives or hypotheses regarding moxibustion intervention was reported?	① "Fully Reported" was considered if the purpose or hypothesis of the moxibustion study was explicitly reported as targeting a Western disease, or a TCM pattern, or the names of a Western disease and a TCM pattern; if the study targeted two or more TCM patterns, a generalized description such as "TCM diagnosis and treatment" appeared in the title and was considered fully reported.<br>② "Partially Reported" was considered if generalized descriptions were derived from	① In the treatment of CS neck pain, complementary therapies such as acupuncture, moxibustion, and massage have been widely accepted. Thunder-fire moxibustion, which contains refined moxa as well as agarwood, frankincense, costus root, and other traditional Chinese medicines, is often used in China to warm the meridian to relieve pain. However, the effect of thunder-fire moxibustion on CSR remains uncertain because of poor study

				the research objectives, encompassing diseases related to respiratory system and evidence-based treatment in Traditional Chinese Medicine (TCM), among others.<br>③ "Not Reported" was considered if it was not stated that the purpose or hypothesis of the moxibustion study was for a Western medicine disease, or a Chinese medicine evidence type, or a Western medicine disease with a Chinese medicine evidence type.	design and small sample sizes in previous clinical trials [407].<br>② Moxibustion is an external TCM treatment based on the meridian theory. It has been widely used in the treatment of various diseases including cardiovascular diseases and has shown good curative effects. The curative effect of moxibustion mainly comes from the thermal effect of moxa burning on acupoints on the body surface, and the chemical stimulation of the medicinal ingredients in the wormwood. Animal experiments have found that moxibustion plays a key role in myocardial protection by regulating neuroendocrine immune response, inhibiting excessive autophagy, and improving myocardial hypertrophy and cardiac function. The mechanism may be related to the upregulating of mTOR, inhibiting myocardial autophagy, and enhancing anti-inflammatory responses [418].
4	Eligibility Criteria	10a<br>State whether participants with a specific TCM pattern will be recruited, in terms of 1) diagnostic criteria, and 2) inclusion and exclusion criteria. All criteria	Q7. If participants with a specific TCM pattern would be recruited, whether the TCM diagnostic criteria, inclusion and exclusion criteria and reference(s) was reported?	⑤ "Fully Reported" was considered if the recruitment of subjects with specific TCM patterns should be accompanied by a comprehensive description of their diagnostic criteria as well as clear inclusion and exclusion criteria. It is essential to utilize recognized diagnostic criteria or provide references that can be consulted for detailed explanations.	① TCM diagnostic criteria IBS-D patients were diagnosed with spleen deficiency and dampness syndrome referring to the Expert Consensus Opinions on the Treatment of IBS in TCM 2017 [16]: primary symptoms: ① loose stools and diarrhea; ② vague abdominal pain; secondary symptoms: ① attacks or worsening after exertion or cold;

		utilized should be universally recognized, or reference(s) where detailed explanations can be found should be given.		⑥ "Partially Reported" was considered if it failing to meet any of the TCM diagnostic criteria, inclusion exclusion criteria, or references.。<br>⑦ "Not Reported" was considered if subjects meeting specific TCM evidence types were recruited; however, the TCM diagnostic criteria, inclusion/exclusion criteria, and references were not provided in the text<br>⑧ "Not applicable" was considered if refraining from recruiting subjects exhibiting a specific TCM evidence pattern.	② fatigue and lassitude; ③ anorexia. Tongue and pulse: Pale tongue with teeth marks on two sides, white and greasy fur; weak pulse [405].
		10b<br>Descriptions of the roles, qualifications and other relevant experience of the researchers (e.g., participant screeners, care providers, outcome assessors, data analysts) in TCM research are recommended.	Q8. Whether the roles, qualifications and other relevant experience of the researchers was reported?	④ "Fully Reported" was considered if the roles, qualifications, and relevant experience of individuals involved in the study (e.g., subject screeners, healthcare providers, outcome assessors, data analysts) were explicitly documented.<br>⑤ "Partially Reported" was considered if only a concise portrayal of the researcher is provided, for instance, the practitioner is solely identified as a physician without elaborating on their qualifications and pertinent expertise.<br>⑥ "Not Reported" was considered if the roles, qualifications, and relevant experience of individuals involved in the study (e.g., subject screeners, healthcare providers, outcome assessors, data analysts) were not reported.	① All CRFs will be stored in a locked cabinet. At the end of the study, the investigator will submit the CRFs to the data management committee, and the investigators cannot modify the data. The data monitoring committee is independently chaired by the Statistics Teaching and Research Office of Changshu Hospital Affiliated to Nanjing University of Chinese Medicine and claims no conflict of interest [403].<br>② The data monitoring committee is independently chaired by the Statistics Teaching and Research Office of Guangzhou University of Chinese Medicine and claims to have no conflict of interest. The South China Research Center for Acupuncture and Moxibustion will act as an independent

					committee to monitor the progress and provide advice if necessary. The Ethics Committee of the First Affiliated Hospital of Guangzhou University of Chinese Medicine will take part in endpoint adjudication. All staff members will be included in the author's contribution [407].<br>③ Thunder-fire moxibustion will be performed by a licensed acupuncturist with more than 6 years of experience who will be trained before the trial [409].
		10c<br>Descriptions of the qualification and relevant experience of study center(s) involved in a TCM trial are recommended.	Q9. Whether the qualification and relevant experience of study center(s) involved in a TCM trial was described?	④ "Fully Reported" was considered if The reported availability of qualifications and relevant experience of research centers in their participation in TCM trials.<br>⑤ "Partially Reported" was considered if the research center where the TCM trial was conducted is specified by providing the name and credentials, such as patient recruitment exclusively at XX hospitals or solely focusing on recruitment at a tertiary hospital.<br>⑥ "Not Reported" was considered if it failure to provide comprehensive reporting on the qualifications and relevant experience of research centers involved in clinical trials of Chinese medicine.	This entry has not been fully reported in any study, and examples of partial reporting are available for reference:<br>① We will recruit participants by advertising on bulletin boards, located at the Department of Orthopedics, the Department of Acupuncture and Characteristic Chinese Medicine, and the Department of Rehabilitation Nursing Center at the First Affiliated Hospital of Guangzhou University of Chinese Medicine [407].
5	Interventions	11a.1<br>Interventions for the experimental group(s)	Q10. Whether the patient posture during the moxibustion treatment	① "Fully reported" was considered if there was information related to the position/posture of the patient during the	① In the quiet treatment room, the participant should be in a prone position. One moxa stick will be held by the operator

		with sufficient detail to allow replication.	and the treatment environment were reported?	treatment and the treatment environment. For the patient position/posture during the moxibustion treatment, it could be prone, sitting, lying sideways, the posture of the patient of both sides of the treatment if both front and back points are used, "Any position was acceptable as long as the patient was comfortable", "There were no special requirements for position/posture", or other expressions with similar meanings in the article, etc. For the treatment environment, it could be descriptions about temperature, humidity, noise, light, etc.<br>② "Partially Reported" was considered if only one therapeutic environment or subject position for moxibustion was documented.<br>③ "Not Reported" was considered if the therapeutic context and the position of the subject receiving moxibustion were not specified in the report.	who will ignite the top of the stick. Then, the moxa stick will be placed on the treatment site, and the fire head will be 3 cm away from the skin [412].
			Q11. Whether the name and number (uni/bilateral) of acupoints/locations used for moxibustion was reported?	① "Fully reported" was considered if there was information related to the name and number of acupoints/locations (if no official name). For the name, they could be standard nomenclature for acupoints, anatomical locations, "The pain site was selected for	① Points of Qihai (CV 6), Zhongji (CV 5), Guanyuan (CV 4), and bilateral Sanyinjiao (SP 6) will be selected for moxibustion [403].<br>② The sites for thunder-fire moxibustion will be selected along the governor meridian in its spinal

				moxibustion treatment” reported in the article, or other expressions with similar meanings. For the number, it referred to whether moxibustion was carried out unilaterally or bilaterally.<br>② “Partially Reported” was considered if only the number of moxibustion materials used per treatment unit or the name or location of the acupoints used (unilateral/bilateral) was reported, e.g., bilateral acupoints, but only the name of the acupoint was reported without unilateral or bilateral.<br>③ “Not Reported” was considered if the number of moxa materials used, the name and location of acupoints used (unilateral/bilateral) per treatment unit were not reported for each subject.	segment, with the acupoints Dazhui and Yaoshu as the borders. All acupoints will be located according to the National Standard of the People’s Republic of China: Name and Location of Acupoints (GB/T 12346-2006) issued in 2006, wherein acupoint Dazhui (GV14) is located in the infraspinous depression of the 7th cervical vertebra along the posterior middle line within the spinal region and acupoint Yaoshu (GV2) is at a place along the posterior middle line within the sacral region and opposite to sacral hiatus (Fig1)[409].

					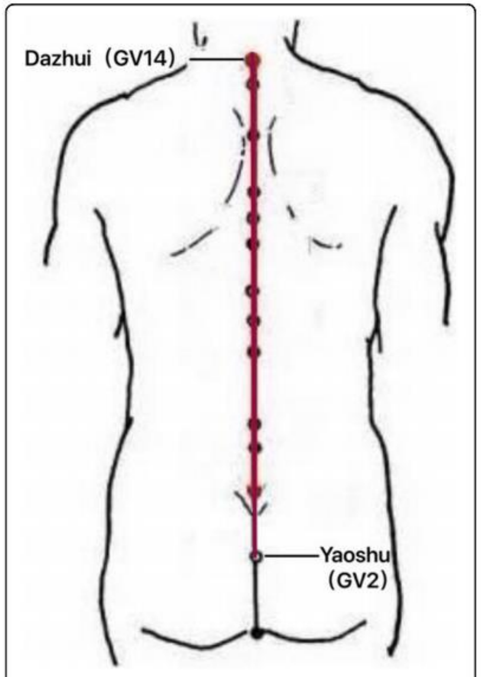<br>**Fig. 1** The sites for thunder-fire moxibustion. The sites will be selected along the governor meridian in its spinal segment, with the acupoints Dazhui and Yaoshu as the border
			Q12. Whether the Procedure and technique of moxibustion (e.g., direct/ indirect, warming/sparrow-pecking technique, warming needle, moxa box) and Response sought (e.g., warm feeling, skin reddening, burning pain) were	④ "Fully reported" was considered if 1) readers can identify how the moxibustion procedure and technique were performed, or the moxibustion procedure without special technique was clearly descried. **AND** 2) descriptions of responses from participants, either the expected responses or the actual	①The operational methods of thunder-fire moxibustion are as follows: the patient is kept in a prone position, and the selected acupoints are exposed. The moxibustion medicine fixed in small holes of the moxibustion box is ignited, and the box is placed onto the position of the governor

			described?	responses. It was also considered as "Fully reported" if authors reported "No special response was found", "All participants responded normally", or other expressions with similar meanings.<br>⑤ "Partially Reported" was considered if 1) readers can identify how the moxibustion procedure and technique were performed, or the moxibustion procedure without special technique was clearly descried. **OR** 2) descriptions of responses from participants, either the expected responses or the actual responses. It was also considered as "Fully reported" if authors reported "No special response was found", "All participants responded normally", or other expressions with similar meanings.<br>⑥ "Not Reported" was considered if the documentation of moxibustion procedures, practices, and the corresponding physiological responses was not reported in the reported literature.	meridian from acupoint Dazhui to that of Yaoshu at the back, where it is covered with a towel for a warm moxibustion for 30 min. The medicine is removed once every 15 min to blow away the ashes. The moxibustion is continued until redness appears on the skin and deep tissue becomes warm, with local perspiration [409].<br>② The operational methods of thunder-fire moxibustion are as follows: the patient is kept in a prone position, and the selected acupoints are exposed. The moxibustion medicine fixed in small holes of the moxibustion box is ignited, and the box is placed onto the position of the governor meridian from acupoint Dazhui to that of Yaoshu at the back, where it is covered with a towel for a warm moxibustion for 30 min. The medicine is removed once every 15 min to blow away the ashes. The moxibustion is continued until redness appears on the skin and deep tissue becomes warm, with local perspiration [410].
			Q13. Whether the materials used for moxibustion was reported?	① "Fully reported" was considered if there was information related to the materials used for moxibustion, such as moxa floss (white, soft, cotton-like fibers prepared from moxa	① The moxa rolls for moxibustion are produced by Nanyang Chinese Medicine Airong Co. Ltd., in 18 × 200 mm [403].

				leaves), moxa cone (cone-like shape made of moxa wool and commonly with three different sizes), moxa stick (strick wrapping moxa floss and some other herbs), indirect moxibustion materials (ginger, garlic, salt, monkshood cake, medicinal herbs and paper), special apparatuses (moxa burner), special form of moxibustion using herbs other than moxa (natural moxibustion—Tianjiu), etc. And indicate the manufacturer or production lot number of the material.<br>② "Partially Reported" was considered if there was information related to the materials used for moxibustion, such as moxa floss (white, soft, cotton-like fibers prepared from moxa leaves), moxa cone (cone-like shape made of moxa wool and commonly with three different sizes), moxa stick (strick wrapping moxa floss and some other herbs), indirect moxibustion materials (ginger, garlic, salt, monkshood cake, medicinal herbs and paper), special apparatuses (moxa burner), special form of moxibustion using herbs other than moxa (natural moxibustion—Tianjiu), etc. or indicate the manufacturer or production lot number of the material.<br>③ "Not Reported" was considered if there is no information was reported on any moxibustion therapy materials.	② Three grams of moxa (Suzhou Medical Appliance Factory, Suzhou, Jiangsu, China) will be squeezed into olive-shaped sticks, which will be placed on the compressed powder and ignited for preheating [411].

			Q14. Whether the number, frequency and duration of the moxibustion sessions were reported?	① "Fully reported" was considered if there was information related to the number of session, frequency and duration of each treatment or the length of the entire treatment, either planned or actual.<br>② "Partially reported" was considered if there reporting on only one or two part of number, frequency and duration.<br>③ "Not Reported" was considered if was no information about the number, frequency and duration of the cupping sessions.	① Moxibustion is performed on the local neck area for a treatment of 30 min. The moxibustion box and the treatment area are covered with a thick treatment towel to maintain temperature and to control smoke (Figs. 2 and 3). Treatments will be given 5 days per week for 2 weeks [407].<br>② Thunder-fire moxibustion will be performed three times a week successively for 4 weeks with 12 times in total. The moxibustion medicine fixed in small holes of the moxibustion box is ignited, and the box is placed onto the position of the governor meridian from acupoint Dazhui to that of Yaoshu at the back, where it is covered with a towel for a warm moxibustion for 30 min. The medicine is removed once every 15 min to blow away the ashes [409].
		11a.2<br>Describe interventions for the control group(s) with sufficient detail to allow replication.	Q15. Whether the state any special arrangement(s) in pre-treatment, treatment and post-treatment periods corresponding to the experimental intervention (e.g., examinations in pre-treatment period, unaltered lifestyle and medication in treatment period, and compensatory interventions in post-treatment period) were reported?	① "Fully reported" was considered if the choice of a blank control or waiting group control for the study protocol requires a description of the special arrangements for the pre-treatment, treatment and post-treatment periods corresponding to the trial intervention.<br>② "Partially reported" was considered if the study protocol employed either a blank	No cases meeting the fullly reported criteria were identified in the articles included.

				control or a waitlist control; however, certain aspects pertaining to the implementation of the blank or waiting group control were not provided.<br>③ "Not Reported" was considered if the study protocol chose either a blank control or a waiting group control, but some of the details of the blank or waiting group control are missing.<br>④ "Not applicable" was considered if there was no blank control or a waitlist control.	
			Q16. Whether the state the comparability of the sham moxibustion or moxibustion-like control and comprehensively provide details as for the recommendations of Intervention were reported?	① "Fully reported" was considered if describe the similarity of the sham or moxibustion-like control to the test group moxibustion and describe the sham or similar moxibustion in detail<br>② "Partially reported" was considered if the study protocol included sham moxibustion or moxibustion-like control; However, certain aspects of the control protocol remain undisclosed.<br>③ "Not Reported" was considered if the study mentioned the use of sham moxibustion or moxibustion-like controls, but failed to provide any pertinent details regarding the control protocol.	① In the zero MSC group, all four devices will each contain one lit stick of smokeless charcoal moxa. In the low MSC group, only one device will contain one lit stick of conventional moxa floss, whereas the other three devices will each contain one lit stick of smokeless charcoal moxa. In the medium MSC group, two devices will each contain one lit stick of conventional moxa, and two devices will each contain one lit stick of smokeless charcoal moxa. In the high MSC group, all four devices will each contain one lit stick of conventional moxa. Each moxibustion treatment will last for 20 minutes per session, with 5 weekly sessions being conducted over the course of 6 weeks [413].

				④ "Not applicable" was considered if the study protocol had no sham moxibustion or moxibustion-like control.	
		11d.2<br>Descriptions of other interventions that will be administrated to experimental and/or control groups are recommended (e.g., rescue interventions), with enough details to allow replication.	Q17. Whether the details of other interventions administered to experimental and/or control groups were reported?	⑤ "Fully reported" was considered if there was information related to the details of other interventions administered to the moxibustion group, such as acupuncture, cupping, herbs, exercises, or lifestyle advice, either carried out by the treatment provider or the patient.<br>⑥ "Partially reported" was considered if there was insufficient information about the details of other interventions administered to the moxibustion group.<br>⑦ "Not Reported" was considered if there was no information about the details of other interventions administered to the moxibustion group.<br>⑧ "Not applicable" was considered that the experimental and/or control groups received only moxibustion treatment.	① Based on the 2018 Chinese guideline for the diagnosis and treatment of rheumatoid arthritis, patients in both groups will be administered 12.5 mg methotrexate once per week (2.5 mg/pill, 030170202, SPH Sine Pharmaceutical Laboratories Co., Ltd, Shanghai, China). An additional 2.5 mg of methotrexate will be administered if patients experience uncontrolled pain, swelling or stiffness. The maximum dose will not exceed 15 mg. Therapy will last for three months [404].<br>② Gentle Moxibustion Group. In this group, Zhongwan (RN12), Guanyuan (RN4), and Sanyinjiao (SP6) will be selected (Figure 3). In order to keep the local skin's surface temperature at 45°C± 2°C for 20 minutes, a lit moxa stick (1.5 cm diameter moxa cone grown in Qichun County, Hubei, China) will be placed 2–3 cm away from the skin. For 12 weeks straight, a gentle moxibustion procedure will be performed every other day, three times per week. During the menstrual cycle, the treatment will be postponed [410].

6	Outcomes	12a<br>Provide the rationale of TCM-related indexes as outcomes (e.g. the change of degree and scope of symptoms and signs related to pattern differentiation).	Q18. Whether the rationale of TCM-related indexes as outcomes were reported?	① "Fully reported" was considered if the rationale and references for utilizing TCM-related indicators as outcome measures should be concurrently provided. The rationale may encompass the study's objective, previous research findings, or specific literature citations.<br>② "Partially reported" was considered if the TCM-related outcome indicators are briefly described without any accompanying references, thereby lacking scholarly and academic rigor.<br>③ "Not Reported" was considered if the rationale for employing TCM-related indicators as outcome measures was not documented.	① A change in the mean severity of hypertensive symptoms scored by the symptom scale in the Guiding Principles of Clinical Research on New Drugs of Chinese Medicines at month 6. The scale assesses 24 hypertension-related symptoms. Each item receives a score of 0 to 3, and a higher score indicates higher severity of that symptom; the total score is the sum of the individual item scores and ranges from 0 to 72 (see details in Additional file 1) [408].<br>② TCM syndrome scale score. The IBS-D TCM symptom scoring criteria refers to the Guidelines for Clinical Research on New Chinese Medicines (Trial). Higher score indicates more severe symptoms, and the scoring details are shown in Table 2. Perform score at baseline, after 4 weeks of treatment, and at week 4 of follow-up once each [405].
		12b<br>Provide the details of the TCM-related outcomes assessment, including i) the measuring methods and standard (e.g. frequency, severity rating scale of symptoms and signs, verified pattern questionnaire, time	Q19. Whether the details of the TCM-related outcomes assessment were described?	④ "Fully reported" was considered if the study comprehensive elucidation of the evaluation process for outcome indicators related to Chinese medicine, encompassing measurement methods and criteria, assessor qualifications, strategies for enhancing assessment quality, and pertinent references.<br>⑤ "Partially reported" was considered if	① TCM syndrome scale score. The IBS-D TCM symptom scoring criteria refers to the Guidelines for Clinical Research on New Chinese Medicines (Trial). Higher score indicates more severe symptoms, and the scoring details are shown in Table 2. Perform score at baseline, after 4 weeks of treatment, and at week 4 of follow-up once each [405].

		points for assessment and corresponding rationale), ii) assessor qualification (e.g. relevant assessment experience, years in clinical practice), iii) methods used to enhance the quality of assessment (e.g. multiple repeated observation, training of assessors), and iv) related reference(s).		only names or references of TCM-related outcome indicators were reported<br>⑥ "Not Reported" was considered if the rationale for employing TCM-related indicators as outcome measures was not documented.	② The efficacy of TCM syndrome will be evaluated by the TCM Syndrome Score (TCMSS) and TCM syndrome effective rate as the standard assessment. With the TCMSS, TCM syndrome is determined by stiffness and pain of the lumbar, sacro, ack, cervical spine and hip, fatigue, and chills (see more details in Additional file 1). The scores range from 0 to 14. Then, we will calculate the relative TCMSS by using the nimodipine method: TCMSSb stands for the TCMSS before treatment, and TCMSSa is the TCMSS after treatment: relative TCMSS = (TCMSSb − TCMSSa)/ TCMSSb × 100%. The specific methods to assess the efficacy of TCM syndrome are as follows: (1) clinical cure: the main TCM symptoms and signs disappear, relative TCMSS ≥ 85%; (2) markedly effective: the main TCM symptoms and signs improved obviously, 65 ≤ relative TCMSS < 85%; (3) effective: the main TCM symptoms and signs improved, 30 ≤ relative TCMSS < 65%; and (4) ineffective: the main TCM symptoms and signs not improved and even aggravated, relative TCMS S < 30%. Counting the number of patients who were clinically cured and markedly effective, and denoted by n1 and n2. Calculating the TCM syndrome effective rate using the following

					formula: TCM syndrome effective rate = (n1 + n2)/n × 100%, where n stands for the total number of patients in this study [409].
7	Data Collection Methods	18a<br>When trial targeting TCM pattern, or a WM-defined disease with a specific TCM pattern, baseline data about TCM pattern should be provided.	Q20. If trial targeting TCM pattern, or a WM- defined disease with a specific TCM pattern, whether the baseline data about TCM pattern was provided?	⑤ "Fully reported" was considered if The baseline data collection encompasses comprehensive information on evidence in traditional Chinese medicine (TCM), including baseline characteristics and disparities among populations with different types of evidence.<br>⑥ "Partially reported" was considered if it was only mentioned in the baseline data collection or statistics that the evidence type would be considered, but there were no details on how this would be implemented.<br>⑦ "Not Reported" was considered if the baseline data did not include information regarding the evidence of TCM.<br>⑧ "Not applicable" was considered if the study focuses on the disease rather than the evidence pattern of non-Chinese medicine.	① This prospective, parallel, randomized controlled trial (RCT) will comfirm mild-warm moxibustion on IBS-D in efficacy and safety. And syndrome combing spleen deficiency and dampness will be observed. Patients will be randomly assigned to receive 4 weeks of mild-warm moxibustion treatment or oral loperamide hydrochloride capsules therapy, followed by 4 weeks of follow-up in both groups. See trial's flow diagram in Figure 1 and schedule of protocol in Table 1. The Consolidated Standards of Reporting Trials 2017 guided this protocol, and also Standard Protocol Items: Recommendations for Interventional Trials (Table S1). TCM diagnostic criteria. IBS-D patients were diagnosed with spleen deficiency and dampness syndrome referring to the Expert Consensus Opinions on the Treatment of IBS in TCM 2017 [16]: primary symptoms: ① loose stools and diarrhea; ② vague abdominal pain; secondary symptoms: ① attacks or worsening after exertion or cold; ② fatigue and lassitude; ③ anorexia. Tongue and pulse: Pale tongue with teeth marks on two sides, white and greasy fur; weak pulse

					[405].

Supplementary file 4: List of the included protocols

1. Lu W, Giobbie-Hurder A, Tanasijevic A, Baedorf Kassis S, Park SH, Jeong YJ, et al. Acupuncture for hot flashes in hormone receptor-positive breast cancer, a coordinated multinational study: Rationale and design of the study protocol. Contemp Clin Trials. 2022;121:106885.
2. Wang XX, Liu CF, Wei XQ, Zheng YY, Li FY, Fang Y, et al. Acupuncture versus sham acupuncture in the treatment of diabetic distal symmetric polyneuropathy (DSPN): Study protocol for a randomized controlled trial. European Journal of Integrative Medicine. 2020;34.
3. Yin ZH, Zhang XY, Li YQ, Wang ZQ, Ye F, He X, et al. Effect and neuroimaging mechanism of acupuncture for amnestic mild cognitive impairment: Study protocol of a multicenter randomized controlled trial. European Journal of Integrative Medicine. 2023;58.
4. Wang L, Xu T, Sun M, Wan S, Ying J, Luo L, et al. Electro-acupuncture for gastrointestinal dysfunction after colorectal cancer surgery: A protocol for three-arm randomized controlled trial. European Journal of Integrative Medicine. 2023:102277.
5. Li Y, Ma Y, Guo W, Ge W, Cheng Y, Jin C, et al. Effect of transcutaneous electrical acupoint stimulation on postoperative pain in pediatric orthopedic surgery with the enhanced recovery after surgery protocol: a prospective, randomized controlled trial. Anaesth Crit Care Pain Med. 2023;42(6):101273.
6. Nagato L, Leal AF, de Moraes IAP, Barros AFF, Dias AE, Canzonieri AAM, et al. Effect of acupuncture on heart rate variability in individuals with multiple sclerosis: a protocol for a double-blinded randomized controlled trial. Journal of Human Growth and Development. 2023;33(1):44.
7. Chen J-M, Chen C-T, Wu B-Y, Chen Y-H, Liu C-T. Effects of Laser Acupuncture on Constipation in Patients With Advanced Cancer: Study Protocol for a Double-blinded, Randomized Controlled Trial. 2021.
8. 8.Afshari Fard MR, Mohammadi A, Ma LX, Mu JD, Yu WY, Song Y, et al. Comparison of the immediate analgesic effect of perpendicular needling and transverse needling at SP6 in patients with primary dysmenorrhea: study protocol for a randomized controlled trial. Medicine. 2020;99(3):e18847.
9. Zhao Q, Zheng S, Delaney GP, Moylan E, Agar MR, Koh ES, et al. Acupuncture for Cancer Related Pain: protocol for a Pragmatic Randomised Wait-List Controlled Trial. Integrative cancer therapies.2020;19:1534735420976579.
10. .Zhou J, Liu S, Sun Y, Wang W, Liu Z. Efficacy of acupuncture for treatment of intermittent claudication in patients with degenerative lumbar spinal stenosis: protocol for a randomized controlled trial. Trials. 2020;21(1):679.
11. Huang M, Lai M, Wang X, Wang Y, Yang J, Yang Z, et al. Acupuncture for osteoporosis: study protocol for a randomized controlled trial. 2020.
12. Sun J, Meng X, Oleson T, Wang L, Li C, Zhang L, et al. Auricular Acupuncture Therapy for Stage I of Shoulder Hand Syndrome (SHS) After Ischemic Stroke: Study Protocol for a Randomized, Sham-controlled, Patient-blinded, Clinical Trial. 2021.
13. Sun Y, Liu Y, Su T, Sun J, Wu Y, Liu Z. Electroacupuncture versus solifenacin for women with urgency-predominant mixed urinary incontinence: a protocol for a three-armed non-inferiority randomized controlled trial.BMC Complement Med Ther. 2020;20(1):18.
14. Wen Q, Li N, Wang X, Li H, Tian F, Chen W, et al. Effect of electroacupuncture versus solifenacin for moderate and severe overactive bladder: a multi-centre, randomized controlled trial study protocol. BMC Complement Med Ther. 2020;20(1):224.
15. Zhong LLD, Wu X, Lam TF, Wong YP, Cao P, Wong EY, et al. Electro-acupuncture for central obesity: randomized, patient-assessor blinded, sham-controlled clinical trial protocol. BMC Complement Med Ther. 2021;21(1):190.
16. Yu C, Wang J, Shen B, Li X, Zhang R, Qin Y, et al. Effectiveness of acupuncture in the treatment of cyclic mastalgia: a study protocol for a randomized controlled trial. BMC Complement Med Ther. 2022;22(1):297.
17. Zhou Y, Shen Y, Ye X, He D, Sun N, Zhang Y, et al. Acupuncture on GB34 for immediate analgesia and regulating pain-related anxiety for patients with biliary colic: a protocol of randomized controlled trial. BMC Complement

Med Ther. 2023;23(1):224.
18. Chang XL, Liu XM, An LX, Zheng JY, Zhang K. Effects of transcutaneous electrical acupoint stimulation (TEAS) on postoperative pain in patients undergoing gastric and esophageal ESD surgery: a study protocol for a prospective randomized controlled trial. BMC Complement Med Ther. 2023;23(1):253.
19. Chang X, Zhu Y, Zhao W, Liu Y, He Y, Chen Y, et al. Electro-acupuncture for health-related quality of life and symptoms in patients with gastric cancer undergoing adjuvant chemotherapy (EAGER): a protocol for a multicenter randomized controlled trial. Health Qual Life Outcomes. 2023;21(1):70.
20. Wang W, Yu S, Long Z, Liu Y, Yan Y, Sun T, et al. Electroacupuncture vs topical diclofenac sodium gel for patients with hand osteoarthritis: study protocol for a randomized controlled trial. J Orthop Surg Res. 2022;17(1):233.
21. Fu J, Cai X, Ouyang H, Gong C, Huang Y. Efficacy of electroacupuncture in assisting postoperative healing of distal radius fractures: study protocol for a randomized controlled trial. J Orthop Surg Res. 2022;17(1):517.
22. Wang W, Shi H, Liu Y, Sun Y, Chen Y, Liu Z. Efficacy and safety of acupuncture for hand osteoarthritis: study protocol for a multi-center, randomized, sham-controlled clinical trial. J Orthop Surg Res. 2023;18(1):89.
23. Guo L, Huang X, Ha LJ, Zhang JZ, Mi J, Sun PH, et al. Efficacy of compatible acupoints and single acupoint versus sham acupuncture for functional dyspepsia: study protocol for a randomized controlled trial. Trials.2020;21(1):77.
24. Chan K, Lui L, Yu K, Lau K, Lai M, Lau W, et al. The efficacy and safety of electro-acupuncture for alleviating chemotherapy-induced peripheral neuropathy in patients with coloreactal cancer: study protocol for a single-blinded, randomized sham-controlled trial. Trials. 2020;21(1):58.
25. Wang J, Shi W, Khiati D, Shi B, Shi X, Luo D, et al. Acupuncture treatment on the motor area of the scalp for motor dysfunction in children with cerebral palsy: study protocol for a multicenter randomized controlled trial. Trials. 2020;21(1):29.
26. Zhang ZQ, Li KP, He J, Jiang LM, Wang W, Hu XS, et al. Acupuncture of fascia points to relieve hand spasm after stroke: a study protocol for a multicenter randomized controlled trial. Trials. 2020;21(1):69.
27. Li S, Zhang Q, Yin X, Yue H, Zhang W, Lao L, et al. Electroacupuncture for postoperative pain after nasal endoscopic surgery: study protocol for a pilot randomized controlled trial. Trials. 2020;21(1):163.
28. Chang YY, Chiu CW, Chen CY, Chang CF, Lee TC, Lo LC, et al. Efficacy of electroacupuncture on acute abdomen emergency care: study protocol for a randomized controlled trial. Trials. 2020;21(1):224.
29. Wu P, Cheng C, Song X, Yang L, Deng D, Du Z, et al. Acupoint combination effect of Shenmen (HT 7) and Sanyinjiao (SP 6) in treating insomnia: study protocol for a randomized controlled trial. Trials. 2020;21(1):261.
30. Han M, Cui J, Xiao Y, Xiao D, Jiao J, Peng Q, et al. Acupuncture for primary fibromyalgia: Study protocol of a randomized controlled trial. Trials. 2020;21(1):538.
31. Yu S, Dong X, Sun R, He Z, Zhang C, Chen M, et al. Effect of acupuncture and its influence on cerebral activity in patients with persistent asthma: study protocol for a randomized controlled clinical trial. Trials. 2020;21(1):406.
32. Jiao R, Huang M, Zhang W, Liu Z. Efficacy of acupuncture in improving the symptoms and the quality of life of patients with moderate or severe acne vulgaris: study protocol for a randomized controlled trial. Trials. 2020;21(1):563.
33. Bai YF, Gao C, Li WJ, Du Y, An LX. Transcutaneous electrical acupuncture stimulation (TEAS) for gastrointestinal dysfunction in adults undergoing abdominal surgery: study protocol for a prospective randomized controlled trial. Trials. 2020;21(1):617.
34. Tian ZX, Liu CZ, Qi YS, Tu JF, Lin Y, Wang Y, et al. Transcutaneous electrical acupoint stimulation for stage 1 hypertension: protocol for a randomized controlled pilot trial. Trials. 2020;21(1):558.
35. Zhang N, Li JL, Yan CQ, Wang X, Lin LL, Tu JF, et al. The cerebral mechanism of the specific and nonspecific effects of acupuncture based on knee osteoarthritis: study protocol for a randomized controlled trial. Trials. 2020;21(1):566.
36. Xu H, Kang B, Li Y, Xie J, Sun S, Zhong S, et al. Using electroacupuncture to recover muscle strength in patients with knee osteoarthritis after total knee arthroplasty: a study protocol for a double-blinded, randomized, and placebo-controlled trial. Trials. 2020;21(1):705.

37. Xu H, Zheng C, He L, Su T, Wang H, Li Y, et al. Effect of acupuncture on women with poor ovarian response: a study protocol for a multicenter randomized controlled trial. Trials. 2020;21(1):775.
38. Liu L, Lu Y, Yan XN, Yang SQ, Gong LP, Li LE, et al. Efficacy and safety of fire needle therapy for blood stasis syndrome of plaque psoriasis: protocol for a randomized, single-blind, multicenter clinical trial. Trials. 2020;21(1):739.
39. Zhou J, Zhao L, Meng L, Liang H, Zhou T, Ye S, et al. Acupuncture treatment for carotid atherosclerotic plaques: study protocol for a pilot randomized, single blinded, controlled clinical trial. Trials. 2020;21(1):768.
40. Yu L, Liu S, Zheng C, Liu W, Wang H, Liang F, et al. Manual acupuncture versus sham acupuncture and usual care for the prevention of primary dysmenorrhea (PD): study protocol for a randomized controlled trial.Trials. 2020;21(1):818.
41. Yue H, Zhou S, Wu H, Yin X, Li S, Liang T, et al. Efficacy and safety of electro-acupuncture (EA) on insomnia in patients with lung cancer: study protocol of a randomized controlled trial. Trials. 2020;21(1):788.
42. Xu JJ, Ren M, Zhao JJ, Wu JJ, Zhang SC, Zhong YB, et al. Effectiveness of theta and gamma electroacupuncture for post-stroke patients on working memory and electrophysiology: study protocol for a double-center, randomized, patient- and assessor-blinded, sham-controlled, parallel, clinical trial. Trials. 2020;21(1):910.
43. Deng H, Shu Y, Lv P, Zhao L, Cheng K, Zhang T, et al. Acupuncture for diabetic peripheral neuropathy: study protocol for a randomized, placebo-controlled trial. Trials. 2020;21(1):879.
44. Gao Y, Liu L, Li B, Guo J, Liu H, Wang S, et al. Evaluation of the efficacy and safety of fire needle compared to filiform needle on knee osteoarthritis: study protocol for a randomized controlled trial. Trials. 2020;21(1):911.
45. He Z, Yang Y, Wen Q, Yin T, Li Z, Ma P, et al. Acupuncture for chronic stable angina pectoris based on the theory of Meridian-Viscera Association: study protocol for a multicenter randomized controlled trial. Trials. 2020;21(1):915.
46. Cheng S, Dong X, Lan L, He Z, Yu S, Yang Y, et al. Acupuncture for chronic persistent asthma based on the theory of Meridian-viscera Association: study protocol for a multi-center randomized controlled trial in China. Trials. 2021;22(1):17.
47. Wen H, Xu S, Zeng J, Ge S, Liao Y, Tang C, et al. Effect of acupuncture for methadone maintenance treatment patients: study protocol of a randomized clinical trial. Trials. 2020;21(1):1003.
48. Su KQ, Liu ST, Li JY, Li RQ, Feng HL, Xue Y, et al. Effects of different acupuncture treatment methods on post-stroke cognitive impairment: study protocol for a multicenter randomized controlled trial. Trials. 2021;22(1):27.
49. Yu FT, Ni GX, Cai GW, Wan WJ, Zhou XQ, Meng XL, et al. Efficacy of acupuncture for sciatica: study protocol for a randomized controlled pilot trial. Trials. 2021;22(1):34.
50. Lin Y, Wang X, Li XB, Wu BQ, Zhang ZH, Guo WH, et al. Acupuncture for persistent atrial fibrillation after catheter ablation: study protocol for a pilot randomized controlled trial. Trials. 2021;22(1):35.
51. Li F, Lu H, Wang X, Zhang Q, Liu Q, Wang T. Effectiveness of electroacupuncture for thin endometrium in infertile women: study protocol for a single-blind, randomized controlled trial. Trials. 2021;22(1):73.
52. Wang S, Zhao J, Zeng W, Du W, Zhong T, Gao H, et al. Acupuncture for Hashimoto thyroiditis: study protocol for a randomized controlled trial. Trials. 2021;22(1):74.
53. Kim JH, Na CS, Park GC, Lee JS. Effects of different wavelengths of invasive laser acupuncture on chronic non-specific low back pain: a study protocol for a pilot randomized controlled trial. Trials. 2021;22(1):118.
54. Wang Y, Shi GX, Tian ZX, Liu JH, Qi YS, Tu JF, et al. Transcutaneous electrical acupoint stimulation for high-normal blood pressure: study protocol for a randomized controlled pilot trial. Trials. 2021;22(1):140.
55. Dietzel J, Hörder S, Habermann IV, Meyer-Hamme G, Hahn K, Ortiz M, et al. Acupuncture in diabetic peripheral neuropathy-protocol for the randomized, multicenter ACUDPN trial. Trials. 2021;22(1):164.
56. Fang WH, Wang GL, Liu Q, Ding X, Wang ZY, Wang XW, et al. Effect of 'hand and foot acupuncture with twelve needles' on hemiplegia patients with 'qi deficiency and blood stasis' syndrome in the convalescent stage of Ischaemic stroke: study protocol for a randomised controlled trial. Trials. 2021;22(1):215.
57. Qi LY, Wang Y, Wang LQ, She YF, Shi GX, Li Y, et al. Acupuncture for the treatment of diarrheal-predominant irritable bowel syndrome: study protocol for a pilot randomized controlled trial. Trials. 2021;22(1):253.
58. Li S, Liu J, Huang J, Luo D, Wu Q, Ning B, et al. Acupuncture for comorbid mild-moderate depression and chronic

musculoskeletal pain: study protocol for a randomized controlled trial. Trials. 2021;22(1):315.
59. Peng W, Hong X, Huangfu Y, Sun Z, Shen W, Feng F, et al. The cerebral mechanism of acupuncture for chronic insomnia with gastrointestinal disorder: protocol for a randomized controlled trial. Trials. 2021;22(1):386.
60. Wang M, Fu W, Meng L, Liu J, Wu L, Peng Y, et al. SWE and SMI ultrasound techniques for monitoring needling treatment of ankylosing spondylitis: study protocol for a single-blinded randomized controlled trial. Trials. 2021;22(1):385.
61. Sun N, Zhou YF, Zhou J, Zuo WW, Ye XY, Deng XD, et al. The cerebral mechanism underlying the acupoints with specific effect for gallbladder stone disease: protocol for a randomized controlled task-fMRI trial. Trials. 2021;22(1):399.
62. Luo H, Peng J, Ma Q, Wei Z, Lin C, Zhang M, et al. Intradermal acupuncture for rheumatoid arthritis: study protocol for a randomised controlled trial. Trials. 2021;22(1):450.
63. Fan H, Hong X, Zeng J, Wang X, Chen J. Differences in the individual curative effect of acupuncture for obese women with polycystic ovary syndrome based on metagenomic analysis: study protocol for a randomized controlled trial. Trials. 2021;22(1):454.
64. Nie N, Chen L, Li T, Zhou C, Li B, Ji C, et al. Comparative effect of electroacupuncture with different frequency on headache attacks in migraine outpatients: study protocol for a randomised placebo-controlled trial. Trials. 2021;22(1):483.
65. Sun JW, Sun ML, Li D, Zhao J, Shi SH, Li HX, et al. Efficacy of acupuncture based on acupoint combination theory for irritable bowel syndrome: a study protocol for a multicenter randomized controlled trial. Trials. 2021;22(1):719.
66. Xu M, Zi Y, Wu J, Xu N, Lu L, Liu J, et al. Effect of opposing needling on motor cortex excitability in healthy participants and in patients with post-stroke hemiplegia: study protocol for a single-blind, randomised controlled trial. Trials. 2021;22(1):481.
67. Gao Z, Yin T, Lan L, Li D, Sun R, Ha G, et al. Efficacy and central mechanism of acupuncture treatment in patients with neck pain: study protocol for a randomized controlled trial. Trials. 2021;22(1):536.
68. Zhong LLD, Lam TF, Yang W, Zheng Y, Lyu Z, Bian Z. Electro-acupuncture for irritable bowel syndrome patients: study protocol for a single-blinded randomized sham-controlled clinical trial. Trials. 2021;22(1):619.
69. Shao JK, Liu Q, Pei W, Wang Y, Yang NN, Qi LY, et al. Electroacupuncture for postoperative ileus after laparoscopic surgery on colorectal cancer: study protocol for a randomized controlled trial. Trials. 2021;22(1):610.
70. Qin Y, Yang L, Zhang M, Bai Y, Li Z, Zhao N, et al. Efficacy evaluation and mechanism study of electroacupuncture intervention in acute phase of IFP: study protocol for a randomized controlled trial. Trials. 2021;22(1):663.
71. Sheng X, Yue H, Zhang Q, Chen D, Qiu W, Tang J, et al. Efficacy of electroacupuncture in patients with failed back surgery syndrome: study protocol for a randomized controlled trial. Trials. 2021;22(1):702.
72. Yan B, Wang FC, Ma TS, Liu YZ, Liu W, Cheng L, et al. Efficacy and safety of electroacupuncture treatment in the prevention of negative moods in healthy young men after 30 h of total sleep deprivation: study protocol for a single-center, single-blind, parallel-arm, randomized clinical trial. Trials. 2021;22(1):761.
73. Xu H, Hao M, Zheng C, Yang H, Yin Y, Yang L, et al. Effect of acupuncture for diminished ovarian reserve: study protocol for a randomized controlled trial. Trials. 2021;22(1):720.
74. Yu B, Xuan L, Jin Y, Chen S, Liu S, Wan Y. Efficacy and safety of thread embedding acupuncture for facial expression muscles atrophy after peripheral facial paralysis: study protocol for a randomized controlled trial. Trials. 2021;22(1):755.
75. Chen Y, Bian Y, Li S, Zhao Y, Li J, Zheng Y, et al. Effect of acupuncture at Zusanli (ST36) point on antral contraction function under ultrasound guidance: study protocol of a randomized controlled trial. Trials. 2021;22(1):803.
76. Yu FT, Long DH, Shi GX, Wang LQ, Tu JF, Gang LL, et al. Evaluating the efficacy and safety of electro-acupuncture in patients with antipsychotic-related constipation: protocol for a randomized controlled trial. Trials. 2021;22(1):771.
77. Liu X, Xie X, Li Y, Li M, Wang Y, Wang N, et al. Efficacy of manual acupuncture versus placebo acupuncture for generalized anxiety disorder (GAD) in perimenopause women: study protocol for a randomized controlled trial. Trials. 2021;22(1):833.

78. Li M, Yao L, Huang H, Zhang L, Zheng H, Wang G, et al. Multimodal cerebral imaging study on the effects of "Adjust Zang Dredge Meridian" electroacupuncture on cerebral central sensitization in PDPN patients: a study protocol for a sham-controlled, randomized trial. Trials. 2021;22(1):799.
79. Zhang Y, Guo S, Wang C, Liu X, Liu Y, Shang H, et al. Acupuncture for prostatectomy incontinence: study protocol for a multicenter single-blind randomized parallel controlled trial. Trials. 2022;23(1):9.
80. Yao L, Li M, Sun S, Xu M, Yu S, Zhang Z, et al. Multimodal brain imaging effect of "Adjust Zang-fu and Arouse Spirit" electroacupuncture on diabetic cognitive impairment: study protocol of a randomized, sham-controlled pilot trial. Trials. 2021;22(1):847.
81. Yang L, Zhang H, Zhou L, Gao Y, Yang L, Hu Y, et al. Effect of electro-acupuncture on ovarian function of women with diminished ovarian reserve: study protocol for a randomized controlled trial. Trials. 2021;22(1):921.
82. Jiang T, Zhang Q, Yuan F, Zhang F, Guo J. Efficacy of acupuncture and its influence on the emotional network in adult insomnia patients: protocol for a randomized controlled clinical trial. Trials. 2022;23(1):11.
83. Liu X, Wang Z, Yao H, Yang Y, Cao H, Toh Z, et al. Effects of acupuncture treatment on postoperative gastrointestinal dysfunction in colorectal cancer: study protocol for randomized controlled trials. Trials. 2022;23(1):100.
84. Chen L, Chen Y, Wu L, Fu W, Wu L, Fu W. Efficacy of acupuncture on cognitive function in poststroke depression: study protocol for a randomized, placebo-controlled trial. Trials. 2022;23(1):85.
85. Wang L, Wang R, Yao Y, Bai X, Sheng G. The effects of acupuncture on psychological symptoms in patients with insomnia: study protocol for a randomized controlled trial. Trials. 2022;23(1):152.
86. Chen KB, Wu ZW, Wang J, Zhu LH, Jin XL, Chen GF, et al. Efficacy and safety of long-term transcutaneous electroacupuncture versus sham transcutaneous electroacupuncture for delayed gastric emptying after distal gastrectomy: study protocol for a randomized, patient-assessor blinded, controlled trial. Trials. 2022;23(1):189.
87. Ren H, Zeng Y, Zhang M, Zhang S, Chen Z, Wu B, et al. Electro-acupuncture for protracted amphetamine abstinence syndrome: study protocol for a pragmatic randomized controlled trial. Trials. 2022;23(1):216.
88. Yu B, Hazlewood PJ, Yin X, Li S, Yue H, Xu K, et al. Effect of electroacupuncture on discomfort during gastroscopy: a study protocol for a randomized controlled trial. Trials. 2022;23(1):364.
89. Shen H, Han Y, Wu D, Hu L, Ma Y, Wu F, et al. Trial of transcutaneous electrical acupoint stimulation in laryngopharyngeal reflux disease: study protocol for a randomized controlled trial. Trials. 2022;23(1):272.
90. Peron R, Rampazo É P, Liebano RE. Traditional acupuncture and laser acupuncture in chronic nonspecific neck pain: study protocol for a randomized controlled trial. Trials. 2022;23(1):408.
91. Chen J, Zhou S, Sun M, Wang Y, Chen X, Guo T, et al. Manual acupuncture as prophylaxis for migraine without aura: study protocol for a multi-center, randomized, single-blinded trial. Trials. 2022;23(1):574.
92. Qi LY, Yang JW, Yan SY, She YF, Hu H, Li Y, et al. Effect of acupuncture for diarrhea-predominant irritable bowel syndrome: study protocol for a randomized clinical trial. Trials. 2022;23(1):711.
93. Chang Y, Wu N, Zhang Z, Zhang Z, Ren B, Liu F, et al. Efficacy of manual acupuncture, electro-acupuncture, and warm acupuncture for knee osteoarthritis: study protocol for a randomized controlled trial. Trials. 2022;23(1):700.
94. Agrawal S, Szmit M, Wełna M, Rudnicki J, Agrawal A, Goździk W. Transcutaneous electrical acupoint stimulation to reduce opioid consumption in patients undergoing inguinal hernia repair: protocol for a randomized controlled trial. Trials. 2022;23(1):1064.
95. Liu Q, Wu X, Guo J, Gao J, Liu B, Wang Y, et al. Analgesic Effect of Electroacupuncture on Postherpetic Neuralgia: a Trial Protocol for a Multicenter Randomized Controlled Trial. Pain and therapy. 2021;10(2):1755.
96. Zeng R, Lai F, Huang M, Zhu D, Chen B, Tao L, et al. Feasibility of electroacupuncture at Baihui (GV20) and Zusanli (ST36) on survival with a favorable neurological outcome in patients with postcardiac arrest syndrome after in-hospital cardiac arrest: study protocol for a pilot randomized controlled trial. Pilot Feasibility Stud. 2023;9(1):8.
97. Hou WG, Pan HT, Wang RP, Feng CC, Pei LJ, Li J, et al. "Fan-zhen Jie-ci" acupuncture therapy for treatment of discogenic sciatica: protocol for a single-blind, randomized controlled clinical trial. Ann Palliat Med. 2021;10(1):733-41.
98. Guan LX, Song XL, Wang X, Zhang X, Liu LM, Chen BL, et al. Immediate effects of Zhongji point acupuncture on

pelvic floor structure in female patients with stress urinary incontinence: a randomized, single-blind, and sham-controlled clinical trial protocol. Ann Palliat Med. 2021;10(7):8292-9.
99. Qu M, Gao X, Jia X, Gao Y, Cao W, Li J, et al. Efficacy of electro-acupuncture for gastrointestinal motility after colorectal cancer surgery: study protocol for a randomized controlled trial. 2020.
100. Liu R, Yu X, Wang J, Liu Y, Liu B, Li X, et al. Acupuncture Collaborative Care for Patients with Poststroke Cognitive Impairment: Protocol for a Randomized Controlled Trial. 2020.
101. Tang H, Lu Y, Ren H, Fu J, Meng Y, Li B, et al. Efficacy and Safety of Deep Acupuncture at Qugu Point (CV2) for Benign Prostatic Hyperplasia: Study Protocol for a Randomized Controlled Trial. 2021.
102. Bai Y, Wang Y, Chen B, Qin Y, Lei Q, Zhao H, et al. Stuck-moving needle acupuncture myofascial trigger point to treat idiopathic frozen shoulder: study protocol for a randomized controlled trial. Trials. 2020;21(1):901.
103. Wang J, Li D, Tang W, Guo J, Chen W, Yong Y, et al. Pretreatment with transcutaneous electrical acupoint stimulation to prevent postoperative ileus in patients undergoing laparoscopic colon surgery: study protocol for a randomised controlled trial. BMJ Open. 2020;10(8):e030694.
104. Wang C, Yang WJ, Yu XT, Fu C, Li JJ, Wang J, et al. Acupuncture for insomnia with short sleep duration: protocol for a randomised controlled trial. BMJ Open. 2020;10(3):e033731.
105. Lim JH, Kim KO, Kim SH, Kang CW, Kim BK. Electroacupuncture for treatment-resistant insomnia: study protocol for a randomised, controlled, assessor-blinded, pilot clinical trial. BMJ Open. 2020;10(2):e034239.
106. Jiang L, Geng H, Lu M, Du Z, Chen P, Han X, et al. Acupuncture for poststroke hemiplegia focusing on cerebral bilateral connections: study protocol for a randomised controlled neuroimaging trial. BMJ Open. 2020;10(4):e034548.
107. Huang Z, Zhao J, Pei X, Wang B. Effectiveness of deep electroacupuncture with strong deqi and shallow electroacupuncture with no deqi for lumbar disk herniation: study protocol for a randomised controlled trial. BMJ Open. 2020;10(11):e036528.
108. Wang W, Liu S, Liu Y, Zang Z, Zhang W, Li L, et al. Efficacy of acupuncture versus sham acupuncture or waitlist control for patients with chronic plantar fasciitis: study protocol for a two-centre randomised controlled trial. BMJ Open. 2020;10(9):e036773.
109. Sun Y, Liu Y, Chen H, Yan Y, Liu Z. Electroacupuncture for stress-predominant mixed urinary incontinence: a protocol for a three-armed randomised controlled trial. BMJ Open. 2021;11(1):e038452.
110. Zheng Q, Zheng H, Zhou S, Shi Y, Zhang L, Xiao X, et al. Efficacy of acupuncture treatment for chronic spontaneous urticaria: study protocol for a randomised controlled trial. BMJ Open. 2022;12(2):e045027.
111. Zhao J, Chen M, Wang X, Ye K, Shi S, Li H, et al. Efficacy of acupuncture in refractory irritable bowel syndrome: study protocol for a randomised controlled trial. BMJ Open. 2021;11(9):e045655.
112. Huang H, Wang J, Li H, Lei R, Zou W, Huang Q, et al. Acupuncture for retinitis pigmentosa: study protocol for a randomised, sham-controlled trial. BMJ Open. 2021;11(11):e049245.
113. Zou X, Yang YC, Wang Y, Pei W, Han JG, Lu Y, et al. Electroacupuncture versus sham electroacupuncture in the treatment of postoperative ileus after laparoscopic surgery for colorectal cancer: study protocol for a multicentre, randomised, sham-controlled trial. BMJ Open. 2022;12(4):e050000.
114. Sun N, He DM, Ye X, Bin L, Zhou Y, Deng X, et al. Immediate acupuncture with GB34 for biliary colic: protocol for a randomised controlled neuroimaging trial. BMJ Open. 2022;12(1):e050413.
115. Chen H, Liu X, Yan Y, Shi H, Liu Z. Effect of electroacupuncture on symptoms of female pelvic organ prolapse (stage II-III) (EAPOP study): protocol of a randomised controlled trial. BMJ Open. 2022;12(6):e051249.
116. Li H, Wen Q, Lu L, Hu H, He Y, Zhou Y, et al. Transcutaneous electrical acupoint stimulation combined with electroacupuncture for rapid recovery of patients after laparotomy for gastrointestinal surgery: a study protocol for a randomised controlled trial. BMJ Open. 2021;11(11):e053309.
117. Yu FT, Liu CZ, Ni GX, Cai GW, Liu ZS, Zhou XQ, et al. Acupuncture for chronic sciatica: protocol for a multicenter randomised controlled trial. BMJ Open. 2022;12(5):e054566.
118. Romero SAD, Emard N, Baser RE, Panageas K, MacLeod J, Walker D, et al. Acupuncture versus massage for pain in

patients living with advanced cancer: a protocol for the IMPACT randomised clinical trial. BMJ Open. 2022;12(9):e058281.

119. Zhang Y, Yang G, Wei J, Chen F, Zhang MZ, Mao S. Prospective comparison of acupuncture with sham acupuncture to determine impact on sedation and analgesia in mechanically ventilated critically ill patients (PASSION study): protocol for a randomised controlled trial. BMJ Open. 2022;12(8):e059741.
120. Li Q, Feng J, Zhang X, Wang Y, Zhao S, Xing C, et al. Efficacy of contralateral acupuncture in women with migraine without aura: protocol for a randomised controlled trial. BMJ Open. 2022;12(6):e061287.
121. Eucker SA, Glass O, Staton CA, Knisely MR, O'Regan A, De Larco C, et al. Acupuncture for acute musculoskeletal pain management in the emergency department and continuity clinic: a protocol for an adaptive pragmatic randomised controlled trial. BMJ Open. 2022;12(9):e061661.
122. Wu XD, Chen TY, Wang K, Wei XQ, Feng JJ, Zhou J. Efficacy and safety of transcutaneous electrical acupoints stimulation for preoperative anxiety in thoracoscopic surgery: study protocol for a randomised controlled trial. Bmj Open. 2023;13(2).
123. Xu D, Yu Z, Cai X, Lin J, Lin T, Sun J, et al. Effect of Fu's subcutaneous needling for cancer pain management: protocol for a pragmatic randomised controlled trial. BMJ Open. 2023;13(4):e068232.
124. Yin S, Chang Y, Yan X, Zhang Z, Yan X, Zhang Z, et al. Effect of Acupuncture for Patients with Knee Osteoarthritis: Study Protocol for a Double-dummy Randomized Controlled Trial. 2023.
125. Lv Z, Gu YM, Liu RD, Su KQ, Ruan XD, Chang YN, et al. The Clinical Observation and Mechanism of Acupuncture on Cancer-Related Fatigue of Breast Cancer Based on "Gut-Brain Axis": Study Protocol for a Randomized Controlled Trial. Dis Markers. 2022;2022:8099595.
126. Li Q, Lu Y, Zhang X, Chen Z, Feng J, Zeng X, et al. Brain-Imaging Mechanisms on Female Abdominal Obesity Treated by "Shu-Mu" Acupoint Catgut Embedding and Compatibility Relation: Study Protocol for a 12-Week Randomized Controlled Trial. Diabetes Metab Syndr Obes. 2023;16:733-47.
127. Lin R, Huang J, Xu J, Tao J, Xu Y, Liu J, et al. Effect and Neuroimaging Mechanism of Electroacupuncture for Vascular Cognitive Impairment No Dementia: study Protocol for a Randomized, Assessor-Blind, Controlled Clinical Trial. Evidence-based complementary and alternative medicine. 2020;2020.
128. Li J, Su K, Mei J, Wang Y, Yin S, Hu Y, et al. Using Surface Electromyography to Evaluate the Efficacy of Governor Vessel Electroacupuncture in Poststroke Lower Limb Spasticity: study Protocol for a Randomized Controlled Parallel Trial. Evidence-based complementary and alternative medicine. 2021;2021.
129. Kim J, Kwon SK, Lee IS, Yeom M, Hahm DH, Park HJ, et al. Effect of Acupuncture on Gut-Brain Axis Parameters in Patients with Atopic Dermatitis: a Study Protocol for a Randomized, Participant- And Assessor-Blind, Sham-Controlled Trial. Evidence-based complementary and alternative medicine. 2021;2021.
130. Wang YN, Sun MS, Ni XX, Tian T, Liu L, Li X, et al. Comparison of Effects and Brain-Gut Regulatory Mechanisms of Acupuncture and Flunarizine for Migraine: study Protocol for a Randomized Controlled Trial. Evidence-based complementary and alternative medicine. 2021;2021.
131. Zhao N, Zhang H, Liu T, Liu J, Xiang Y, Shu G, et al. Neuromodulatory Effect of Sensorimotor Network Functional Connectivity of Temporal Three-Needle Therapy for Ischemic Stroke Patients with Motor Dysfunction: study Protocol for a Randomized, Patient-Assessor Blind, Controlled, Neuroimaging Trial. Evidence-based complementary and alternative medicine. 2021;2021.
132. Li X, Gao Y, Zhang C, Zhang Q, Xin X, Tan Z, et al. Evaluating the Long-Term Efficacy of Acupuncture Therapy for Subacute Poststroke Aphasia: study Protocol for a Randomized, Blinded, Controlled, Multicentre Trial. Evidence-based complementary and alternative medicine. 2021;2021.
133. Fan JQ, Xu ZQ, Chen YY, Lu WJ, Xie XY, Wang YT, et al. Efficacy of Acupuncture for Parkinson's Disease Anxiety: two-Stage Protocol for a Randomized Controlled Clinical Trial. Evidence-based complementary and alternative medicine. 2022;2022.
134. Fan L, Lu G, Yuan H, Wang X, Qiao H. Effects of acupuncture at acupoints along lung meridian on cervical spondylosis of cervical type: A protocol for randomized controlled trial. Medicine: Case Reports and Study

Protocols. 2021;2(7):e0125.
135. Guo Y, Ho LF, Chen L, Tsang PH, Chen M, Chan KL, et al. Electroacupuncture with or without combined warm needling for tinnitus: Study protocol for a randomized, waitlist-controlled trial. Medicine. 2023;102(29):e34315.
136. Zhang P, Chen Y, Zhang F, Pei H, Sun M, Qu Y, et al. Effect of Acupuncture on Blood Pressure and Metabolic Profile Among Patients With Essential Hypertension: Protocol of a Randomized Clinical Trial. Front Cardiovasc Med. 2022;9:888569.
137. Hu H, Shen Y, Li X, Tian H, Li X, Li Y, et al. Efficacy of Electroacupuncture Therapy in Patients With Postherpetic Neuralgia: Study Protocol for a Multicentre, Randomized, Controlled, Assessor-Blinded Trial. Front Med (Lausanne). 2021;8:624797.
138. Wang W, Wang X, Liu Y, Sun Y, Liu X, Yan Y, et al. Effects of Electroacupuncture on Opioid-Induced Constipation in Patients With Cancer: Study Protocol for a Multicenter Randomized Controlled Trial. Front Med (Lausanne). 2022;9:818258.
139. Wang W, Chen H, Gao N, Yu S, Liao J, Wang S, et al. Effect of acupuncture at the sphenopalatine ganglion for the treatment of moderate to severe seasonal allergic rhinitis: Study protocol for a three-armed randomized controlled trial. Front Med (Lausanne). 2022;9:904864.
140. Yao J, Yan X, Chen L, Li Y, Zhang L, Chen M, et al. Efficacy and MicroRNA-Gut Microbiota Regulatory Mechanisms of Acupuncture for Severe Chronic Constipation: Study Protocol for a Randomized Controlled Trial. Front Med (Lausanne). 2022;9:906403.
141. Yan Y, Sun Y, Wang X, Zhu L, Chen Y, Liu Z. Acupuncture for Impaired Glucose Tolerance in People With Obesity: A Protocol for a Multicenter Randomized Controlled Trial. Front Med (Lausanne). 2022;9:932102.
142. Kong X, Ma Z, Tang R, Wang X, Wei K, Yang G, et al. Efficacy of acupuncture in patients with mild Alzheimer's disease and its impact on gut microbiota: Study protocol for a randomized sham-controlled trial. Front Med (Lausanne). 2023;10:1014113.
143. Kim JH, Yang C, Yoo J, Park GC, Kang BK, Kim AR, et al. Safety and efficacy of 650 nm invasive laser acupuncture on non-specific chronic low back pain: A protocol for a multicenter randomized placebo-controlled trial. Front Med (Lausanne). 2023;10:1021255.
144. Bao Q, Liu Y, Zhang X, Li Y, Wang Z, Ye F, et al. Clinical observation and mechanism of acupuncture on amnestic mild cognitive impairment based on the gut-brain axis: study protocol for a randomized controlled trial. Front Med (Lausanne). 2023;10:1198579.
145. Hu T, Hu H, Chen F, Jiang B, Shen F, Su Y, et al. The Efficacy and Safety of Acupuncture for Prophylaxis of Vestibular Migraine: A Study Protocol for a Randomized Controlled Trial. Front Neurol. 2021;12:709803.
146. Zhou J, Jiang NN, Fang Y, Zhang XY, Cheng SR, Li XL, et al. Efficacy of Acupuncture Treatment of Migraine Delivered by Senior or Junior Acupuncturists: Study Protocol for a Randomized Controlled Trial. Front Neurol. 2021;12:812504.
147. Mei J, Xue Y, Li J, Zhang L, Zhang J, Wang Y, et al. Effects of Functional Acupuncture on Upper Limb Spasticity After Ischemic Stroke: A Protocol for a Randomized Controlled Parallel Clinical Trial. Front Neurol. 2022;13:835408.
148. Lu C, Bao W, Deng D, Li R, Li G, Zou S, et al. Efficacy of electroacupuncture with different frequencies in the treatment of chemotherapy-induced peripheral neuropathy: A study protocol for a randomized controlled trial. Front Neurol. 2022;13:843886.
149. Yang LS, Li YM, Zhou DF, Zhao BM, Zheng SZ, Chen ZH, et al. Effects of Qihuang Needling on Motor Function for Patients With Parkinson's Disease: Study Protocol for a Multicenter, Randomized Controlled Trial. Front Neurol. 2022;13:902170.
150. Li H, Wang M, Wu Y, Chen X, Xue C, Liu P, et al. Clinical Effect of Electroacupuncture on Acute Sleep Deprivation and Event-Related Potential Affecting the Inhibition Control of the Brain: Study Protocol for a Randomized Controlled Trial. Front Neurol. 2022;13:911668.
151. Hong J, Sun J, Zhang L, Tan Z, Chen Y, Chen Q, et al. Neurological mechanism and treatment effects prediction of acupuncture on migraine without aura: Study protocol for a randomized controlled trial. Front Neurol.

2022;13:981752.
152. Lu C, Li G, Deng D, Li R, Li X, Feng X, et al. Efficacy of electroacupuncture in the treatment of peripheral neuropathy caused by Utidelone: Study protocol for a randomized controlled trial. Front Neurol. 2023;14:1065635.
153. Li X, Yin X, Feng H, Liao W, Zhao J, Su W, et al. Acupoint catgut embedding for chronic non-specific low back pain: A protocol of randomized controlled trial. Front Neurosci. 2023;17:1106051.
154. Xian J, Wang L, Sun M, Wang X, Zang XM, Yu HJ, et al. Acupuncture for Subthreshold Depression: Study Protocol for a Randomized Controlled Trial. Frontiers in Psychiatry. 2022;12.
155. Yang S, Qin Z, Yang X, Chan MY, Zhang S, Rong P, et al. Transcutaneous Electrical Cranial-Auricular Acupoint Stimulation vs. Escitalopram for Patients With Mild-to-Moderate Depression (TECAS): Study Design for a Randomized Controlled, Non-inferiority Trial. Front Psychiatry. 2022;13:829932.
156. Li M, Wang Y, Li K, Xu X, Zhuang L. The efficacy and safety of Jin's three-needle therapy vs. placebo acupuncture on anxiety symptoms in patients with post-stroke anxiety: A study protocol for a randomized controlled trial. Front Psychiatry. 2022;13:941566.
157. Wang X, Wang P, Liu C, Qin S, Wan Q, Luo S, et al. Acupuncture for hypertension with insomnia: Study protocol for a randomized, sham-controlled, subject-and-assessor-blinded trial. Front Psychiatry. 2022;13:1087706.
158. Hou Y, Lu J, Xie J, Zhu R, Wu M, Wang K, et al. Effects of electroacupuncture on perioperative anxiety and stress response in patients undergoing surgery for gastric or colorectal cancer: Study protocol for a randomized controlled trial. Front Psychiatry. 2023;14:1095650.
159. Wu X, Tu M, Chen N, Yang J, Jin J, Qu S, et al. The efficacy and cerebral mechanism of intradermal acupuncture for major depressive disorder: a study protocol for a randomized controlled trial. Front Psychiatry. 2023;14:1181947.
160. Tu M, Wu X, Qu S, Jin J, Chen N, Xiong S, et al. The effective on intradermal acupuncture based on changes in biological specificity of acupoints for major depressive disorder: study protocol of a prospective, multicenter, randomized, controlled trial. Front Psychiatry. 2023;14:1183127.
161. Liu Y, Zhu Y, Jiang L, Lu C, Xiao L, Chen J, et al. Efficacy of Acupuncture in Post-partum With Diastasis Recti Abdominis: A Randomized Controlled Clinical Trial Study Protocol. Front Public Health. 2021;9:722572.
162. Li H, Du C, Lu L, Hu X, Xu H, Li N, et al. Transcutaneous electrical acupoint stimulation combined with electroacupuncture promotes rapid recovery after abdominal surgery: Study protocol for a randomized controlled trial. Front Public Health. 2022;10:1017375.
163. de Oliveira NM, Machado J, Huang Z, Criado MB. Acupuncture in Women with Human Polycystic Ovary/Ovarian Syndrome: Protocol for a Randomized Controlled Trial. Healthcare (Basel). 2022;10(10).
164. Correia de Carvalho M, Pereira Machado J, Laranjeira M, Nunes de Azevedo J, Azevedo P. Effect of Acupuncture on Functional Capacity and Health-Related Quality of Life of Hemodialysis Patients: Study Protocol for a Randomized Controlled Trial. Healthcare (Basel). 2022;10(10).
165. Gao S, Sun Y, Shi H, Fang J, Liu Z. Efficacy and Safety of Acupuncture for Cyclic Mastalgia: Study Protocol for a Randomized, Sham-Controlled Trial. Int J Womens Health. 2023;15:845-55.
166. Cardoso RF, Lacerda ACR, Lima VP, de Oliveira LFF, de Oliveira SFF, Araújo RP, et al. Efficacy of Acupuncture on Quality of Life, Functional Performance, Dyspnea, and Pulmonary Function in Patients with Chronic Obstructive Pulmonary Disease: Protocol for a Randomized Clinical Trial. J Clin Med. 2022;11(11).
167. Goo B, Baek YH. Thread-Embedding Acupuncture for the Treatment of Shoulder Instability: Protocol for a Randomized, Controlled, Patient-Assessor Blinded Pilot Study. J Pain Res. 2021;14:2729-37.
168. 168.Tang D, Zhang X, Xu Y, Dai L, Sun J, Hu H, et al. The Central Response of Electroacupuncture on Trigeminal Neuralgia Based on Resting-State Functional Magnetic Resonance Imaging: A Protocol for a Pre-Experimental, Single-Centre, Randomized, Controlled Trial. J Pain Res. 2021;14:3321-31.
169. Sun R, Li S, Ren L, Xia Y, Wang Y, Bian Z, et al. Efficacy of Electroacupuncture for the Treatment of Postherpetic Neuralgia: Study Protocol for a Multicenter Randomized Controlled Trial. J Pain Res. 2022;15:959-68.
170. Yin S, Zhang ZH, Chang YN, Huang J, Wu ML, Li Q, et al. Effect of Acupuncture on the Cognitive Control Network of Patients with Knee Osteoarthritis: Study Protocol for a Randomized Controlled Trial. J Pain Res. 2022;15:1443-

55.
171. Wu P, Zhu L, Zheng SY, Li JX, Wu MD, Wang WJ, et al. Transcutaneous Electrical Acupoint Stimulation for Moderate to Severe Pain in Hepatocellular Carcinoma: A Protocol for a Randomized Controlled Trial. J Pain Res. 2022;15:1889-96.
172. Xu YY, Chen YY, Shi Y, Lu JH, Wu ZL, Liu Z, et al. Electro-Acupuncture for Bladder Pain Syndrome: A Protocol of a Randomized Controlled Trial and for Central Mechanism. Journal of Pain Research. 2022;15:1959-70.
173. Zhou CL, Bao J, Hu HT, Ye ST, Shao XM, Liang Y, et al. Acupuncture Based on Regulating Autonomic Nerves for the Prevention of Migraine Without Aura: A Prospective, Double-Dummy, Randomized Controlled Clinical Trial. Journal of Pain Research. 2022;15:2211-21.
174. Kim JH, Kang D, Kim KW, Nam SS, Goo B. Thread Embedding Acupuncture for Temporomandibular Disorder: Protocol for a Pilot Randomized Controlled Trial. J Pain Res. 2022;15:3197-207.
175. Jia J, Yan C, Zheng X, Shi A, Li Z, Xu L, et al. Central Mechanism of Acupuncture Treatment in Patients with Migraine: Study Protocol for Randomized Controlled Neuroimaging Trial. J Pain Res. 2023;16:129-40.
176. Goo B, Park YC, Kim E, Sung WS, Kim EJ, Kim JH, et al. Efficacy, Safety and Cost-Effectiveness of Thread-Embedding Acupuncture for Adhesive Capsulitis (Frozen Shoulder): A Study Protocol for a Multicenter, Randomized, Patient-Assessor Blinded, Controlled Trial. J Pain Res. 2023;16:623-33.
177. Wang MA, Xu XB, Zhao BC, Liu L, Zhao LP, Zhang F, et al. Fire Needling Therapy of Different Frequencies versus External Diclofenac Diethylamine Emulgel for Knee Osteoarthritis: Study Protocol for a Pilot Randomized Controlled Trial. Journal of Pain Research. 2023;16:1381-90.
178. Hu H, Cheng Y, Wu L, Han D, Ma R. Investigating the Therapeutic Effect of Intradermal Acupuncture for Acute Herpes Zoster and Assessing the Feasibility of Infrared Thermography for Early Prediction of Postherpetic Neuralgia: Study Protocol for a Randomized, Sham-Controlled, Clinical Trial. J Pain Res. 2023;16:1401-13.
179. Wang M, Yuan F, Xu X, Zhang T, Guo J, Wang G, et al. Fire Needling Therapy versus Manual Acupuncture in Post-Stroke Complex Regional Pain Syndrome of the Upper Limb: Study Protocol for a Pilot Randomised Controlled Trial. J Pain Res. 2023;16:2347-56.
180. Mahasti S, Caroline B, Bernard A, Yunfen LI, Jianyun N, Dequan L, et al. An international multicentric phase Ⅲ randomized controlled trial of time-acupoints-space acupuncture for the prevention of chemotherapy-induced fatigue in patients with early stage breast cancer: a study protocol. J Tradit Chin Med. 2022;42(2):289-95.
181. Ying G, Yao W, Hongchun C, Ran Y, Tao L, Jinhao Z, et al. Efficacy of press needle on immune function and quality of life in female breast cancer patients after radical mastectomy: study protocol for a randomized controlled trial. J Tradit Chin Med. 2023;43(2):374-8.
182. Zhong LL, Wong Y, Leung C, Choy C, Cho H, Wong AY, et al. Acupuncture for olfactory dysfunction in infected COVID-19 patients: study protocol for a randomized, sham-controlled clinical trial. Journal of Traditional Chinese Medical Sciences. 2022.
183. Psutka SP, Veleber S, Siman J, Jannat S, Holt S, Wright JL, et al. Effects of acupuncture to decrease adverse events in patients with high-risk non-muscle invasive bladder cancer receiving induction intravesical BCG therapy: Study protocol for a randomized, controlled pilot and feasibility study. Contemp Clin Trials Commun. 2022;30:101044.
184. Lee JH, Cho TJ, Park MG, Kim JH, Song SK, Park SY, et al. Clinical study on concurrent use of electro-acupuncture or Chuna manual therapy with pregabalin for chemotherapy-induced peripheral neuropathy: safety and effectiveness (open-labeled, parallel, randomized controlled trial, assessor-blinded): a study protocol. Medicine. 2020;99(3):e18830.
185. Yang J, Yang C, Wang Y, Li N, Yao X, Yang B, et al. Effect of subcutaneous needling on visual analogue scale, IgG and IgM in patients with lumbar disc herniation: study protocol clinical trial (SPIRIT Compliant). Medicine. 2020;99(9):e19280.
186. Huang Y, Qi S, Wu X, Zhi N, He T, Shen M, et al. Randomized controlled trial for the efficacy of electroacupuncture in the treatment of urge urinary incontinence: a clinical study protocol. Medicine. 2020;99(9):e19315.
187. Wu SY, Lin CH, Chang NJ, Hu WL, Hung YC, Tsao Y, et al. Combined effect of laser acupuncture and

electroacupuncture in knee osteoarthritis patients: a protocol for a randomized controlled trial. Medicine. 2020;99(12):e19541.
188. Liu L, Yuan X, Yang L, Zhang J, Luo J, Huang G, et al. Effect of acupuncture on hormone level in patients with gastrointestinal dysfunction after general anesthesia: a study protocol for a randomized controlled trial. Medicine. 2020;99(14):e19610.
189. Sun J, Li R, Li X, Chen L, Liang Y, Zhang Q, et al. Electroacupuncture therapy for change of pain in classical trigeminal neuralgia. Medicine (Baltimore). 2020;99(16):e19710.
190. Li Y, Yu X, Liu R, Wang J, Deng S, Liu B, et al. Acupuncture for erectile dysfunction in post-stroke patients: study Protocol Clinical Trial (SPIRIT Compliant). Medicine. 2020;99(15):e19718.
191. Chen Q, Tao Y, Wang L, Zhang J, Sun B, Yang X. A randomized controlled clinical study of acupuncture therapy for Seborrheic alopecia in young and middle ages: study protocol clinical trial (SPIRIT compliant). Medicine. 2020;99(17):e19842.
192. Han X, Gao Y, Wang S, Chen Q. Effect of electroacupuncture on diabetic neurogenic bladder: a randomized controlled trial protocol. Medicine. 2020;99(17):e19843.
193. Chen G, Wang X, Zhang S, Xu X, Liang J, Xu Y. In vivo investigation on bio-markers of perimenopausal panic disorder and catgut embedding acupoints mechanism. Medicine (Baltimore). 2020;99(19):e19909.
194. Zheng MQ, Weng C, Hu W, Shen CQ, Tao Y, Pan ZW. Efficacy assessment of acupuncture in improving symptoms of uterine fibroids: a randomized controlled trial. Medicine. 2020;99(18):e20016.
195. Liou KT, Baser R, Romero SAD, Green J, Li QS, Orlow I, et al. Personalized electro-acupuncture versus auricular-acupuncture comparative effectiveness (PEACE): a protocol of a randomized controlled trial for chronic musculoskeletal pain in cancer survivors. Medicine. 2020;99(21):e20085.
196. Shao Y, Wang P, Wang Q, Yu L, Zhang L, Wang W. Eye-acupuncture with rehabilitation therapy for stroke. Medicine (Baltimore). 2020;99(18):e20096.
197. Li F, Qi Z, Hua L, Wang X, Ling M, Juan D. The efficacy of acupuncture for the treatment and the fertility improvement in child-bearing period female with Hashimoto Disease: a randomized controlled study. Medicine. 2020;99(27):e20909.
198. Yuan J, Wang H, Chen J, Lei Y, Wan Z, Zhao Y, et al. Effect of low frequency repetitive magnetic stimulation at Shenmen (HT7) on sleep quality in patients with chronic insomnia. Medicine (Baltimore). 2020;99(30):e21292.
199. Chen Y, Song H, Chen M, Xu H. The role of acupotomy in treatment of patients with lumbar spinal stenosis: a protocol for a randomized study. Medicine. 2020;99(31):e21444.
200. Ning Y, Liu X, Yao H, Chen P, Li X, Jia H. The fMRI study for acupuncture on shift work sleep disorder: study protocol for a randomized controlled neuroimaging trial. Medicine. 2020;99(36):e22068.
201. Cai W, Ma W, Chen AW, Shen WD. Effects of electroacupuncture therapy for depression: study protocol for a multicentered, randomized controlled trial. Medicine. 2020;99(38):e22380.
202. Wu N, Huang J, Yang X, Guo J, Liu F, Gu Y, et al. The long-term effect of acupuncture for patients with knee osteoarthritis: study protocol for a randomized controlled trial. Medicine. 2020;99(42):e22599.
203. Li W, Wang H, Wang L, Tang P, Huang Y. Acupoint injection versus sacral canal injection in lumbar disc herniation: a protocol of randomized controlled trial. Medicine. 2020;99(46):e23000.
204. Du YZ, Zhang LL, Liu W, Rao C, Li BX, Nan X, et al. Effect of acupuncture treatment on post-stroke cognitive impairment A randomized controlled trial. Medicine. 2020;99(51).
205. Li D, Jiang Y, Ma X, Li Q, Chu X, Zhong W, et al. The effect of pestle acupuncture for patients with lactation insufficiency after cesarean section: study protocol for a randomized controlled trial. Medicine. 2021;100(3):e23808.
206. Liu MY, Sung L, Liao YS, Jiao YF, Sun CS, Peng XD. The efficacy and safety of auricular acupuncture versus electroacupuncture in ameliorating chemotherapy-induced nausea and vomiting among patients receiving cisplatin-based regimens. Medicine (Baltimore). 2021;100(7):e24588.
207. Ding X, Huang S, Tang Y, Lin J. Effectiveness and safety of ear acupuncture for allergic rhinitis: a protocol of

randomized controlled trial. Medicine. 2021;100(12):e24943.

208. Zhou Y, Shi J, Zhang Y, Zhang X, Dai A, Feng S, et al. Study for cerebral central network mechanism of acupuncture stimulation quantity based on changes of cerebral functional connection of fMRI. Medicine (Baltimore). 2021;100(14):e25480.
209. Yu J, Wang P, Nie C, Zheng B. The efficacy and safety of silver needle in the treatment of rheumatoid arthritis: a protocol of randomized controlled trial. Medicine. 2021;100(18):e25556.
210. Zhou Y, Wang W, Tian K, Huang H, Jia M. Efficacy and safety of electroacupuncture in treatment of cervical spondylosis: a protocol of randomized controlled trial. Medicine. 2021;100(18):e25570.
211. Kao PY, Ben-Arie E, Lu TY, Ho WC, Lee YC, Lin YS, et al. Acupuncture for blunt chest trauma: a protocol for a double-blind randomized control trial. Medicine. 2021;100(18):e25667.
212. Wu J, Zhang X, Zhao J, Xue Y, Yu P, Wu X, et al. Clinical study on acupuncture treatment of hypertension with hyperactivity of liver yang. Medicine (Baltimore). 2021;100(17):e25668.
213. Tang Q, Liang B, Liang R, Zhang S, Zhu L. Study on optimization and evaluation system of traditional Chinese medicine rehabilitation program for swallowing disorder after stroke. Medicine (Baltimore). 2021;100(19):e25731.
214. Pei X, Song S, Li H, Lu D. Efficacy and safety of acupoint catgut embedding in treating postoperative pain of mixed hemorrhoids: a randomized controlled trial protocol. Medicine. 2021;100(19):e25948.
215. Du J, Tao J, Xu M, Wang R, Lin L, Huang X, et al. The effects of acupuncture for patients with psoriasis: study protocol for a randomized controlled trial. Medicine. 2021;100(21):e26042.
216. Li G, Zhang C, Wang C, Xiao L. Acupuncture against chronic postsurgical pain in non-small cell lung cancer patients: a protocol of randomized controlled trial. Medicine. 2021;100(40):e27461.
217. 217.Duan PP, Yan CQ, Feng HS, Chen Y, Sun N, Yao YQ, et al. Clinical study on acupuncture treatment of gastrointestinal damp-heat acne. Medicine (Baltimore). 2021;100(44):e27503.
218. 218.Zhang J, Kuang X, Tang C, Xu N, Xiao S, Xiao L, et al. Acupuncture for amnestic mild cognitive impairment (aMCI): study protocol for a pilot multicenter, randomized, parallel controlled trial. 2021.
219. Zhu L, Tang Q, Zhang L, Xin G, Liang B, Fan Y, et al. Based on voxel-based morphological analysis to investigate the effect of acupuncture-rehabilitation therapy on hippocampal volume and its neuroprotective mechanism in patients with vascular cognitive impairment with type 2 diabetes mellitus: A study protocol. Medicine. 2021;100(51):e28187.
220. Liu C-T, Hsieh T-M, Shih F-Y, Lai W-H, Hsieh C-H, Wu B-Y, et al. The effects of electroacupuncture and laser acupuncture therapy for patients with major trauma: A study protocol. Medicine. 2021;100(52):e28367.
221. Liu Y, Zhang L, Wang S, Long L, Zang Q, Jia G. Efficacy and safety of electroacupuncture at auricular concha region in promoting of rehabilitation of ischemic stroke patients with upper limb motor dysfunction: a study protocol for a randomized pilot trial. Medicine. 2022;101(15):e28047.
222. Zhu X, Yan L, Dou X, Zheng Y, He G, Liao M, et al. Acupuncture treatment of hypertension with insomnia: a protocol for randomized, double-blind, placebo controlled trial research. Medicine. 2022;101(2):e28455.
223. Park KS, Gang WJ, Kim PW, Yang C, Jun P, Jung SY, et al. Efficacy and safety of acupuncture on oligomenorrhea due to polycystic ovary syndrome An international multicenter, pilot randomized controlled trial. Medicine. 2022;101(7).
224. Mayer PK, Kao PY, Lee YC, Liao YF, Ho WC, Ben-Arie E. Acupuncture effect on dumping syndrome in esophagus cancer patients with feeding jejunostomy: a study protocol for a single blind randomized control trial. Medicine. 2023;102(23):e33895.
225. Usichenko TI, Müller-Kozarez I, Knigge S, Busch R, Busch M. Acupuncture for Relief of Gag Reflex in Patients Undergoing Transoesophageal Echocardiography—A Protocol for a Randomized Placebo-Controlled Trial. Medicines. 2020;7(4):17.
226. Mackey S, Gilam G, Darnall B, Goldin P, Kong JT, Law C, et al. Mindfulness-Based Stress Reduction, Cognitive Behavioral Therapy, and Acupuncture in Chronic Low Back Pain: Protocol for Two Linked Randomized Controlled Trials. JMIR Res Protoc. 2022;11(9):e37823.

227. Wang CC, Whitehead L, Cruickshank T, Lo J, Xia J, Wen J. Feasibility and therapeutic efficacy of a twoweek low-level laser acupuncture therapy for shoulder and neck pain in office workers: protocol for a pilot, single-blind, doublearmed, randomised controlled trial. PloS one. 2022;17(1 January).
228. Xue X, Liu X, Pan S, Li J, Wang S, Yuan H, et al. Electroacupuncture treatment of primary dysmenorrhea: a randomized, participantblinded, sham-controlled clinical trial protocol. PloS one. 2023;18(5 May). Origin：ClinicalTrials.gov
229. Romero JdJsP. Evaluation of the effect of electroacupuncture on the sensory symptom of Symmetrical Distal Polyneuropathy of Diabetic origin and its correlation with changes in Nerve Conduction Velocity. 2022. Origin：ClinicalTrials.gov
230. EFFICACY OF PC6 ELECTROACUPUNCTURE IN THE PREVENTION OF NAUSEA VOMITING IN CAESAREAN PATIENT UNDER SPINAL ANAESTHESIA. 2020. Origin：ClinicalTrials.gov
231. Sun J. Analgesic Effect of Electroacupuncture on Postherpetic Neuralgia: a Multicenter Randomized Controlled Trial. 2021. Origin：ClinicalTrials.gov
232. 232.EFFECT OF ACUPUNCTURE ON HEART RATE VARIABILITY IN INDIVIDUALS WITH MULTIPLE SCLEROSIS. 2020. Origin：ClinicalTrials.gov
233. Effectiveness of acupuncture for cyclical mastalgia. 2022. Origin：ClinicalTrials.gov
234. McKee. JDMD. Acupuncture in the Emergency Department for Pain Management: A BraveNet Multi-Center Feasibility Study. 2021. Origin：ClinicalTrials.gov
235. Ha L, Liu X, Liu Y, Zhi M, Jiang H, Zhao J, et al. Scheme optimization of acupoints compatibility and influence factors of the effect. Medicine (Baltimore). 2021;100(50):e27883.
236. Liu R, Wang Z, Zhang J, Ren S, Liu Y, Zhang Z, et al. Effects of Acupuncture for Bell's Palsy in Acute Phase: A Study Protocol for a Randomized Controlled Trial. Available at SSRN 4305772.
237. Wang P, Wang Z, Li Z-x, Ma S-h, Li Y, Li H, et al. Efficacy and safety of Tongdutiaoshen acupuncture on insomnia in maintenance hemodialysis patients: a randomized clinical trial protocol. 2023.
238. Wang Y, Zhou M, Shen J, Wang D, Xu N, Song Y, et al. Effect of electroacupuncture on chemotherapy-induced diarrhea: study protocol for a randomized controlled trial. Transl Cancer Res. 2021;10(5):2516-24.
239. Zheng S, Shen Q, Lyu Z, Tian S, Huang X, Liu Y, et al. Effect of acupuncture or moxibustion at Acupoints Weizhong or Chize on the change in lumbar temperature in healthy adults: a study protocol for a randomized controlled trial with a 2× 2 factorial design. 2023.
240. Wang X, Guo RF, Guo YY, Guo Q, Yan Y, Gong W, et al. Rationale and design of the RESTORE trial: A multicenter, randomized, double-blinded, parallel-group, placebo-controlled trial to evaluate the effect of Shenfu injection on myocardial injury in STEMI patients after PCI. American Heart Journal. 2023;260:9-17.
241. Zhou YS, Mao S, Guo LH, Gao XY, Zou X, Zhang MZ. Effect of Tongguan Capsules ((sic)& x51a0;& x80f6;& x56ca;) on Restenosis after Coronary Stent Implantation: Study Protocol for A Randomized Controlled Trial. Chinese Journal of Integrative Medicine. 2021;27(1):16-23.
242. Liu NY, Pei H, Liu MX, Liu LT, Fu CG, Li H, et al. Efficacy and Safety of Guilingji Capsules () for Treating Mild-to-Moderate Cognitive Impairment: Study Protocol for A Randomized, Double-Blind, Positive-Controlled, Multicenter and Noninferiority Trial. Chin J Integr Med. 2020;26(8):577-82.
243. Wang G, Jia L, Pei Y, Yu R, Gao Y, Deng C, et al. Clinical study for external Chinese herbal medicine LC09 treating hand-foot skin reaction associated with the antitumor targeted drugs: protocol for a prospective, randomized,

controlled, double-blind, and monocentric clinical trial. Medicine. 2020;99(4):e18849.
244. Xu L, Zhang J, Li J, Lv L, Zhang Z, Wang F, et al. Clinical study on post evaluation after listing of Qizhi Weitong granules: study protocol clinical trial (SPIRIT compliant). Medicine. 2020;99(16):e19758.
245. Zhang Y, Yuan H, Kang J, Xie H, Long X, Qi L, et al. Clinical study for external washing by traditional Chinese medicine in the treatment of multiple infectious wounds of diabetic foot: study protocol clinical trial (SPIRIT compliant). Medicine. 2020;99(17):e19841.
246. Zhang S, Liang C, Yang Y, Zhao Z, Li J, Meng X. Effects of Yangxinshi tablet on exercise tolerance in patients with coronary heart disease: a protocol of randomized, double-blind, placebo-controlled, and multi-center trial. Medicine. 2020;99(31):e21485.
247. Lu Y, Chu X, Zhang J, Zhao Y, Jin C, Zhu J, et al. Effect of Shexiang Tongxin dropping pill on stable coronary artery disease patients with normal fractional flow reserve and coronary microvascular disease: a study protocol. Medicine. 2020;99(38):e22126.
248. Liang X, Hu X, Zhang X, Fu H. ASF (a Compound of Traditional Chinese Medicine) in the treatment of patients with alcohol dependence: study protocol of a randomized, double-blinded, placebo-controlled clinical trial. Medicine. 2020;99(52):e23899.
249. Song Z, Sun LY, Gu SS, Zhu XS, Lai HZ, Lu F, et al. Exploring the Safety, Effectiveness, and Cost-Effectiveness of a Chinese Patent Medicine (Fufang E’jiao Syrup) for Alleviating Cancer-Related Fatigue: a Protocol for a Randomized, Double-Blinded, Placebo-Controlled, Multicenter Trial. Integrative cancer therapies. 2021;20.
250. Xiao Z, Hu L, Lin J, Lu L, Huang X, Zhu X, et al. Efficacy and safety of Jianpishengsui for chemotherapy-related fatigue in patients with non-small cell lung cancer: study protocol for a randomized placebo-controlled clinical trial. Trials. 2020;21(1):94.
251. Sha Z, Hou Y, Xue C, Li O, Li Z, Wang H, et al. The efficacy and safety of 'antianxiety granule' for anxiety disorder: a multicentre, randomized, double-blind, placebo-controlled, parallel-group trial. Trials. 2020;21(1):107.
252. Song J, Ma T, Liang Y, Cao X, Sun Z. Efficacy and safety of Dingkun pill for female infertility patients with low prognosis undergoing in vitro fertilization-embryo transfer: study protocol for a multicenter, double-blind, randomized, placebo-controlled trial. Trials. 2020;21(1):550.
253. Yao W, Cheang I, Liao S, Zhou Y, Zhou F, Xu D, et al. Study protocol for a randomized controlled trial: Qiliqiangxin in heart failUre: assESsment of reduction in morTality (QUEST). BMC Complement Med Ther. 2020;20(1):38.
254. Zhang PX, Zeng L, Meng L, Li HL, Zhao HX, Liu DL. Observation on clinical effect of Huoxue-Jiangtang decoction formula granules in treating prediabetes: a randomized prospective placebo-controlled double-blind trial protocol. BMC Complement Med Ther. 2022;22(1):274.
255. Leng Y, Fu X, Qian L, Li Q, Gao H, Xie H, et al. Efficacy, safety and therapeutic mechanism of Shen-Qi Xiao-Tan formula in the treatment of peripheral atherosclerosis in patients with type 2 diabetes mellitus: a randomized, double-blind, placebo-controlled trial protocol. BMC Complement Med Ther. 2022;22(1):337.
256. Huang Q, An ZM, Xin X, Sun QM, Gao ST, Lv S, et al. Effectiveness and safety analysis of Danggui Shaoyao Powder for the treatment of non-alcoholic fatty liver disease: study protocol for a randomized, double-blind, placebo-controlled clinical trial. Bmc Complementary Medicine and Therapies. 2023;23(1).
257. Zheng Y, Ching J, Cheng CW, Lam WC, Chan KL, Zhang X, et al. Efficacy and safety of Chinese medicine JCM-16021 for diarrhea-predominant irritable bowel syndrome: study protocol for a multi-center, randomized, double-blind, placebo controlled clinical trial. Chinese Medicine. 2021;16(1).
258. Hu Y, Gu S, Yuan X, Li H, Yuan C, Ye Q. Traditional Chinese medicine syndrome differentiation and treatment by stages of Parkinson's disease: study protocol for a multicentre, randomized, double-blind, placebo-controlled clinical trial. Chin Med. 2022;17(1):68.
259. Hung HY, Song T, Loo SKF, Chan KL, Ching JYL, Sum CH, et al. Efficacy and safety of modified Xiao-Feng Powder in the treatment of chronic urticaria: protocol of a randomized double-blind placebo-controlled study. Chin Med. 2022;17(1):87.
260. Wang J, Wang Z, Lan T, Zhang L, Li Z, Wang X, et al. Wangbi granule as a combination therapy to achieve clinical

deep remission in rheumatoid arthritis: protocol for a multicenter, triple-blind, randomised, placebo-controlled trial. Chin Med. 2023;18(1):22.
261. Chen P, Zhu H, Ning Y, Yin D, Jia H. Efficacy and safety of Shu-gan-qing-re formula for generalized anxiety disorder: study protocol for a multi-center, prospective, double-blind, double-dummy, randomized controlled trial. Trials. 2020;21(1):266.
262. Lee KY, Han IS, Go HY, Lee DN, Yu JS, Sun SH. Efficacy and safety of Onkyeong-tang in treating cold hypersensitivity in the feet of Korean women: protocol for a double-blind, randomized, placebo-controlled, parallel-group, multicenter clinical study. Trials. 2020;21(1):410.
263. Deng C, Lou Y, Gao Y, Deng B, Su F, Jia L. Efficacy and safety of Shengjiang Xiexin decoction in prophylaxis of chemotherapy-related diarrhea in small cell lung cancer patients: study protocol for a multicenter randomized controlled trial. Trials. 2020;21(1):370.
264. Zeng C, Liu X, Hu L, Feng Y, Xia N, Zeng H, et al. Jiao-tai-wan for insomnia symptoms caused by the disharmony of the heart and kidney: a study protocol for a randomized, double-blind, placebo-controlled trial. Trials. 2020;21(1):408.
265. Xu J, Wang R, You S, Zhang L, Zheng P, Ji G, et al. Traditional Chinese medicine Lingguizhugan decoction treating non-alcoholic fatty liver disease with spleen-yang deficiency pattern: Study protocol for a multicenter randomized controlled trial. Trials. 2020;21(1):512.
266. Wang H, She B, Mao B, Jiang H. Efficacy and safety of Zihua Wenfei granules in treatment of postinfectious cough (wind-cold invading lungs syndrome): study protocol for a randomized controlled trial. Trials. 2020;21(1):547.
267. Wang ZY, Fu SZ, Xu L, Li SS, Qian KJ, He XD, et al. Impact of Shenfu injection on a composite of organ dysfunction development in critically ill patients with coronavirus disease 2019 (COVID-19): A structured summary of a study protocol for a randomized controlled trial. Trials. 2020;21(1):738.
268. Yap NY, Loo WS, Zheng HF, Tan QM, Tan TK, Quek LYP, et al. A study protocol for HEalth-Related quality of life-intervention in survivors of Breast and other cancers experiencing cancer-related fatigue using TraditionAL Chinese Medicine: the HERBAL trial. Trials. 2020;21(1):909.
269. Wang Y, Huang YQ, Zhu SL, Zhang CR, Chen XL, Hou QK, et al. Efficacy of Tong-Xie-Yao-Fang granule and its impact on whole transcriptome profiling in diarrhea-predominant irritable bowel syndrome patients: study protocol for a randomized controlled trial. Trials. 2020;21(1):908.
270. Li Y, Liang L, Snellingen T, Xu K, Gao Y, Zhang F, et al. Mingjing granule, a traditional Chinese medicine in the treatment of neovascular age-related macular degeneration: study protocol for a randomized controlled trial. Trials. 2021;22(1):69.
271. Sin SH, Wu J, Kang Y, Yip KHK, Kong NS, Wan H, et al. Efficacy of modified Banxia Xiexin decoction in the management of Wei-Pi syndrome (postprandial distress syndrome): study protocol for a randomized, waitlist-controlled trial. Trials. 2021;22(1):135.
272. Zhang W, Xie Q, Xu X, Sun S, Fan T, Wu X, et al. Baidu Jieduan granules, traditional Chinese medicine, in the treatment of moderate coronavirus disease-2019 (COVID-19): study protocol for an open-label, randomized controlled clinical trial. Trials. 2021;22(1):1-7.
273. Ruan X, Li Y, Sun Y, Jia M, Xu X, Huo L, et al. Efficacy and safety of Suxiao Jiuxin Pill in the treatment of stable angina (Qi stagnation and blood stasis syndrome): study protocol of a randomized, double-blind, placebo-controlled, multi-center clinical trial. Trials. 2021;22(1):466.
274. Zhang X, Cheng Y, Li X, Tan X, Shi L, Shi X, et al. Efficacy and safety of the Chinese herbal formula Hewei Jiangni recipe for NERD with cold-heat complex syndrome: study protocol for a double-blinded randomized controlled trial. Trials. 2021;22(1).
275. Sun Y, Huang C, Huo L, Li Y, Chen J, Zhang Z, et al. Efficacy and safety of Qi-Jing Hui-Xin Decoction in the treatment of coronary microvascular angina: study protocol for a randomized, controlled, multi-center clinical trial. Trials. 2021;22(1):553.
276. Tian PP, Wu QJ, Li J, Chen HW, Wu J, Deng YW, et al. Efficacy and safety of Chinese herbal medicine Wen Xin

granules for the treatment of unstable angina pectoris with Yang deficiency and blood stasis syndrome: study protocol for a randomized controlled trial. Trials. 2021;22(1):798.
277. Du Y, Li YT, Fu XY, Li CJ, Lou YN. Efficacy of Guizhi Fuling Wan for primary dysmenorrhea: protocol for a randomized controlled trial. Trials. 2021;22(1).
278. Cao Y, Peijuan W, Lu Y, Chen Y, Chen S, Weibo Z. Effectiveness and safety of Bushen Huoxue granules in treatment of premature ovarian insufficiency: study protocol for a randomized, double-blinded, placebo-controlled, and multicenter clinical trial. Trials. 2021;22(1):877.
279. Ma Y, Cao X, Song J, Gao D, Wang X, Li L, et al. Effect of traditional Chinese medicine formula Guilu Xian on in vitro fertilization and embryo transfer outcome in older women with low prognosis: study protocol for a prospective, multicenter, randomized double-blind study. Trials. 2021;22(1):917.
280. Wang Y, Xu YH, Zhang L, Huang SW, Dou LP, Yang JH, et al. Comparison of Buyang Huanwu granules and Naoxintong capsules in the treatment of stable angina pectoris: rationale and design of a randomized, blinded, multicentre clinical trial. Trials. 2022;23(1).
281. Guo Y, Lu H, Gan J, Li D, Gao J, Zhang C. Efficacy of Chinese herbal medicine Jiangniaosuan formula for treatment of hyperuricemia: study protocol for a double-blinded non-inferiority randomized controlled clinical trial. Trials. 2022;23(1):1.
282. Wei Y, Huang YS, Yang Z, Wang X, Li Y, Zhang Y, et al. Effectiveness of the Shenzhuo formula in the treatment of patients with macroalbuminuria secondary to diabetic kidney disease: protocol update and statistical analysis plan. Trials. 2022;23(1).
283. Sun Y, Hu N, Chen G, Wang Y, Hu Y, Ge M, et al. Efficacy and safety of Qushi Huayu granule for hyperlipidemia: study protocol for a randomized, double-blind, placebo-controlled trial. Trials. 2022;23(1):104.
284. Liang SB, Han M, Cheng HJ, Zhang QY, Zhang NW, Jia BY, et al. Chinese herbal formula Tongxie Yaofang for diarrhea-predominant irritable bowel syndrome: study protocol for a randomized, multiple-blind, placebo-controlled trial. Trials. 2022;23(1):226.
285. He Y, Dai C, Shen J, Chen Q, Gao J, Pan X, et al. Effect of Baihu and Guizhi decoction in acute gouty arthritis: study protocol for a randomized controlled trial. Trials. 2022;23(1):317.
286. Jiang L, Fu Q, Wang S, Chen Y, Li J, Xiao Y, et al. Effect of RG (Coptis root and ginseng) formula in patients with type 2 diabetes mellitus: a study protocol for a randomized controlled and double-blinding trial. Trials. 2022;23(1):305.
287. Li YQ, Shen TT, Wang QY, Ma MX, Tian FY, She YY, et al. The efficacy and safety of Longmu Tang granule for the treatment of atopic dermatitis: study protocol for a single-centred, double-blinded, randomised, placebo-controlled trial. Trials. 2022;23(1).
288. Cheng G, She B, Mao B, Jiang H. Efficacy and safety of Tanreqing oral liquid in treatment of acute bronchitis: study protocol for a randomized controlled trial. Trials. 2022;23(1):373.
289. Gu Z, Jia Q, Cong J, Cen R, Chen Y, Wu C, et al. Efficacy and safety of Elian Granules in treating chronic atrophic gastritis: study protocol for a randomized, double-blind, placebo-controlled, multicenter clinical trial. Trials. 2022;23(1):437.
290. Xiao M, Zhong LLD, Lam WC, Zhao Y, Gwee KA, Holtmann G, et al. Zhizhu Kuanzhong Capsule in treating patients with functional dyspepsia postprandial distress syndrome: study protocol for a multicenter, randomized, double-blind, placebo-controlled, parallel-group clinical trial. Trials. 2022;23(1):454.
291. Yan J, Zhou Y, Yang Q, Wu J, He X. Evaluation of the safety and efficacy of a Fuling-Zexie decoction for people with asymptomatic hyperuricemia: protocol for a prospective, double-blinded, randomized, placebo-controlled clinical trial. Trials. 2022;23(1):517.
292. Li YX, Li JC, Tian M, Zheng MY, Zhang LP, Zhang JL, et al. Efficacy and safety of Dengyinnaotong Capsule in patients with Cognitive impairment caused by cerebral Small Vessel Disease: study protocol of a multicenter, randomized, open-label, controlled trial (De-CSVD trial). Trials. 2022;23(1):676.
293. Yang SG, Yu XQ, Li JS, Xie Y, Zhang W, Ban CJ, et al. Efficacy and safety of Jin-shui Huan-xian granule for idiopathic pulmonary fibrosis: study protocol for a multicenter, randomized, double-blind, placebo-controlled trial. Trials.

2022;23(1).
294. Guo SX, Li RB, Hu SY, Cai QH, Zhong CL, Hao RM. Efficacy and safety of Jiu-Wei-Xi-Feng granules for treating tic disorders in children: study protocol for a randomized controlled equivalence trial. Trials. 2022;23(1):898.
295. Long LZ, Chu JF, Qu H, Yang QN, Lu Y, Fu CG, et al. Effects of Qingda granule on patients with grade 1 hypertension at low-medium risk: study protocol for a randomized, controlled, double-blind clinical trial. Trials. 2023;24(1):1.
296. Hu XY, Oliver T, Willcox M, Simpson C, Thorne K, Trill J, et al. Treating Acute EXacerbations of COPD with Chinese HerbAL MedIcine to aid AntiBiotic Use Reduction (EXCALIBUR): study protocol of a randomised double-blind, placebo-controlled feasibility trial. Pilot Feasibility Stud. 2022;8(1):262.
297. Zhong C, Wu S, Chen C, Huang H, Mo J, Yang X, et al. Tiaochang Xiaoliu Decoction Granules prevent the recurrence of colorectal adenoma: a study protocol for a randomized controlled trial. Ann Palliat Med. 2021;10(4):4897-905.
298. Jiang X, Wang B, Chen X, Cao Z, Li K, Lin J, et al. Hantangping-a traditional Chinese medicine compound versus metformin for treating early type 2 diabetes mellitus: study protocol for a non-inferiority randomized controlled trial. 2021.
299. Jin M, Ren J, Ma J, Gao Z, Wei Y, Han L, et al. Hanshiyi Formula treatment in patients with COVID-19: study protocol and statistical analysis plan (SAP) of an open-label randomized controlled clinical trial. 2022.
300. Bi CR, Jing W, Xie XF, Liu YJ. Efficacy and mechanism of traditional Chinese medicine in relieving antibiotic-resistant bacterial diarrhea in children: study protocol for a randomized controlled trial. Trials. 2021;22(1):426.
301. Chen Y, He W, Lu W, Xing Y, Bai J, Yu H, et al. Bufei huoxue capsules in the management of convalescent COVID-19 infection: study protocol for a multicenter, double-blind, and randomized controlled trial. Pulmonary circulation. 2021;11(3):20458940211032125.
302. Wang L, Xiang L, Piao S, Gong X, Zhou W, Feng W, et al. The Efficacy and Safety of Chinese Medicine Fufang Zhenzhu Tiaozhi Capsule (FTZ) in the Treatment of Diabetic Coronary Heart Disease: Study Protocol for Multicenter, Randomized, Double-Blind, Placebo-Controlled Clinical Trial. Diabetes Metab Syndr Obes. 2021;14:2651-9.
303. Zhou L, Zhang Z, Li G, Liao S, Zhou H, Wang P, et al. Chinese Herbal Formula Xuefu Zhuyu for Tension-Type Headache with Qi -Stagnation and Blood-Stasis Pattern (CheruXTH): study Protocol for a Randomized Controlled Trial. Evidence-based complementary and alternative medicine. 2020;2020.
304. Gu Z, Wei G, Zhu L, Zhu L, Hu J, Li Q, et al. Preventive Efficacy and Safety of Yiqi-Wenjing-Fang Granules on Oxaliplatin-Induced Peripheral Neuropathy: a Protocol for a Randomized, Double-Blind, Placebo-Controlled, Multicenter Trial. Evidence-based complementary and alternative medicine : eCAM. 2021;2021:5551568.
305. Yu L, Lu X, Li X, Jiang H, Sun R, Chen G, et al. A Study Protocol for a Randomized, Double-Blind, Placebo-Controlled Clinical Study on the Effect of Qishen Yiqi Dripping Pills on Exercise Endurance and Quality of Life in Patients with Coronary Heart Disease after Percutaneous Coronary Intervention. Evidence-based complementary and alternative medicine : eCAM. 2021;2021:7439852.
306. Liu T, Lu BQ, Wang DD, Liao C, Chiang HJ, Zhang R, et al. Efficacy and Safety of Modified Yupingfeng Nasal Spray in Controlling the Recurrence of Persistent and Moderate-Severe Allergic Rhinitis: study Protocol for a Multicenter, Open-Label, Randomized, and Parallel-Arm Trial. Evidence-based complementary and alternative medicine. 2022;2022.
307. Zhang N, Zhou C, Wang LF, He TT, Wang Y, Wang Y, et al. Effectiveness and safety of Chinese herbal medicines for hepatitis B virus-related acute-on-chronic liver failure: study protocol for a multicenter randomized controlled trial. Journal of Traditional Chinese Medicine. 2020;40(6):1052-9.
308. Hu S, Qiuhan C, Xinmin L, ZHONG C, Youpeng W, Yongbin Y, et al. Efficacy and Safety of Susu Xiaoer Zhike Granules for Treating Acute Cough Due to Common Cold in Children: A Chinese Patent Medicine Study Protocol for Randomized Controlled Trial. 2021.
309. Zhang JJ, Sun Y, Zhou KH, Zhang XY, Chen Y, Hu JY, et al. Rationale and design of the AUGUST-AHF Study. Esc Heart Failure. 2020;7(5):3124-33.
310. Wu X, Guo M, Shi S, Shi S, Deng Y, Wang S, et al. Efficacy and Safety of Shenqisuxin Granule for Non-ST-segment Elevation Acute Coronary Syndrome: Study Protocol for a Randomized, Double-Blinded, Placebo-Controlled Trial.

Front Cardiovasc Med. 2022;9:888724.
311. Liu T, Yao S, Jiang W, Lan T, Xu W, Cao H, et al. Xin-Li-Fang efficacy and safety for patients with chronic heart failure: A study protocol for a randomized, double-blind, and placebo-controlled trial. Front Cardiovasc Med. 2023;10:1103548.
312. Sum CH, Ching JYL, Song T, Cheong PK, Lo CW, Lai MK, et al. Chinese medicine for residual symptoms of COVID-19 recovered patients (long COVID)-A double-blind, randomized, and placebo-controlled clinical trial protocol. Front Med (Lausanne). 2022;9:990639.
313. Liu X, Qin T, Li T, Shan L, Lei X, Xu X, et al. "Huoling Shengji granule" for amyotrophic lateral sclerosis: protocol for a multicenter, randomized, double-blind, riluzole parallel controlled clinical trial. Front Aging Neurosci. 2023;15:1153973.
314. Kim EH, Yoon J-H, Park SB, Lee JY, Chung WK, Yoon SW. Comparative Efficacy of Jaungo, A Traditional Herbal Ointment, and the Water-in-Oil Type Non-Steroidal Moisturizer for Radiation-Induced Dermatitis in Patients With Breast Cancer: A Study Protocol for a Prospective, Randomized, Single-Blinded, Pilot Study. Frontiers in Pharmacology. 2021;12:751812.
315. Tang Y, Li H, Huang L, Wang Q, Han Y, Wu H, et al. Yunpi Qufeng Chushi Formula for Pre-Rheumatoid Arthritis: Study Protocol for a Multiple-Center, Double-Blind, Placebo-Controlled Randomized Controlled Trial. Front Pharmacol. 2022;13:793394.
316. Wu H, Fang X, Jin D, Miao R, Wei J, Zhao T, et al. Efficacy and Mechanism of the Jiangtang Tiaozhi Recipe in the Management of Type 2 Diabetes and Dyslipidaemia: A Clinical Trial Protocol. Front Pharmacol. 2022;13:827697.
317. Zhang HW, Yeung KNK, Tong MCF, Lin ZX, Chang WWT, Ng IH, et al. A Chinese Medicine Formula (Bushen Huoxue Tongluo) for the Treatment of Chronic Subjective Tinnitus: A Study Protocol for a Pilot, Assessor-Blinded, Randomized Clinical Trial. Front Pharmacol. 2022;13:844730.
318. Zhang D, Li T, Wang A, Feng L, Lai X, Cao K, et al. Efficacy and safety of LongShengZhi capsule on functional recovery after acute ischemic stroke (LONGAN): Protocol and statistical analysis plan for a randomized, double-blind, placebo-controlled trial. Front Pharmacol. 2022;13:916421.
319. Xu J, Piao C, Qu Y, Liu T, Peng Y, Li Q, et al. Efficacy and mechanism of Jiedu Tongluo Tiaogan Formula in treating type 2 diabetes mellitus combined with non-alcoholic fatty liver disease: Study protocol for a parallel-armed, randomized controlled trial. Front Pharmacol. 2022;13:924021.
320. Liu Y, Chen X, Wang H, Yao C, Gou X, Gao Z, et al. Effectiveness and safety analysis of SanHanHuaShi granules for the treatment of coronavirus disease 2019: Study protocol and statistical analysis plan for a randomized, parallel-controlled, open-label clinical trial. Front Pharmacol. 2022;13:936925.
321. Kang J, Liu Y, Peng S, Tang X, Liu L, Xie Z, et al. Efficacy and safety of traditional Chinese medicine external washing in the treatment of postoperative wound of diabetes complicated with anal fistula: Study protocol of a randomized, double-blind, placebo-controlled, multi-center clinical trial. Front Pharmacol. 2022;13:938270.
322. Wan YY, Yang JX, Ma TY, Wang WQ, Wang HN, Sun WT, et al. A Chinese medicine formula (kunbixiao granule) for female rheumatoid arthritis: Study protocol for a double-blind, randomized, placebo-controlled trial (vol 13, 945565, 2022). Frontiers in Pharmacology. 2022;13.
323. Wu X, Zhou L, Dong H, Tian M, Liu S, Xu X. Efficacy, safety and mechanism of Honghua Xiaoyao Pill in the treatment of peri-menopausal syndrome: A study protocol for a randomized controlled trial. Front Pharmacol. 2022;13:1001228.
324. Cheong PK, Ho TM, Chan KL, Lo CW, Leung SB, Hon KL, et al. The efficacy and safety of Yupingfeng Powder with variation in the treatment of allergic rhinitis: Study protocol for a randomized, double-blind, placebo-controlled trial. Front Pharmacol. 2022;13:1058176.
325. Wang Y, Li J, Yan J, Wang Y, Cheng Y, Liu Z, et al. Efficacy of Xinbao pill on chronic heart failure: Study protocol of a multicenter, randomized, double-blind, placebo-controlled trial. Front Pharmacol. 2022;13:1058799.
326. Ni W, Liu T, Liu Y, Lu L, Zhou B, Dai Y, et al. Sijunzi decoction granules in the prevention and treatment of recurrence of colorectal adenoma: Study protocol for a multicenter, randomized, double-blind, placebo-controlled trial. Front

Pharmacol. 2023;14:1175811.
327. Hsu YT, Ng HY, Chen YH, Huang YC, Lee YY, Tsai MY. Assessing the efficacy and safety of Juan Bi Tang for dialysis-related myofascial pain in the fistula arm: Study protocol for a randomized cross-over trial. Front Public Health. 2022;10:925232.
328. Li Y, Han J, Wang J, He J, Zhang G, Liu L, et al. Traditional Chinese Medicine (Xiao Tan San Jie Granule) for Covid-19 Patients in Rehabilitation Stage: Study Protocol for a Randomized Controlled Trial. 2021.
329. Guo X, Xuan M, Zheng H, Qin S, Wu H, Huang S, et al. The Chinese herbal formula Huoxiang Zhengqi for diarrhea-predominant irritable bowel syndrome (CHAIRS): a study protocol for a double-blinded randomized controlled trial. Trials. 2021;22(1):491.
330. Lee B, Ha N-Y, Park H-J, Kim A-R, Kwon O-J, Cho J-H, et al., editors. Herbal Medicine Yukgunja-Tang for Functional Dyspepsia: A Protocol for a Randomized, Controlled, Multicenter Clinical Trial. Healthcare; 2023: MDPI.
331. Pan Y, Chang R, He Z, Hong M. How to prophylactically alleviate postembolization syndrome following transarterial chemoembolization?: protocol of a double blinded, randomized, placebo-controlled trial. Medicine. 2021;100(14):e25360.
332. Huanjia G, Hairong C, Jieqin Z, Xingzhen D, Xue F, Weizhang Z, et al. Efficacy and safety of Naoxintong capsule for treating chronic stable angina: study protocol for a randomized controlled trial. Trials. 2021;22(1):336.
333. Lee B, Park H-J, Jung S-Y, Kwon O-J, Park Y-C, Yang C. Herbal Medicine Maekmundong-Tang on Patients with Nonspecific Chronic Cough: Study Protocol for a Double-Blind, Randomized Controlled Clinical Trial. International Journal of Environmental Research and Public Health. 2023;20(5):4164.
334. Jieqin Z, Shuling L, Hairong C, Xingzhen D, Yanhong C, Zilin J, et al. Efficacy and safety of Guhong injection for treating coronary microvascular disease: study protocol for a randomized controlled trial. Trials. 2020;21(1):75.
335. Ziyi Z, Can W, Yuanqi Z, Xiangzhe L, Ying G, Hongwei AN, et al. Efficacy and safety of a sequential treatment with clearing heat and eliminating phlegm and tonifying and activating blood circulation in treating acute ischemic stroke: study protocol for a randomized controlled trial. J Tradit Chin Med. 2022;42(4):604-10.
336. Lin J, Yang T, Chen W, Qi X, Cao Y, Zheng X, et al. Zhengyuan capsules for the treatment of chemotherapy-induced cancer-related fatigue in stage IIIB-IV unresectable NSCLC: study protocol for a randomized, multi-center, double-blind, placebo-controlled clinical trial. J Thorac Dis. 2022;14(11):4560-70.
337. Lee B, Jeong YE, Park HJ, Choi YE, Kim H, Kim BY, et al. Effects of Sihogayonggolmoryeo-tang (Saikokaryukotsuboreito or Chai-Hu-Jia-Long-Gu-Mu-Li-Tang) for insomnia disorder with prehypertension or stage 1 hypertension: a study protocol for a randomized controlled trial. Medicine. 2020;99(29):e20980.
338. Leng Y, Gao H, Fu X, Xie H, Hu Z, Zhu J, et al. The efficacy and safety of Chinese herbal medicine Shen-Qi Hua-Yu formula in patients with diabetic lower extremity artery disease: study protocol of a multi-center, randomized, double-blind, placebo-controlled trial. Medicine. 2020;99(3):e18713.
339. Li S, Zhang C, Zhang HY, Zhou M, Wang SN, Xu R, et al. Efficacy and Safety of Jueyin Granules for Patients with Mild-to-Moderate Psoriasis Vulgaris: protocol for a Multicenter Randomized Placebo-Controlled Trial. Evidence-based complementary and alternative medicine. 2020;2020.
340. Li G, Zhang Z, Zhou L, Liao S, Sun J, Liu Y, et al. Chinese herbal formula Xuefu Zhuyu for primary dysmenorrhea patients (CheruPDYS): a study protocol for a randomized placebo-controlled trial. Trials. 2021;22(1):95.
341. Liao S, Zhang Z, Li G, Zhou L, Jiang J, Zhang N, et al. Chinese Herbal Formula Xuefu Zhuyu for Stable Angina (CheruSA): study Protocol for a Multicenter Randomized Controlled Trial. Evidence-based complementary and alternative medicine. 2020;2020.
342. Cheng YC, Wang YM, Lin YH, Cheng JY, Li SH, Huang YC, et al. Preventive effect and safety of Chinese herbal medicine for oral mucositis during radiotherapy in patients with head and neck cancer: Study protocol for a randomized trial. Contemp Clin Trials Commun. 2022;27:100912.
343. Kim K-I, Hong M, Park Y-C, Lee B-J, Kim K, Kang BK, et al. Herbal medicines (Eunkyosan and Samsoeum) for treating the common cold: a protocol for a randomized, placebo-controlled, multicenter clinical trial. Integrative Medicine Research. 2020;9(1):48-53.

344. Lu L, Xu L, He Y, Shen J, Xin J, Zhou J, et al. Evaluation the effectiveness of the Jiangniaosuan formulation in the treatment of hyperuricemic nephropathy in patients with chronic kidney disease stages 3-4: Study protocol of a randomized controlled trial. Contemp Clin Trials Commun. 2023;32:101065.
345. Xie L, Xie Y, Mao G, Jiang JL, Yao T, Fang R, et al. A randomized double-blind controlled study protocol on the efficacy and safety of Sangdantongluo granule in the treatment of post-stroke spasticity. Contemporary Clinical Trials Communications. 2022;29.
346. Liu R, Zhao Y, Wu Y, Guo M, Duan Y, Ye J, et al. Individualized Chinese medicine for the treatment of diabetic patients with dry eye disease: a single-case randomized controlled protocol. Medicine. 2020;99(1):e18459.
347. Yang H, Zhang C, Gan W, Chen J, Wu J, Xiao W, et al. A randomized controlled trial study protocol for Xiao-Qing-Long decoction in the treatment of refractory asthma: study protocol clinical trial (spirit compliant). Medicine. 2020;99(5):e18911.
348. Shi SY, Zhou Q, He ZQ, Shen ZF, Zhang WX, Zhang D, et al. Traditional Chinese medicine (Liang-Xue-Di-Huang Decoction) for hemorrhoid hemorrhage: study Protocol Clinical Trial (SPIRIT Compliant). Medicine. 2020;99(16):e19720.
349. Pan X, Tao H, Nie M, Liu Y, Huang P, Liu S, et al. A clinical study of traditional Chinese medicine prolonging the survival of advanced gastric cancer patients by regulating the immunosuppressive cell population: a study protocol for a multicenter, randomized controlled trail. Medicine. 2020;99(16):e19757.
350. Zhou Z, Fu S, Liu Y, Wang Y, Bu H, Mei Y, et al. Study of efficacy and safety of Jiaotai pill in the treatment of depression. Medicine (Baltimore). 2020;99(18):e19999.
351. Wang K, Cai JJ, Wu Y, Wang Y, Liu LL, Shi L, et al. Prospective randomized controlled trial study of Luofengning granule in the treatment of unstable angina. Medicine (Baltimore). 2020;99(20):e20025.
352. Zhao K, Chen K, Huang Q, Gao P, Zhang C, Yang H, et al. Traditional Chinese medicine may reduce the dosage of systemic glucocorticoids in required patients with acute exacerbation of chronic obstructive pulmonary disease: Study protocol for a randomized placebo-controlled trial. Medicine. 2020;99(18).
353. Tian J, Zhang L, Yang X, Zuo H, Zhao X, Yong J, et al. The effect of Shexiang Tongxin Dropping Pills on coronary microvascular dysfunction (CMVD) among those with a mental disorder and non-obstructive coronary artery disease based on stress cardiac magnetic resonance images: a study protocol. Medicine. 2020;99(21):e20099.
354. Lin W, Yu Q, Qin Y, Dai L, Xiao J, Jiao L, et al. To explore the clinical efficacy of Traditional Chinese Medicine bath in the treatment of psoriasis vulgaris with blood-heat syndrome and its effect on related cytokines based on different temperature and different concentration. Medicine (Baltimore). 2020;99(19):e20172.
355. Guo LJ, Yuan H, Zhang DW, Zhang J, Hua Q, Ma XC, et al. A multi-center, randomized, double-blind, placebo-parallel controlled trial for the efficacy and safety of shenfuqiangxin pills in the treatment of chronic heart failure (Heart-Kidney yang deficiency syndrome). Medicine. 2020;99(21).
356. Wang BQ, Mei J, Liu L, Ju CX, Zhao JN, Zhang P, et al. Exploratory study on the safety and effectiveness of Yizhi Qingxin Decoction (capsules) in the treatment of hypertension in the elderly with mild cognitive impairment (deficiency of kidney essence syndrome). Medicine. 2020;99(27):e20789.
357. Jin Z, Liu Z, Kang L, Yang A, Zhao H, Yan X, et al. A randomized double-blind placebo-controlled multicenter trial of Bushen Yisui and Ziyin Jiangzhuo formula for constipation in Parkinson disease. Medicine (Baltimore). 2020;99(28):e21145.
358. Xu C, Zhou X, Tong Z, Ma J, Ye J, Xu J, et al. A randomized, double-blind, placebo-controlled trial for Yi-Qi Hua-Yu tong-sui granule in the treatment of mild or moderate cervical spondylotic myelopathy. Medicine (Baltimore). 2020;99(33):e21776.
359. Gan W, Huang Q, Wang M, Wang J, Hui Y, Zhao K, et al. A randomized controlled trial study protocol of modified Mahuang-Fuzi-Xixin decoction in the treatment of patients with mild bronchial asthma during acute exacerbation. Medicine. 2020;99(35):e21858.
360. Zhang Q, Fan L, Li F, Sun Z, Zhang C, Chen R. Yishentongluo decoction in treatment of idiopathic asthenozoospermia infertility: study protocol for a randomized controlled trial. Medicine. 2020;99(43):e22662.

361. Yang G, He HQ, Li HZ, Shen ZN, Zhou SY, Lu BX, et al. Effects of Danlou tablet for the treatment of stable angina pectoris A study protocol of a randomized, double-blind, and placebo-controlled clinical trial. Medicine. 2020;99(49).
362. Gan W, Huang Q, Xiao G, Luo Y, Wang J, Zhang C, et al. Modified Maimendong decoction in the treatment of patients with idiopathic pulmonary fibrosis: study protocol for a randomized controlled trial. Medicine. 2020;99(49):e23460.
363. Jing C, Zhou L, Ai J, Li Z, Wu J, Sun Y, et al. Peony and licorice decoction fumigation treatment for strephenopodia after stroke: study protocol for a randomized controlled pilot trial. Medicine. 2020;99(50):e23600.
364. Tan X, Zhao XJ, Li JX, Xie CE, Pei WJ, Shi L, et al. Study on the clinical mechanism of Tong-Xie-An-Chang Decoction in the treatment of diarrheal irritable bowel syndrome based on single-cell sequencing technology. Medicine (Baltimore). 2020;99(52):e23868.
365. Yang H, Liu X, Peng W, Chen R, Chen Y. Lipi Guben decoction in treating diarrheal irritable bowel syndrome: a study protocol for a randomized controlled trial. Medicine. 2021;100(3):e23887.
366. Ma Y, Zhang D, Lv Z, Cui Y, Fei Y, Chang T, et al. Optimal intervention time and risk of the activating blood and removing stasis method in acute cerebral hemorrhage patients: A randomized placebo-controlled trial. Medicine (Baltimore). 2021;100(2):e24214.
367. Li C, Fan W, Pan Z, Zheng G, Zhang Q, Rong J. Efficacy and safety of Buyang Huanwu decoction in the treatment of varicose veins of the lower extremities: a protocol of randomized controlled trial. Medicine. 2021;100(8):e24663.
368. Chen C, Xia J, Feng R, Wan J, Zhou K, Lin Q, et al. Randomized controlled clinical study on Yiqi Liangxue Shengji prescription for intervention cardiac function of acute myocardial infarction with ischemia-reperfusion injury. Medicine (Baltimore). 2021;100(10):e24944.
369. Zheng X, Wang W, Wang G, Liu S. Could Jinfukang alleviate the chemotherapy-related adverse effects in non-small cell lung cancer patients?: a protocol for a double-blind, randomized controlled trial. Medicine. 2021;100(28):e25002.
370. Wang R, Shi Y, Xie X, Ge Q, Xu J, Sun Q, et al. Use of Shenhuang paste on Shenque point improves chemotherapy induced gastrointestinal toxicity in breast cancer: a protocol for randomized controlled trial. Medicine. 2021;100(15):e25097.
371. Qiu B, Zhao P, Shen L, Qiao S, Li G, Deng B, et al. Improvement of Shen'ge formula on heart function in diastolic heart failure: a protocol for randomized, double-blind, placebo-controlled clinical study. Medicine. 2021;100(13):e25383.
372. Zhou Y, Wang W, Tian K, Huang H, Jia M. Efficacy and safety of Biqi capsule in the treatment of knee osteoarthritis: a protocol of a randomized controlled trial. Medicine. 2021;100(16):e25476.
373. Liang J, Tao X, Hu D, Cao Y. Efficacy and safety of Zhen Wu Decoction against chronic heart failure: a protocol of randomized, double-blinded, and controlled trial. Medicine. 2021;100(38):e27260.
374. Pei H, Ma Y, Wang L, Wang L, Xu L, Wang R. Effects of Shenfu injection on inflammatory factors and immune function in children with Mycoplasma pneumoniae: a protocol for a double-blind, randomized controlled trial. Medicine. 2021;100(42):e27585.
375. Gao J, Shao X, Guan Y, Mei J. Effect of Danhong injection on neurological recovery and adverse events in patients with acute ischemic stroke: a protocol for a randomized, double-blind, placebo-controlled clinical study. Medicine. 2021;100(46):e27683.
376. Yue B, Wang Y, Zhang C, Ding Y, Liu Z. Efficacy of Shaobei injection in the treatment of grade II-III hemorrhoids and the effect on fibulin protein expression: a study protocol of a randomized controlled trial. Medicine. 2021;100(46):e27706.
377. Hu J, Li X, Fang Y, Peng J. Efficacy and safety of Buzhong Yiqi Decoction in improving cancer-related fatigue and immunity of cervical carcinoma patients: A protocol of randomized controlled trial. Medicine. 2021;100(49):e27938.
378. Ji L, Zhao X, Zhang Y, Zhao P, Gong R, Li F, et al. Efficacy and safety of Qinghua Zhixie Decoction against diarrhea-

predominate irritable bowel syndrome: a protocol for a randomized controlled trial. Medicine. 2022;101(9):e28895.
379. Lee B, Park HJ, Jung SY, Kwon OJ, Ko MM, Jeong HA, et al. Effects of Bojungikgi-tang on anorexic patients with atopic dermatitis: a protocol for a randomized, usual care-controlled, assessor-blinded, parallel, pilot clinical trial. Medicine. 2022;101(9):e28965.
380. Li N, Zhang C, Wang Z, Zhang Q, Chen R, Hua Z, et al. Clinical study of Wuwei Fuzheng Yijing formula in the treatment of sperm DNA damage in male infertility: a study protocol for a randomized controlled trial. Medicine. 2022;101(43):e31226.
381. Ma M, Ju B, Li X, Yao J, Li L, Zhang Y, et al. Clinical study on the treatment of male infertility with Wuwei Fuzheng Yijing decoction based on microplastics: study protocol for a randomized controlled trial. Medicine. 2022;101(41):e31265.
382. Zhang X, Yang K, Wang S, Tang B, Yin H, Lei Q, et al. Efficacy and safety of Yaobitong capsule for acute lumbar disc herniation: a protocol for a multi-center randomized controlled trial. Medicine. 2022;101(47):e31533.
383. Qiao YN, Lin SZ, Duan XZ, Yang MH, Zhang XF, Li JJ, et al. A randomized, double-blind, placebo-controlled multicenter clinical trial of Xiehuang Jiejing granule in the treatment of cough variant asthma in children. Medicine (Baltimore). 2022;101(46):e31636.
384. Yang XD, Shi JX, Liao WC, Cui JY, Jin Z, Liu DL, et al. Intervention of Compound Xueshuantong Capsule on the incidence of heart failure in patients with acute myocardial infarction after PCI based on the combination of disease and syndrome: A multi-center, randomized, double-blind, controlled trial. Medicine (Baltimore). 2022;101(50):e32311.
385. Cheng R, Liu Q, Zhu Y, Zhao Y, Yang L, Zhang Q. Effectiveness of Jian-Pi-An-Tai formula for the pregnancy outcome of in vitro fertilization and embryo transfer in infertile women: Protocol of a randomized controlled trial. Medicine. 2022;101(51):e32419.
386. Wang Z, Du M, Li J, Shi D, Wang H, Li H, et al. Clinical study of Tongdu Shujin decoction in the treatment of ankylosing spondylitis with cold-dampness obstruction type: Study protocol for a randomized controlled trial. Medicine. 2023;102(6):e32852.
387. Ko MM, Kim P-W, Jung SY, Kim C-H, Lee B-C, Jung J. The clinical effects of Gyejibongnyeong-Hwan (Gui Zhi Fu Ling Wan) on patients with hyperlipidemia: A study protocol for a multicenter, double-blind, two-armed parallel, investigator-initiated, exploratory randomized controlled trial. Medicine. 2023;102(16):e33093.
388. Feng YW, Ning Lai, Yuen Kwan Agnes, Cheng Chien shan, Zhang Cheng, Li, Sha. Based on the theory of “body constitution of Chinese Medicine” and “combination of prescription and syndrome” to improve COVID-19 susceptible body constitution of residents in Hong Kong. 2020. Origin: ClinicalTrials.gov
389. Chong. HWHLT-YSL-NCH. The Effects of Traditional Chinese Medicine Gargle Solutions on the Oral Health of Leprosy Patients. 2021. Origin: ClinicalTrials.gov
390. Sun A. Clinical Observation Of ZhenQi Buxue Oral Liquid in Treating Menstrual Disorders. 2022. Origin: ClinicalTrials.gov
391. Xue Z, Huang Z, Cheng SL, Wang XH, Zhou X, Ma QY, et al. Efficacy and safety of Xiaoyao pills for mild to moderate depression: study protocol for a randomized controlled trial. Trials. 2022;23(1).
392. Chen Z, Yang X, Guo J, Jin T, Lin Z, Zhu P, et al. AGI grade-guided chaiqin chengqi decoction treatment for predicted moderately severe and severe acute pancreatitis (CAP trial): study protocol of a randomised, double-blind, placebo-controlled, parallel-group, pragmatic clinical trial. Trials. 2022;23(1):933.
393. Ying C, Xiao X, Xue C, Zheng S, He Q, Yao R, et al. The Effect of Qi-Shao-Tong-Mai-An-Shen (QSTMAS) Herbal Paste in Coronary Heart Disease Patients with Depression and/or Anxiety: A Randomized Controlled Clinical Trial Protocol. 2023.
394. Wu J, Li W, Ye B, Yao Y. The efficacy and safety of Xianling Gubao capsules in the treatment of knee osteoarthritis: a protocol for a randomized, double-blind, controlled trial. Medicine. 2021;100(36):e27086.
395. Pang B, Guo H, Zhang Y-y, Feng S, Hu H-j, Sun Y, et al. Traditional Chinese Patent Medicine Qizhijiangtang Capsule

for Non-Proliferative Diabetic Retinopathy: Study Protocol for a Randomized Controlled Trial. 2023.
396. Wei XM, Chen XF, Shu P, Jiang ZW, Wu XY, Zou X, et al. Study on efficacy and safety of Huangqi Guizhi Wuwu decoction treatment for oxaliplatin induced peripheral neurotoxicity: a protocol for a randomized, controlled, double-blind, multicenter trial. Medicine. 2020;99(22):e19923.
397. Woo HL, Ji HR, Kim S, Suh HS, Kim KI, Lee JM, et al. Efficacy and safety of herbal medicine (Dangguijagyag-san) for primary dysmenorrhea: study protocol for a randomized, double-blind, placebo-controlled, parallel-group, multi-center trial. Integrative Medicine Research. 2020;9(2).
398. Xu Y, Li X, Zhang H, Wu Y, Zhang J, Li J, et al. China Tongxinluo Study for myocardial protection in patients with Acute Myocardial Infarction (CTS-AMI): Rationale and design of a randomized, double-blind, placebo-controlled, multicenter clinical trial. Am Heart J. 2020;227:47-55.
399. Xu H, Wei X, Zhang R, Li L, Zhang Z, Jia R, et al. The acupoint herbal plaster for the prevention and treatment of postoperative nausea and vomiting after PLIF with general anesthesia: study protocol for a multicenter randomized controlled trial. Trials. 2021;22(1):79.
400. Xuan M, Guo X, Li H, Xie T, Mo X, Wen Z. The Chinese herbal formula Huoxiang Zhengqi for atopic dermatitis with dampness pattern (CHARM): a study protocol for a double-blinded randomized controlled trial. Trials. 2021;22(1):67.
401. Zhang T, Fei YT, Xu Y, Sun LY, He B, Yan SH, et al. Effect of Jianpi Bushen Sequential Formula on Adjuvant Chemotherapy of Colon Cancer: Study Protocol for a Randomized Controlled Trial. Chin J Integr Med. 2021;27(12):891-5.
402. Zhao Z, Wang X, Wang S, Zhou R, Su Q, Liu Y, et al. Research based on the core pathogenesis in the treatment according to traditional Chinese medicine syndrome differentiation for heart failure with normal ejection fraction. Medicine (Baltimore). 2020;99(37):e21663.
403. Ji L, Wang A, Fan Q, Zhang N, Weng L, Gu J. Prophylactic Moxibustion in Preventing Postoperative Urinary Retention of Hemorrhoidectomy: A Study Protocol for a Randomized Controlled Trial. Front Surg. 2022;9:898097.
404. Zhou YM, Luo Y, Yu ZY, Liao CX, Bai Y, Xiong Y, et al. Effectiveness of grain-sized moxibustion for rheumatoid arthritis: Study protocol for a randomized controlled trial. European Journal of Integrative Medicine. 2020;35.
405. Chen Y, Gu J, Wang Y. Efficacy and safety of mild-warm moxibustion in treating diarrhea-predominant irritable bowel syndrome (spleen deficiency and dampness excess syndrome): a study protocol for a randomized controlled trial. TMR Non-Drug Ther. 2023;6(2):7.
406. Wu C, Zhao L, Guo Y, Hao X, Fan Y, Wu P, et al. Moxibustion treatment for Parkinson's disease: study protocol for a randomized controlled trial. BMC Complement Med Ther. 2023;23(1):193.
407. Huang Y, Zhang J, Xiong B, Huang R, Zhao W, Zhou M, et al. Thunder-fire moxibustion for cervical spondylotic radiculopathy: Study protocol for a randomized controlled trial. Trials. 2020;21(1):143.
408. Zhou X, Li S, Li L, Deng G, Dai L, Chai L, et al. Community-based heat-sensitive moxibustion for primary hypertension: study protocol for a randomized controlled trial with patient-preference arms. Trials. 2022;23(1):154.
409. Liu Y, Wang P, Sun YY, Qu J, Li M. Efficacy of thunder-fire moxibustion in treating ankylosing spondylitis of kidney deficiency and governor meridian cold and its influence on TNF-α and RANKL: study protocol for a prospective, nonblinded, single-center, randomized controlled trial. Trials. 2022;23(1):344.
410. Yu B, Huang W, Zhang Y, Wang J, Xia C, Zhang Y, et al. Efficacy and Safety of Moxibustion for Menopausal Obesity: a Multicentre, Randomized, Controlled Trial Protocol. Evidence-based complementary and alternative medicine : eCAM. 2022;2022:9255017.
411. Xue K, Wang X, Quan F, Tang J, Wang X, Lan L, et al. Efficacy and safety of Ma's Bamboo-based medicinal moxibustion therapy for chronic fatigue syndrome: An exploratory study protocol for randomized controlled trial. Medicine: Case Reports and Study Protocols. 2022;3(1):e0193.
412. Zhang J, Zhai X, Wang X, Wang L, Tong H, Xian T, et al. The Effect of Thunder-Fire Moxibustion on Lumbar Disc Herniation: Study Protocol for a Randomized Controlled Trial. Front Public Health. 2022;10:930830.

413. Meng X, Wang L, Li C, Gao S, Yu H, Zhang L, et al. Efficacy of Moxibustion Smoke for Stage 1 Post-Stroke Shoulder-Hand Syndrome: Protocol for a Multi-Center, Single-Blind Randomized Sham-Controlled Trial. J Pain Res. 2022;15:643-53.
414. Iravani S, Cai L, Ha L, Zhou S, Shi C, Ma Y, et al. Moxibustion at 'Danzhong' (RN17) and 'Guanyuan' (RN4) for fatigue symptom in patients with depression: study protocol clinical trial (SPIRIT Compliant). Medicine. 2020;99(7):e19197.
415. Hu H, Jiang Y, Li X, Lou J, Zhang Y, He X, et al. The microcirculatory characteristics of the heart and lung meridians: Study protocol clinical trial (SPIRIT Compliant). Medicine. 2020;99(14):e19594.
416. Chen L, Huang Z, Cheng K, Wu F, Deng H, Lin L, et al. The efficacy of jade moxibustion in knee osteoarthritis. Medicine (Baltimore). 2020;99(17):e19845.
417. Zhou X, Wu Q, Zhang G, Wang Y, Li S, Wang B, et al. Heat-sensitive moxibustion self-administration in patients in the community with primary hypertension: a protocol for a multi-center, pragmatic, non-randomized trial. Medicine. 2020;99(38):e22230.
418. He J, Jiao L, Xu M, Gong R, Guo Z. A randomized controlled protocol on the effect of moxibustion on the cardiac function and quality of life in patients with chronic heart failure. Medicine. 2021;100(32):e26860.
419. Yu M, Yang S, Chen B, Gan L, He X, Wang A, et al. Effect of chrono-moxibustion and its influence on circadian rhythm for rheumatoid arthritis: a study protocol for a randomized controlled trial. Medicine. 2022;101(38):e30701.
420. Ma T, Gong R, Zheng R, Wu J. Dose-Effect of Long-Snake-Like Moxibustion for Chronic Fatigue Syndrome: Study Protocol for a Randomized Controlled Trial. 2021.

Supplementary file 5: Details of characteristics of included protocols

5.1 Journal type

Journal type	CHMF n (%)	Acupuncture n (%)	Moxibustion n (%)	Total n (%)
English journal (SCIE & ESCI), with impact factor <2 or none	71 (16.9)	62 (14.8)	14 (3.3)	147 (35.0)
English journal (SCIE & SSCI), with impact factor 2-3	63 (15.0)	129 (30.7)	6 (1.4)	198 (47.1)
English journal (SCIE & SSCI), with impact factor 3-5	19 (4.5)	37 (8.8)	1 (0.2)	57 (13.6)
English journal (SCIE), with impact factor >5	14 (3.3)	3 (0.7)	1 (0.2)	18 (4.3)

5.2 Distributions of corresponding authors

Distributions of corresponding authors	CHMF n (%)	Acupuncture n (%)	Moxibustion n (%)	Total n (%)
China	149 (35.5)	209 (49.8)	18 (4.3)	376 (89.5)
South Korea	9 (2.1)	8 (1.9)	0 (0)	17 (4)
USA	1 (0.2)	6 (1.4)	0 (0)	7 (1.7)
Germany	0 (0)	2 (0.5)	0 (0)	2 (0.5)
Brazil	0 (0)	2 (0.5)	0 (0)	2 (0.5)
France	0 (0)	2 (0.5)	0 (0)	2 (0.5)
Portugal	0 (0)	2 (0.5)	0 (0)	2 (0.5)
Poland	0 (0)	1 (0.2)	0 (0)	1 (0.2)
Australia	0 (0)	1 (0.2)	0 (0)	1 (0.2)
Canada	0 (0)	1 (0.2)	0 (0)	1 (0.2)
Singapore	1 (0.2)	0 (0)	0 (0)	1 (0.2)
UK	1 (0.2)	0 (0)	0 (0)	1 (0.2)

5.3 Type of disease/symptom

Type of disease/symptom	CHMF n (%)	Acupuncture n (%)	Moxibustion n (%)	Total n (%)
08 Diseases of the nervous system	14 (3.3)	47 (11.2)	5 (1.2)	66 (15.7)
11 Diseases of the circulatory system	39 (9.3)	9 (2.1)	4 (1)	52 (12.4)
15 Diseases of the musculoskeletal system or connective tissue	8 (1.9)	35 (8.3)	5 (1.2)	48 (11.4)
13 Diseases of the digestive system	22 (5.2)	20 (4.8)	1 (0.2)	42 (10)
21 Symptoms, signs or clinical findings, not elsewhere classified	10 (2.4)	30 (7.1)	0 (0)	40 (9.5)
02 Neoplasms	14 (3.3)	23 (5.5)	0 (0)	37 (8.8)

05 Endocrine, nutritional or metabolic diseases	14 (3.3)	17 (4)	1 (0.2)	32 (7.6)
06 Mental, behavioural or neurodevelopmental disorders	7 (1.7)	14 (3.3)	1 (0.2)	22 (5.2)
16 Diseases of the genitourinary system	14 (3.3)	17 (4)	0 (0)	21 (5)
07 Sleep-wake disorders	2 (0.5)	16 (3.8)	0 (0)	18 (4.3)
12 Diseases of the respiratory system	12 (2.9)	5 (1.2)	0 (0)	17 (4)
26 Supplementary Chapter Traditional Medicine Conditions - Module I	10 (2.4)	2 (0.5)	4 (1)	16 (3.8)
14 Diseases of the skin	8 (1.9)	7 (1.7)	0 (0)	15 (3.6)
22 Injury, poisoning or certain other consequences of external causes	0 (0)	14 (3.3)	1 (0.2)	15 (3.6)
01 Certain infectious or parasitic diseases	2 (0.5)	6 (1.4)	0 (0)	8 (1.9)
25 Codes for special purposes	8 (1.9)	0 (0)	0 (0)	8 (1.9)
09 Diseases of the visual system	3 (0.7)	1 (0.2)	0 (0)	4 (1)
04 Diseases of the immune system	1 (0.2)	0 (0)	0 (0)	1 (0.2)
10 Diseases of the ear or mastoid process	0 (0)	1 (0.2)	0 (0)	1 (0.2)
18 Pregnancy, childbirth or the puerperium	0 (0)	1 (0.2)	0 (0)	1 (0.2)

5.4 Type of TCM pattern(s)

**Type of TCM pattern(s)**	**CHMF** n（%）	**Acupuncture** **n (%)**	**Moxibustion** **n (%)**	**Total** **n (%)**
Qi stagnation and blood stasis syndrome	2 (0.4)	0(0)	0(0)	2 (0.4)
blood stasis syndrome	0(0)	1 (0.2)	0(0)	1 (0.2)
qi deficiency and blood stasis' syndrome	0(0)	1 (0.2)	0(0)	1 (0.2)
hyperactivity of liver yang	0(0)	1 (0.2)	0(0)	1 (0.2)
The disharmony of the heart and kidney	1 (0.2)	0(0)	0(0)	1 (0.2)
spleenyang deficiency pattern	1 (0.2)	0(0)	0(0)	1 (0.2)
wind-cold invading lungs syndrome	1 (0.2)	0(0)	0(0)	1 (0.2)
cold-heat complex syndrome	1 (0.2)	0(0)	0(0)	1 (0.2)
Yang deficiency and blood stasis syndrome	1 (0.2)	0(0)	0(0)	1 (0.2)
Heart-Kidney yang deficiency syndrome	1 (0.2)	0(0)	0(0)	1 (0.2)
deficiency of kidney essence syndrome	1 (0.2)	0(0)	0(0)	1 (0.2)
cold- dampness obstruction type	1 (0.2)	0(0)	0(0)	1 (0.2)
dampness pattern	1 (0.2)	0(0)	0(0)	1 (0.2)
spleen deficiency and dampness excess syndrome	0(0)	0(0)	1 (0.2)	1 (0.2)
kidney deficiency and governor meridian cold	0(0)	0(0)	1 (0.2)	1 (0.2)
kidney Yin and Yang deficiency	0(0)	0(0)	1 (0.2)	1 (0.2)
Spleen-Kidney Yang deficiency	0(0)	0(0)	1 (0.2)	1 (0.2)

Supplementary file 6: The inter-rater agreement rate of quality assessment

6.1 The inter-rater agreement rate of CHMFs quality assessment

No.	Section/topic	Extension items NO.	Q No.	Kappa	Agreement rate
1	Title	1a	Q1	0.854	99.40%
		1b	Q2	0.869	98.20%
2	Background and Rationale	6a.1	Q3	0.888	93.90%
		6a.2	Q4	0.89	96.30%
		6b	Q5	0.744	93.90%
3	Objectives	7	Q6	1	100%
4	Eligibility Criteria	10a	Q7	0.952	99.40%
		10b	Q8	0.823	90.80%
		10c	Q9	0.574	87.70%
5	Interventions	11a.1-1A	Q10	0.956	97.50%
			Q11	0.884	92.00%
			Q12	0.929	96.30%
			Q13	0.964	97.50%
			Q14	0.940	95.70%
			Q15	0.951	95.70%
			Q16	0.962	98.20%
			Q17	0.968	98.20%
			Q18	1	100%
			Q19	0.965	98.20%
			Q20	0.965	98.20%
			Q21	0.953	97.50%
			Q22	0.927	96.30%
			Q23	1	100%
		11a.2-1A	Q24	0.953	97.00%
			Q25	0.956	98.80%
		11d.2	Q26	0.941	96.30%
6	Outcomes	12a	Q27	0.932	96.30%
		12b	Q28	0.955	97.50%
7	Data Collection Methods	18a	Q29	0.948	99.40%

6.2 The inter-rater agreement rate of acupuncture quality assessment

No.	Section/topic	Extension items NO.	Q No.	Kappa	Agreement rate
1	Title	1a	Q1	0.84	97.9%
		1b	Q2	0.798	99.6%
2	Background and Rationale	6a.1	Q3	0.612	91.2%
		6a.2	Q4	0.547	94.6%
		6b	Q5	0.381	96.2%
3	Objectives	7	Q6	0.784	97.9%

4	Eligibility Criteria	10a	Q7	0.663	99.2%
		10b	Q8	0.797	90.4%
		10c	Q9	0.295	96.2%
5	Interventions	11a.1-1B	Q10	0.657	93.3%
			Q11	0.859	92.5%
			Q12	0.861	92.5%
			Q13	0.857	91.2%
			Q14	0.872	94.1%
		11a.2-1B	Q15	0.716	92.9%
			Q16	0.834	95.4%
			Q17	0.855	92.5%
		11d.2	Q18	0.819	93.3%
6	Outcomes	12a	Q19	0.347	96.7%
		12b	Q20	0.576	97.9%
7	Data Collection Methods	18a	Q21	0.317	96.2%

6.3 The inter-rater agreement rate of moxibustion quality assessment

No.	Section/topic	Extension items NO.	Q No.	Kappa	Agreement rate
1	Title	1a	Q1	1	100%
		1b	Q2	1	100%
2	Background and Rationale	6a.1	Q3	0.58	88.89%
		6a.2	Q4	1	100%
		6b	Q5	0.42	94.44%
3	Objectives	7	Q6	1	100%
4	Eligibility Criteria	10a	Q7	1	100%
		10b	Q8	1	94.44%
		10c	Q9	1	100%
5	Interventions	11a.1-1C	Q10	0.42	94.44%
			Q11	0.37	88.89%
			Q12	1	100%
			Q13	1	100%
			Q14	0.66	94.44%
		11a.2-1C	Q15	1.00	100%
			Q16	1.00	100%
		11d.2	Q17	1.00	100%
6	Outcomes	12a	Q18	0.4	88.89%
		12b	Q19	1.00	88.89%
7	Data Collection Methods	18a	Q20	1.00	100%

Supplementary file 7: Reporting quality assessment of CHMF protocols

No.	Section/topic	Extension items	Questions for assessment	Fully reported (%)	Partially reported (%)	Not reported (%)	Not applicable (%)
1	Title	1a<br>Specify the patient population in terms of 1) a WM-defined disease, 2) a WM-defined disease with a specific TCM pattern, or 3) a TCM pattern.	Q1. Whether the diseases or patterns was accurately and specifically reported in the title?	160 (98.2)	0	3 (1.8)	——
		1b<br>Specify the intervention, in terms of 1) CHMF, 2) acupuncture, 3) moxibustion, or 4) other TCM therapy(s).	Q2. Whether the specific intervention was reported in the title?	149 (91.4)	3 (1.9)	11 (6.7)	——
2	Background and Rationale	6a.1<br>Provide the background and rationale of the research question with TCM theory.	Q3. Whether the rationale of TCM about CHMF intervention for diseases or TCM patterns was reported in the background/introduction?	12 (7.4)	15 (9.3)	92 (56.4)	——
		6a.2<br>Describe the rationale of the utilized TCM interventions with references.	Q4. Whether the rationale of CHMF intervention was reported in the background/introduction?	12 (7.4)	133 (82.1)	18 (11.0)	——
		6b<br>Describe the rationale and principle(s) for selecting comparators corresponding to certain interventions (i.e. CHMFs,	Q5. Whether the he rationale and principle(s) for selecting comparators corresponding to CHMF intervention was reported?	16 (9.8)	143 (88.3)	4 (2.5)	——

		acupuncture, moxibustion or other TCM interventions), considering 1) comparable with tested intervention; 2) success of blinding.					
3	Objectives	7<br>State the objectives or hypotheses regarding the specific TCM intervention for 1) a WM-defined disease, 2) a WM-defined disease with a specific TCM pattern or 3) a TCM pattern.	Q6. Whether the objectives or hypotheses regarding CHMF intervention was reported?	161 (98.8)	2 (1.2)	0	——
4	Eligibility Criteria	10a<br>State whether participants with a specific TCM pattern will be recruited, in terms of 1) diagnostic criteria, and 2) inclusion and exclusion criteria. All criteria utilized should be universally recognized, or reference(s) where detailed explanations can be found should be given.	Q7. If participants with a specific TCM pattern would be recruited, whether the TCM diagnostic criteria, inclusion and exclusion criteria and reference(s) was reported?	7 (4.3)	6 (3.7)	0	150 (92.0%)
		10b<br>Descriptions of the roles, qualifications and other relevant experience of the researchers (e.g., participant screeners, care providers, outcome assessors, data analysts) in TCM research are recommended.	Q8. Whether the roles, qualifications and other relevant experience of the researchers was reported?	3 (1.8)	98 (60.5)	62 (38.0)	——
		10c<br>Descriptions of the qualification and relevant experience of study center(s) involved in a TCM trial are recommended.	Q9. Whether the qualification and relevant experience of study center(s) involved in a TCM trial was described?	1 (0.6)	140 (86.4)	22 (13.5)	——

5	Interventions	11a.1 Interventions for the experimental group(s) with sufficient detail to allow replication.	Q10. Whether the name of the CHMF and each medical substance was reported?	87 (53.4)	12 (7.4)	3 (1.8)	61 (37.4)
			Q11. Whether the source of the CHMF was reported?	35 (21.5)	2 (1.2)	62 (38.0)	64 (39.3)
			Q12. Whether the source and proccessing method of the CHMF were reported?	0	4 (2.5)	97 (59.5)	62 (38.0)
			Q13. Whether the dosage form, production method and administration route of the CHMF were reported?	38 (23.3)	56 (34.6)	6 (3.7)	63 (38.7)
			Q14. Whether the dosage of CHMF and each medical substance was reported in?	42 (25.8)	42 (25.9)	18 (11.0)	61 (37.4)
			Q15. Whether the reference(s) to dosage of CHMF was reported?	1 (0.6)	2 (1.2)	99 (60.7)	61 (37.4)
			Q16. Whether the administration route of the CHMF was reported in?	1 (0.6)	0	100 (61.3)	62 (38.0)
			Q17. Whether the quality control of each ingredient and the whole formula(s) and the safety assessment of the whole formula(s) were conducted?	11 (6.7)	3 (1.9)	87 (53.4)	62 (38.0)
			Q18. For protocol with individualized CHMFs, whether it reported how,	2 (1.2)	0	0	161 (98.8)

		when, and by whom the CHMF was modified?				
		Q19. For protocol with patent proprietary CHMFs, whether the name and dosage of formula were reported?	47 (28.8)	13 (8.0)	0	103 (63.2)
		Q20. For protocol with patent proprietary CHMFs, whether the efficacy of formula was reported?	11 (28.8)	47 (29.0)	2 (1.2)	103 (63.2)
		Q21. For protocol with patent proprietary CHMFs, whether the safety assessment and quality control of formula were reported?	5 (3.1)	6 (3.7)	48 (29.4)	104 (63.8)
		Q22. For protocol with patent proprietary CHMFs, whether the details of the formula were illustrated?	1 (0.6)	4 (2.5)	54 (33.1)	104 (63.8)
		Q23. Whether the patent proprietary CHMF utilized in the protocol is identical to the publiclyavailable reference?	0	0	61 (37.4)	102 (62.6)
	11a.2<br>Describe interventions for the control group(s) with sufficient detail to allow replication.	Q24. For protocol with placebo control, whether the name and dosage of each ingredient, the similarity of placebo with	39 (23.9)	76 (46.9)	6 (3.7)	42 (25.8)

			the intervention (e.g., color, smell, taste, appearance, packaging), the quality control and safety assessment of placebo,the dministration route, regimen, and dosage and the production information of placebo,including when, where, how, and by whom the placebo was produced were reported?				
			Q25. For protocal with active control, if a CHMF will be used, refer to the recommen-dations of 11a.1A; if a chemical agent will be used, whether the name,administration route, dosage and regime were reported?	1 (0.6)	24 (14.8)	0	138 (84.7)
		11d.2 Descriptions of other interventions that will be administrated to experimental and/or control groups are recommended (e.g., rescue interventions), with enough details to allow replication.	Q26. Whether the details of other interventions administered to experimental and/or control groups were reported?	67 (41.1)	16 (9.9)	6 (3.7)	74 (45.4)
6	Outcomes	12a Provide the rationale of TCM-related indexes as outcomes (e.g. the change of degree and scope of	Q27. Whether the rationale of TCM-related indexes as outcomes were reported?	18 (11.0)	39 (24.1)	1 (0.6)	105 (64.4)

		symptoms and signs related to pattern differentiation).<br>12b<br>Provide the details of the TCM-related outcomes assessment, including i) the measuring methods and standard (e.g. frequency, severity rating scale of symptoms and signs, verified pattern questionnaire, time points for assessment and corresponding rationale), ii) assessor qualification (e.g. relevant assessment experience, years in clinical practice), iii) methods used to enhance the quality of assessment (e.g. multiple repeated observation, training of assessors), and iv) related reference(s).	Q28. Whether the details of the TCM-related outcomes assessment were described?	35 (21.5)	6 (3.7)	21 (12.9)	101 (62.0)
7	Data Collection Methods	18a<br>When trial targeting TCM pattern, or a WM- defined disease with a specific TCM pattern, baseline data about TCM pattern should be provided.	Q29. If trial targeting TCM pattern, or a WM- defined disease with a specific TCM pattern, whether the baseline data about TCM pattern was provided?	2 (1.2)	3 (1.9)	5 (3.1)	153 (93.9)

Supplementary file 8: Reporting quality assessment of acupuncture protocols

No.	Section/topic	Extension items	Questions for assessment	Fully reported (%)	Partially reported (%)	Not reported (%)	Not applicable (%)
1	Title	1a<br>Specify the patient population in terms of 1) a WM-defined disease, 2) a WM-defined	Q1. Whether the diseases or patterns was accurately and specifically reported in the title?	224 (93.7)	7 (2.9)	8 (3.3)	—

		disease with a specific TCM pattern, or 3) a TCM pattern.<br>1b<br>Specify the intervention, in terms of 1) CHMF, 2) acupuncture, 3) moxibustion, or 4) other TCM therapy(s).	Q2. Whether the specific intervention was reported in the title?	237 (99.2)	1 (0.4)	1 (0.4)	—
2	Background and Rationale	6a.1<br>Provide the background and rationale of the research question with TCM theory.	Q3. Whether the rationale of TCM about acupuncture intervention for diseases or TCM patterns was reported in the background/introduction?	15 (6.3)	10 (4.2)	214 (89.5)	—
		6a.2<br>Describe the rationale of the utilized TCM interventions with references.	Q4. Whether the rationale of acupuncture intervention was reported in the background/introduction?	230 (96.2)	6 (2.5)	3 (1.3)	—
		6b<br>Describe the rationale and principle(s) for selecting comparators corresponding to certain interventions (i.e. CHMFs, acupuncture, moxibustion or other TCM interventions), considering 1) comparable with tested intervention; 2) success of blinding.	Q5. Whether the he rationale and principle(s) for selecting comparators corresponding to acupuncture intervention was reported?	12 (5.0)	227 (95.0)	0	—
3	Objectives	7<br>State the objectives or hypotheses regarding the specific TCM intervention for 1) a WM-defined disease, 2) a WM-defined	Q6. Whether the objectives or hypotheses regarding acupuncture intervention was reported?	228 (95.4)	7 (2.9)	4 (1.7)	—

		disease with a specific TCM pattern or 3) a TCM pattern.					
4	Eligibility Criteria	10a<br>State whether participants with a specific TCM pattern will be recruited, in terms of 1) diagnostic criteria, and 2) inclusion and exclusion criteria. All criteria utilized should be universally recognized, or reference(s) where detailed explanations can be found should be given.	Q7. If participants with a specific TCM pattern would be recruited, whether the TCM diagnostic criteria, inclusion and exclusion criteria and reference(s) was reported?	1 (0.4)	2 (0.8)	0	236 (98.7)
		10b<br>Descriptions of the roles, qualifications and other relevant experience of the researchers (e.g., participant screeners, care providers, outcome assessors, data analysts) in TCM research are recommended.	Q8. Whether the roles, qualifications and other relevant experience of the researchers was reported?	89 (37.2)	145 (60.7)	5 (2.1)	—
		10c<br>Descriptions of the qualification and relevant experience of study center(s) involved in a TCM trial are recommended.	Q9. Whether the qualification and relevant experience of study center(s) involved in a TCM trial was described?	1 (0.4)	233 (97.5)	5 (2.1)	—
5	Interventions	11a.1<br>Interventions for the experimental group(s) with sufficient detail to allow replication.	Q10. Whether the treatment environment and participant posture was reported?	1 (0.4)	30 (12.6)	208 (87.0)	—
			Q11. Whether the number of needle insertions per subject per session (mean	125 (52.3)	105 (43.9)	9 (3.8)	—

		and range if possible) and names and locations of acupoints (uni/bilateral) was reported?				
		Q12. Whether the angle and depth of insertion was reported?	63 (26.4)	90 (37.7)	86 (36.0)	—
		Q13. Whether the response sought from participants (e.g., de qi or muscle twitch response) and the needle stimulation (e.g., manual, electrical) was described?	112 (46.9)	37 (15.5)	90 (37.7)	—
		Q14. Whether the needle type (e.g. diameter, length, and manufacturer or material) was described?	172 (72.0)	29 (12.1)	38 (15.9)	—
		Q15. Whether the number of acupuncture treatment sessions, the frequency and duration of acupuncture treatment sessions and the needle retention time was reported?	211 (88.3)	16 (6.7)	12 (5.0)	—
	11a.2<br>Describe interventions for the control group(s) with sufficient detail to allow replication.	Q16. If the study protocol was the blank/waitlist control, whether any special arrangements in pre-treatment, treatment and post-treatment periods corresponding to the	6 (2.5)	31 (13.0)	1 (0.4)	201 (84.1)

			experimental intervention was reported ? Q17. If the study protocol was sham acupuncture or acupuncture-like control, whether the comparability of the sham acupuncture or acupuncture-like control and the comprehensive details as for the recommendations of Intervention 11a.1B were reported?	16 (6.7)	128 (53.6)	1 (0.4)	93 (38.9)
		11d.2 Descriptions of other interventions that will be administrated to experimental and/or control groups are recommended (e.g., rescue interventions), with enough details to allow replication.	Q18. Whether the details of other interventions administered to experimental and/or control groups were reported?	43 (18.0)	11 (4.6)	2 (0.8)	182 (76.2)
6	Outcomes	12a Provide the rationale of TCM-related indexes as outcomes (e.g. the change of degree and scope of symptoms and signs related to pattern differentiation).	Q19. Whether the rationale of TCM-related indexes as outcomes were reported?	0	5 (2.1)	233 (97.5)	—
		12b Provide the details of the TCM-related outcomes assessment, including i) the measuring methods and standard (e.g. frequency, severity rating scale of symptoms and signs, verified	Q20. Whether the details of the TCM-related outcomes assessment were described?	2 (0.8)	3 (1.3)	234 (97.9)	—

		pattern questionnaire, time points for assessment and corresponding rationale), ii) assessor qualification (e.g. relevant assessment experience, years in clinical practice), iii) methods used to enhance the quality of assessment (e.g. multiple repeated observation, training of assessors), and iv) related reference(s).					
7	Data Collection Methods	18a<br>When trial targeting TCM pattern, or a WM- defined disease with a specific TCM pattern, baseline data about TCM pattern should be provided.	Q21. If trial targeting TCM pattern, or a WM- defined disease with a specific TCM pattern, whether the baseline data about TCM pattern was provided?	0	0	3 (1.3)	236 (98.7)

Supplementary file 9: Reporting quality assessment of moxibustion protocols

No.	Section/topic	Extension items	Questions for assessment	Fully reported (%)	Partially reported (%)	Not reported (%)	Not applicable (%)
1	Title	1a<br>Specify the patient population in terms of 1) a WM-defined disease, 2) a WM-defined disease with a specific TCM pattern, or 3) a TCM pattern.	Q1. Whether the diseases or patterns was accurately and specifically reported in the title?	17 (94.4)	0	1 (5.6)	——
		1b<br>Specify the intervention, in terms of 1) CHMF, 2) acupuncture, 3) moxibustion, or 4) other TCM therapy(s).	Q2. Whether the specific intervention was reported in the title?	17 (94.4)	0	1 (5.6)	——
2	Background and Rationale	6a.1	Q3. Whether the rationale of TCM about moxibustion intervention for diseases or	3 (16.7)	8 (44.4)	7 (38.9)	——

		Provide the background and rationale of the research question with TCM theory.	TCM patterns was reported in the background/introduction?				
		6a.2<br>Describe the rationale of the utilized TCM interventions with references.	Q4. Whether the rationale of moxibustion intervention was reported in the background/introduction?	18 (100.0)	0	0	——
		6b<br>Describe the rationale and principle(s) for selecting comparators corresponding to certain interventions (i.e. CHMFs, acupuncture, moxibustion or other TCM interventions), considering 1) comparable with tested intervention; 2) success of blinding.	Q5. Whether the he rationale and principle(s) for selecting comparators corresponding to moxibustion intervention was reported?	7 (38.9)	11 (61.1)	0	——
3	Objectives	7<br>State the objectives or hypotheses regarding the specific TCM intervention for 1) a WM-defined disease, 2) a WM-defined disease with a specific TCM pattern or 3) a TCM pattern.	Q6. Whether the objectives or hypotheses regarding moxibustion intervention was reported?	17 (94.4)	0	1 (5.6)	——
4	Eligibility Criteria	10a<br>State whether participants with a specific TCM pattern will be recruited, in terms of 1) diagnostic criteria, and 2) inclusion and exclusion criteria. All criteria utilized should be universally recognized, or reference(s) where detailed explanations can be found should be given.	Q7. If participants with a specific TCM pattern would be recruited, whether the TCM diagnostic criteria, inclusion and exclusion criteria and reference(s) was reported?	1 (5.6)	3 (16.7)	0	14 (77.8)

		10b Descriptions of the roles, qualifications and other relevant experience of the researchers (e.g., participant screeners, care providers, outcome assessors, data analysts) in TCM research are recommended.	Q8. Whether the roles, qualifications and other relevant experience of the researchers was reported?	7 (38.9)	3 (16.7)	8 (44.4)	——
		10c Descriptions of the qualification and relevant experience of study center(s) involved in a TCM trial are recommended.	Q9. Whether the qualification and relevant experience of study center(s) involved in a TCM trial was described?	17 (94.4)	0	1 (5.6)	——
5	Interventions	11a.1 Interventions for the experimental group(s) with sufficient detail to allow replication.	Q10. Whether the patient posture during the moxibustion treatment and the treatment environment were reported?	3 (16.7)	8 (44.4)	7 (38.9)	0
			Q11. Whether the name and number (uni/bilateral) of acupoints/locations used for moxibustion was reported?	8 (44.4)	10 (55.6)	0	0
			Q12. Whether the moxibustion procedures and responses sought from participants (e.g., warm feeling, skin reddening, burning pain, heat-sensitization phenomenon, etc.) was reported?	11 (61.1)	7 (38.9)	0	0

		Q13. Whether the materials used for moxibustion was reported?	11 (61.1)	3 (16.7)	4 (22.2)	0
		Q14. Whether the number, frequency and duration of the moxibustion sessions were reported?	8 (44.4)	10 (55.6)	0	0
	11a.2 Describe interventions for the control group(s) with sufficient detail to allow replication.	Q15. If the study protocol was the blank/waitlist control, whether any special arrangements in pre-treatment, treatment and post-treatment periods corresponding to the experimental intervention was reported?	2 (11.1)	0	1 (5.6)	15 (83.3)
		Q16. If the study protocol was sham moxibustion or moxibustion-like control, whether the comparability of the sham moxibustion or moxibustion-like control and the comprehensive details as for the recommendations of Intervention 11a.1B were reported?	1 (5.6)	1 (5.6)	0	16 (88.9)
	11d.2 Descriptions of other interventions that will be administrated to experimental and/or control groups are recommended (e.g., rescue	Q17. Whether the details of other interventions administered to experimental and/or control groups were reported?	3 (16.7)	1 (5.6)	0	14 (77.8)

		interventions), with enough details to allow replication.					
6	Outcomes	12a<br>Provide the rationale of TCM-related indexes as outcomes (e.g. the change of degree and scope of symptoms and signs related to pattern differentiation).	Q18. Whether the rationale of TCM-related indexes as outcomes were reported?	3 (16.7)	3 (16.7)	12 (66.7)	——
		12b<br>Provide the details of the TCM-related outcomes assessment, including i) the measuring methods and standard (e.g. frequency, severity rating scale of symptoms and signs, verified pattern questionnaire, time points for assessment and corresponding rationale), ii) assessor qualification (e.g. relevant assessment experience, years in clinical practice), iii) methods used to enhance the quality of assessment (e.g. multiple repeated observation, training of assessors), and iv) related reference(s).	Q19. Whether the details of the TCM-related outcomes assessment were described?	2 (11.1)	3 (16.7)	13 (72.2)	——
7	Data Collection Methods	18a<br>When trial targeting TCM pattern, or a WM- defined disease with a specific TCM pattern, baseline data about TCM pattern should be provided.	Q20. If trial targeting TCM pattern, or a WM- defined disease with a specific TCM pattern, whether the baseline data about TCM pattern was provided?	1 (5.6)	2 (11.1)	1 (5.6)	14 (77.8)
